# Principles, concepts, and perspectives for the improvement of decompression procedures: a Neo-Haldanian approach bridging bubble physics and decompression physiology

**DOI:** 10.3389/fphys.2026.1943519

**Published:** 2026-10-07

**Authors:** Jean-Pierre Imbert, Ran Arieli, Costantino Balestra

**Affiliations:** 1 Environmental and Occupational, Physiology Laboratory, Haute Ecole Bruxelles-Brabant (HE2B), Brussels, Belgium; 2 The Israel Naval Medical Institute, Haifa, Israel; 3 Eliachar Research Laboratory, Western Galilee Medical Center, Nahariya, Israel; 4 Europe Research Division, Divers Alert Network (DAN), Brussels, Belgium; 5 Europe Research Division, Divers Alert Network (DAN), Roseto, Italy

**Keywords:** active hydrophobic site, arterial gas bubble, decompression algorithm, decompression sickness, decompression stress, inflammation, genetic, microbubble

## Abstract

**Introduction:**

This paper is a narrative review and theoretical perspective presenting principles, concepts, and perspectives for improving decompression procedures. It distinguishes three levels of evidence: established observations, hypotheses with experimental support, and mechanistic scenarios that remain hypothetical.

**Methods:**

We organize decompression around two linked dimensions: bubbles as the primary physical event and microparticles (MPs) as a physiological consequence. We hypothesize that a substantial proportion of decompression-related microbubbles originate from pre-existing gas micronuclei associated with active hydrophobic sites (AHS) at three locations: distal arterial, venous, and extravascular. From these assumptions, five bubble scenarios are considered: growth and detachment from distal arterial AHS; growth and detachment from venous AHS; pulmonary bubble filtration; survival of arterial gas microbubbles and their potential target organs; and intravascular bubble development. Physiological factors may generate MPs, endothelial dysfunction, systemic inflammation, and possibly a secondary generation of microbubbles.

**Results and discussion:**

The proposed scenarios align with decompression sickness (DCS) pathophysiology across five target organ systems: skin, brain, inner ear, spinal cord, and musculoskeletal tissue. Inflammatory stress appears important for defining post-dive intervals and multiday diving patterns. Designing decompression procedures therefore requires integrating individual variability and dive history; dive characteristics such as rapid ascents, yo-yo diving, and surface decompression; the bubble scenarios triggered during ascent; and accumulated oxidative stress, oxy-inflammation, and endothelial damage that modify susceptibility to DCS.

**Conclusion:**

There is no single ultimate decompression algorithm; rather, different strategies reflect different experience, assumptions, and operational contexts.

## 1 Introduction

Commercial divers work worldwide from diving support vessels, tunnel workers face increasing intervention pressures and saturation exposures; and technical recreational divers using rebreathers, propulsors, and dive computers now explore to 200 m of seawater (msw) and beyond.

New projects and equipment require new decompression procedures, combining scientific principles and mathematical modeling with empirical assumptions to solve immediate operational problems.

While sciences and technology differ in their objectives and methods, they evolve together. This evolution is illustrated by the safety performance of saturation procedures used in offshore commercial diving over the last 40 years. Comex, a historical diving company, had a decompression sickness (DCS) incidence of about 1% in 1977, which decreased to about 0.5% in 1992 (Imbert and Bontoux, 1988). Today, diving companies operating in the North Sea consider saturation DCS a rare event (Imbert et al., 2024b).

The industry culture strongly influences this evolution. The company’s safety management system controls the different steps of the new procedures validation, and external bodies (clients, authorities, and international industry standards) require documented justifications for their acceptance (Imbert et al., 2026).

Traditions also influence decompression procedures. Haldane’s 1908 publication and his historical algorithm have shaped the way we theorize decompression today (Boycott et al., 1908). Today, “Haldanian algorithms” underlie commercial diving tables (French MT92, US Navy tables) and most recreational dive computers.

Because decompression practice remains Haldanian, we present an alternative view bridging the physical and physiological factors governing safe decompression.

We first address design principles, scientific concepts, and mathematical modeling, then propose a theoretical construction that:

1. Identifies physical and physiological scenarios for the different manifestations of DCS.
2. Lists concepts, implications, proposed models, and perspectives.
3. Separates proven facts from perspectives to support innovative but practical reflection.
4. Assembles these elements into a “Neo-Haldanian” framework for decompression procedures.

This is a narrative review and theoretical perspective, not a systematic review. Rather than another enumeration of decompression concepts, it provides a structure in which physical and physiological literatures can be read together.

We use four labels with different standing:

- “Concept” reports published work.
- “Implication” and “Perspective” are our interpretation of it.
- “Proposed model” is a hypothesis that has not been implemented or validated.

Three levels of evidence are identified throughout:

- Level 1 covers well-established observations: venous gas emboli (VGE) after certain exposures, interindividual variability, post-dive endothelial and inflammatory activation, right-to-left shunts, and dive characteristics associated with DCS risk.
- Level 2 covers experimentally supported but unproven hypotheses: active hydrophobic sites (AHS), dipalmitoylphosphatidylcholine (DPPC) as their preferential substrate, pre-existing gas micronuclei, and the relation between site density and bubbling propensity.
- Level 3 covers largely hypothetical mechanisms: distal arterial sites in humans, microbubble detachment, preferential organ targeting, detailed cerebral, spinal and vestibular DCS mechanisms, and secondary micronuclei generation by microparticles (MPs).

This narrative review and theoretical proposal has several limitations:

- Method. No protocol, formal search strategy, or quality appraisal was used. Sources were selected for relevance from PubMed, Scopus, Google Scholar, UHMS/EUBS proceedings, and commercial-diving technical reports; results were not pooled.
- Levels of evidence. The framework combines all three levels above. Level 3 material should be read as testable scenarios and does not justify changes in practice.
- Operational observations. Some material derives from one author’s unpublished commercial-diving experience. It is anecdotal, was not collected under a protocol, and is identified where used; it documents practice but is not evidence of mechanism.
- The framework has not been implemented, calibrated, or validated against outcomes and must not be used to compute decompression. AHS density and the local oxygen window (OW) cannot currently be measured in divers; they are therefore experimental variables with testable predictions.

Finally, we exclude mechanisms such as barotrauma, which causes alveolar ruptures and produces a massive arterial gas embolization that generates rapid symptoms. Algorithms cannot account for critical arterial gas embolism (AGE) situations that are part of the definition of decompression illness (DCI) (Mitchell et al., 2022). For the same reason, this discussion excludes cardiopulmonary DCS and fulminant DCS (hemoconcentration, shock, and coagulopathy).

Note: Depth and chamber pressures are expressed in msw; 1 msw = 10.20 kPa gauge pressure, per EN 13319.

## 2 Principles

### 2.1 Models

Decompression theories provide conceptual descriptions of the decompression process, known as models.

Decompression algorithms translate models into a mathematical form suitable for computing a decompression schedule. The merit of an algorithm is appreciated through the performance of the tables it generates during its validation period.

A decompression plan defines the decompression method [in-water decompression, surface decompression, or transfer under pressure (TUP) decompression], the gas mix sequence (travel mix, bottom mix, and deco mixes), and the underwater breathing equipment (open-circuit, closed-circuit, semi-closed circuit, gas reclaim loop).

From a decompression plan and specified bottom depth and time, an algorithm produces a decompression procedure, either as a predefined schedule (tables), a decompression protocol (deco planners), or a real-time ascent profile (dive computers).

Models are designed to cover specific diving activities, which define the domain for which they are tested and validated. This is why Navy divers, offshore divers, and hyperbaric workers do not share tables. The risks may be the same, but the diving procedures and the operating controls differ.

### 2.2 The principle of parsimony

Adding conservatism and undocumented or proprietary adjustments can obscure a model’s original assumptions. We therefore distinguish descriptive models, which use multiple fitted parameters and interpolate efficiently within their validation domain, from explanatory models, which seek broader prediction from fewer, more meaningful assumptions.

- Descriptive models, which can be quite efficient but require multiple parameters to adjust their predictions. They are limited to their validation domain but can perform efficient interpolations.
- Explanatory models, which are based on more significant or relevant assumptions but fewer parameters. They are capable of efficient extrapolations.

The principle of parsimony holds that a multiplicity of parameters should not be sought without necessity. No general rule exists for the number of parameters a decompression model should have. The number is a compromise between goodness of fit and the risk of overfitting, and it is better determined by an explicit criterion, such as the Akaike information criterion, than by judgment (Akaike, 2003).

Bühlmann’s model, like any other Haldanian model, falls into the descriptive model category. The ZHL16C algorithm uses 16 compartment periods and 16 M values, each requiring two coefficients, that is, for a total of 48° of freedom (Bühlmann, 2013). It took Bühlmann a lifetime to adjust these parameters for his various models (ZHLA, ZHLB, and ZHLC).

Dive computers essentially use Bühlmann’s models. They compute ascent in real time, but the computation remains, in current instruments, a pressure–time calculation for dissolved gas state. When additional inputs are used, they are typically the measured inspired PO2 in a rebreather, and they update the inert gas fraction rather than alter the model (Schirato et al., 2020; Schirato et al., 2022; Schirato et al., 2024).

We admit, however, that the Haldanian algorithms are reasonably efficient:

- Among the 35 DCS cases recorded after the 33,809 air diving exposures stored in the Comex database, only 3 were considered type II (neurological symptoms). The rest of them were simple type I (pain-only symptoms) (Imbert, 1991). This represents an overall DCS incidence of 1/1000 among commercial divers.
- Among recreational divers, the DCS incidence is even less, around 1 per 10,000 dives, but the exposures differ in depth and time (Cialoni et al., 2017). Live-aboard and dayboat trips include intensive repetitive and multiday diving. A survey conducted over three groups of recreational divers (basic live-aboard, Cozumel dive guides, and Scapa Flow wreck divers) showed that the observed DCS incidence ranged from 0.7/10^4^ for basic diving to 17/10^4^ for wreck diving (Dunford et al., 2020).

Explanatory models not only describe the data but also predict future observations. For this reason, they are more valuable than descriptive models, but they require structuring knowledge through endpoints.

### 2.3 Model endpoints

Models, in general, and decompression theories, in particular, are characterized by the critical endpoints they identify. Haldanian models, for example, aim to control tissue supersaturation to control bubble production and, by extension, control the risk of DCS.

Hills further clarified the issue by differentiating between a “primary event” (decompression, gas phase formation, etc.) and a “critical insult”, which he described as either blood flow occlusion or mechanical tissue distortion caused by decompression bubbles (**Figure 1**) (Hills, 1979). He emphasized that the delay between the primary event and the critical insult, which corresponds to the complex physiological and biological process of symptom development, constitutes the main difficulty in modeling decompression safety (Hills, 1977).

**Figure 1:**
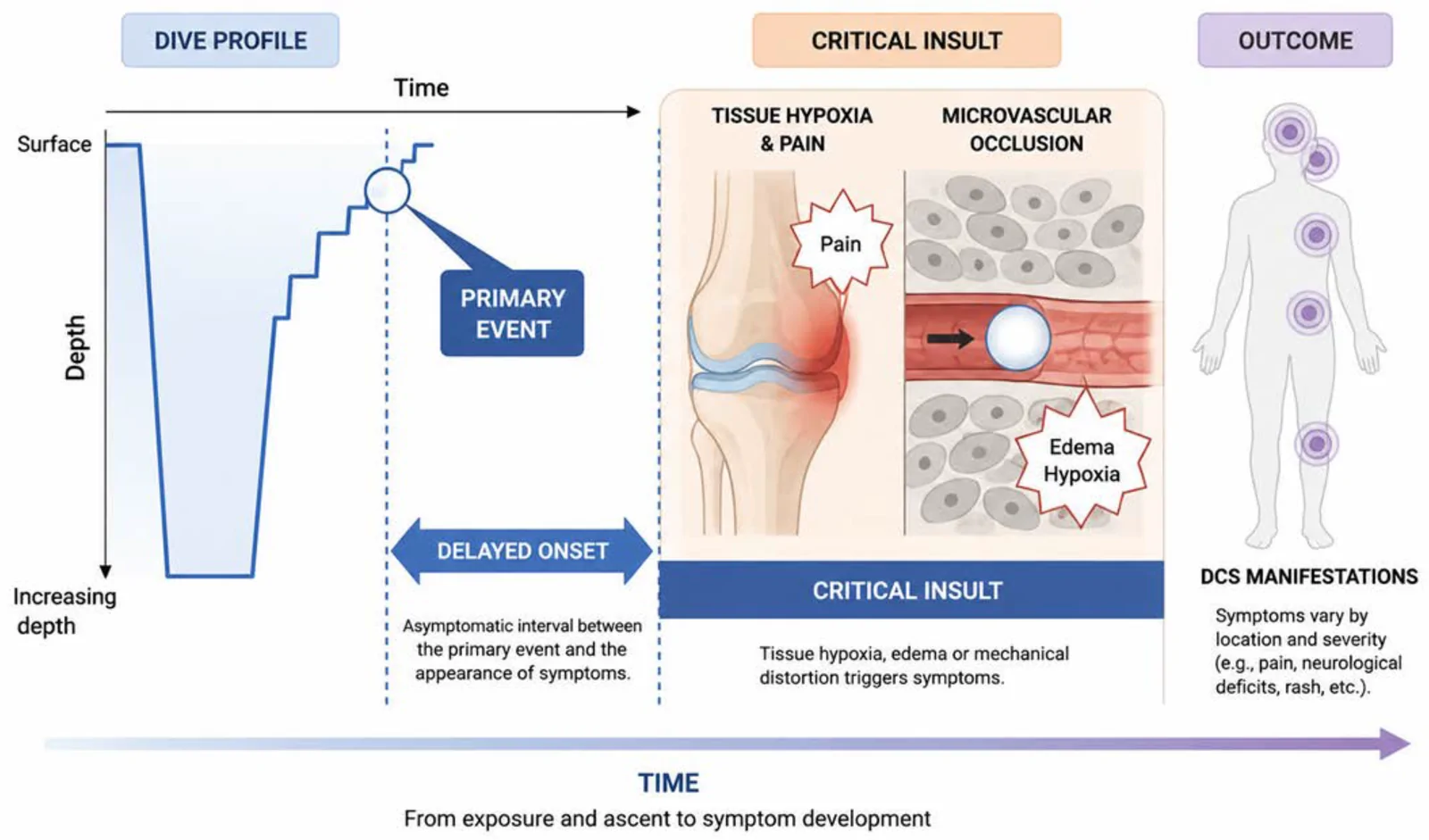
Hills’s definition of the primary event, the onset delay, and the critical insult. The insult was defined through two possible consequences: a vascular bubble occluding a blood vessel and causing tissue hypoxia, or a tissue bubble mechanically distorting surrounding tissue and causing pain, as in the connective tissue of a joint.

Haldanian algorithms track dissolved gas rather than events after phase separation; repetitive or multiday diving and decompression fatigue are therefore represented only through residual gas load. This is a limitation of scope. Adding physiological terms may broaden scope but also introduces additional parameters, often assigned rather than measured.

Haldanian algorithms assume that the primary event and the critical insult coincide. This confines the decompression problem to the moment the safe ascent criterion is violated and excludes the mechanism by which DCS develops. As a consequence:

- DCS is treated as a whole. The symptom type and location are not defined because it is not possible to assign a reliable physiological identity to any compartment.
- The risk of DCS is linked to a threshold with a graded severity proportional to the extent to which the M value is exceeded.
- The time it takes to develop symptoms is not taken into account.

### 2.4 Implication: the decompression stress

In 2005, Brubakk studied the endothelial function after dives and observed a reduced availability of NO associated with impaired endothelial function (Brubakk et al., 2005). He coined the term “decompression stress” to encompass all the adverse effects of decompression (Brubakk and Mollerlokken, 2009):

- Bubbles are the primary physical event. Vascular bubbles may impair perfusion, occlude small arteries, damage endothelium through shear stress, and trigger inflammation; extravascular bubbles may distort tissue, cause joint pain, or reduce spinal-cord perfusion. Consequences include reduced blood supply, endothelial dysfunction, blood–brain barrier damage, and potentially osteonecrosis.
- Inflammation is the second dimension. It may arise from dive-related oxidative stress or decompression-induced platelet/neutrophil activation and MP release. The resulting systemic inflammation can further impair endothelial function, alter susceptibility to bubble scenarios, and accumulate during multiday diving.
- A combination of the above, as bubbles can create MP, and MP can create a second generation of micronuclei.

Following Goldman et al., we have structured the decompression stress along two dimensions (Goldman et al., 2017): one for the bubble scenarios (physical dimension) and another for the inflammation (physiological dimension). Bubbles remain the primary event, and the inflammation is the consequence. Their combination results in decompression stress. Inflammation alone cannot cause DCS (**Figure 2**).

**Figure 2:**
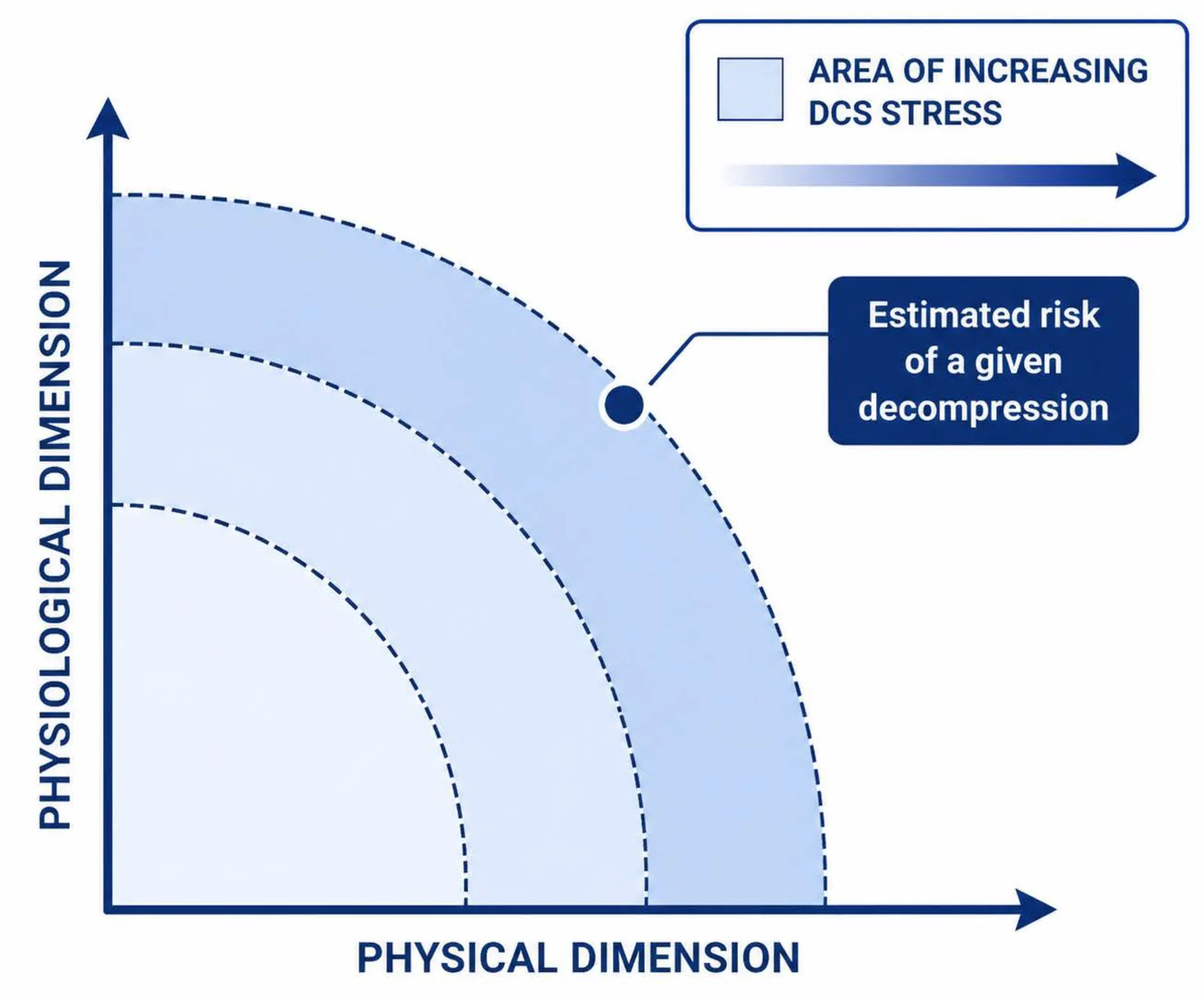
The two dimensions of the proposed decompression stress. Bubbles are the primary event, and inflammation is their physiological consequence. The two events combine to produce a general stress associated with DCS risk.

Physical and physiological dimensions organize decompression stress, but neither has a common quantitative scale: physical stress relies on surrogate endpoints, and no accepted inflammatory-stress metric exists. Surface intervals, days off, and consecutive-diving limits therefore remain empirical; a combined stress measure remains an objective.

## 3 DCS manifestations

In operational diving, DCS is a risk, and decompression procedures are mitigation procedures to control this risk. Hence, DCS is considered the endpoint of decompression models and is classified by its manifestations.

The diagnosis of DCS is based on the classification of its clinical manifestations. Several classification systems of DCS signs and symptoms exist (Desola, 1996), but the most commonly used is the Type I/Type II classification of the US Navy Diving Manual (NAVSEA, 2016). Such classifications serve an operational purpose and allow entry into a flowchart to select a treatment table based on the circumstances of the dive.

### 3.1 Etiology of DCS manifestations

There is a vast scientific literature on the etiology, pathophysiology, and symptomatology of DCS/DCI (Hallenbeck et al., 1975; Hills, 1977; Vann, 1982; Francis, 1998). In this review, we refer to Mitchell’s recent and exhaustive publication in **Table 1** (Mitchell, 2024). It permits sorting the clinical manifestations according to five organ systems (Mitchell et al., 2022):

**Table 1:** DCI manifestations as classified by Mitchell et al., 2022.

Affected organ/system	Manifestations	Description
Musculo skeletal	Pain	Deep, boring ache in shoulder, elbow, hip, knee area, unaffected by joint movement, usually without local tenderness, may be multifocal and poorly located
Skin	Rash and itch	Erythematous poorly demarcated patch, often itchy, or reticular rash with cyanotic discoloration, usually a truncal or proximal distribution
	Patchy paraesthesia	Non-dermatomal distribution, often described as tingling
Inner ear	Vestibular: vertigo, ataxia	Usually accompanied by Nausea and nystagmus.
	Cochlear: hearing loss, tinnitus	Cochlear manifestations are less common than vestibular manifestations
Brain cerebral	Cognitive impairment, scotomata, ataxia, focal weakness, Visual field changes	Typically, mild executive dysfunction (impaired concentration or memory); gross focal lesion considered to be less common in DCS than in AGE
Spinal cord	Motor weakness	Paraplegia or quadriplegia with upper motor neuron signs. Severity ranges from subtle to dense
	Numbness or dense paraesthesia	Dorsal columns appear to be vulnerable; proprioception may be affected
	Bladder and sphincter dystonia	Bladder becomes insensate; urine retention
	Girdle, chest pain, back pain	Typically, a premonitory symptom that precedes other spinal symptoms
Cardio pulmonary	Dyspnea, cough, chokes, chest pain	Typically associated with provocative events such as rapid ascent or omitted decompression
Cardio vascular	Hemoconcentration, shock, coagulopathy	Rare, typically associated with provocative events such as rapid ascent or omitted decompression
Constitutional	Sympathetic or general reaction	Fatigue, malaise, weakness, anxiety, headache

1. Joint (musculoskeletal),
2. Skin,
3. Inner ear,
4. Brain,
5. Spinal cord.

Other occurrences include cardiopulmonary, cardiovascular, and constitutional manifestations.

### 3.2 Classifications of the DCS manifestations with cluster analysis

Ozyigit et al. were the first to propose DCI classifications after processing the manifestations of 1929 DCI cases stored in the DAN database (Ozyigit et al., 2010) using Ward’s method of clustering (Murtagh and Legendre, 2011). Their results could be sorted into 4 clusters:

1. Pain only.
2. Paraesthesia, numbness, decrease in sensibility.
3. Malaise, muscular weakness, paralysis, difficulty urinating.
4. Fatigue, headache, dizziness, skin rash, confusion, nausea, vertigo, altered consciousness, vision problems, hearing loss, tinnitus, swelling, abnormal sensation.

This classification is in line with the work of Howle et al., who analyzed and scored 190 DCI cases recorded after 3222 air and nitrox dives (Howle et al., 2017). They sorted the manifestations into 6 clusters and differentiated between mild type II and severe type II DCI:

1. Pain.
2. Mild neurological.
3. Serious neurological.
4. Cardiopulmonary.
5. Lymphatic or skin.
6. Constitutional or nonspecific manifestations.

A comparable analysis was reported on 605 DCI records from the Comex database using the same method (Imbert et al., 2014). The classification identified four clusters that match the classification of the Comex Medical book:

1. Pain around a joint.
2. Loss of balance, vertigo, nausea, vomiting, vision problems, personality change, fatigue, speech problems, and headache.
3. Paraesthesia, back pain, shock, loss of sensitivity, difficulty standing up, paralysis, bladder problems.
4. Chokes, labored ventilation, cyanosis.

We found a good correlation between scientific DCS classifications and computer classification results. Excluding the last cluster, which represents cardiopulmonary DCS, we drew the pattern trees and identified three clusters emerging from a background of constitutional symptoms (fatigue, headache, etc.) (**Figure 3**).

**Figure 3:**
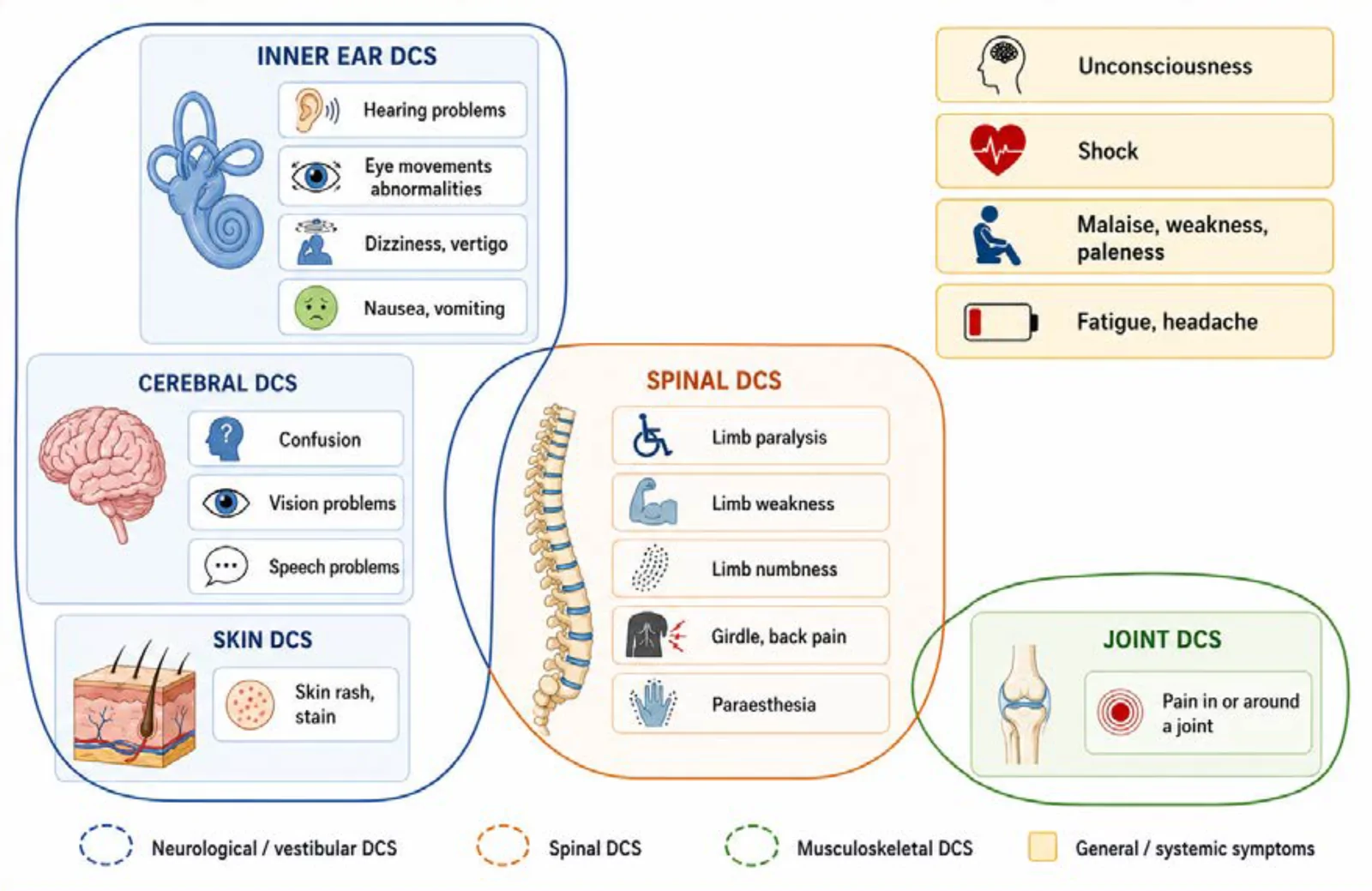
An illustration of the classification obtained with the Ward’s method of cluster analysis showing three overlapping clusters, along with their associated manifestations.

## 4 Bubble physical model

### 4.1 Concept: the three orders of magnitude

When a gas phase can be described with an inside and an outside, it defines a surface with internal and external pressures. According to Gibbs Free Energy, the system tends to minimize its interfacial energy.

At very small scales, classical laws remain relevant, but continuum descriptions become uncertain. When interface thickness is not negligible relative to gas-phase size, macroscopic surface tension, radius, and Laplace pressure lose their usual meaning. We use about 20 nm as a practical transition: Young–Laplace descriptions above it and non-extensive thermodynamics below.

Hence, the evolution of a gas phase activated by decompression covers three orders of magnitude:

- Nanobubbles, identified as precursors to gas nuclei and decompression bubbles (Imbert et al., 2019).
- Microbubbles, which are activated by decompression. They have a size of around 1 micron when they detach from the blood vessel wall (Arya et al., 2023; Bhat et al., 2025).
- Larger bubbles, such as arterial bubbles or VGE, which travel through the blood circulation and can cause DCS through various scenarios. They can be detected by ultrasound Doppler or echography imaging. Doppler devices are preferred for rugged monitoring conditions, with a detection threshold of 10–30 microns (Papadopoulou et al., 2013). Special technology, such as dual-frequency systems, allows for lowering the VGE detection threshold to 1–10 microns (Le et al., 2021).

### 4.2 Concept: bubble shape and the spherical approximation

Gas phases are rarely spherical when constrained. A surface-bound micronucleus is a sessile cap governed by contact angle and adhesion; a vessel-filling bubble becomes an elongated slug; and an extravascular bubble conforms to surrounding tissue. These geometries alter exchange area, diffusion, transit, and mechanical effects.

Because deformation changes exchange and invalidates a single-radius Laplace term, the spherical approximation is only a tractable simplification. Radii should therefore be interpreted as order-of-magnitude volume descriptors, with bubbles depicted schematically.

### 4.3 Concept: gas nuclei

The diving literature refers to gas nuclei as precursors to microbubbles generated during decompression since Harvey’s early work (Harvey et al., 1944). The term is derived from the thermodynamics of cavitation, and it is difficult to draw a transition line between a nanobubble and a nucleus at its start (Caupin and Herbert, 2006).

Gas nuclei are considered the “seed” of decompression microbubbles. Their physical parameters are unspecified, but their size is considered sufficient to obey the classical laws of physics. We therefore propose a terminology based on the evolution of the gas phase from nanobubbles to gas nuclei, microbubbles, and bubbles (**Figure 4**).

**Figure 4:**
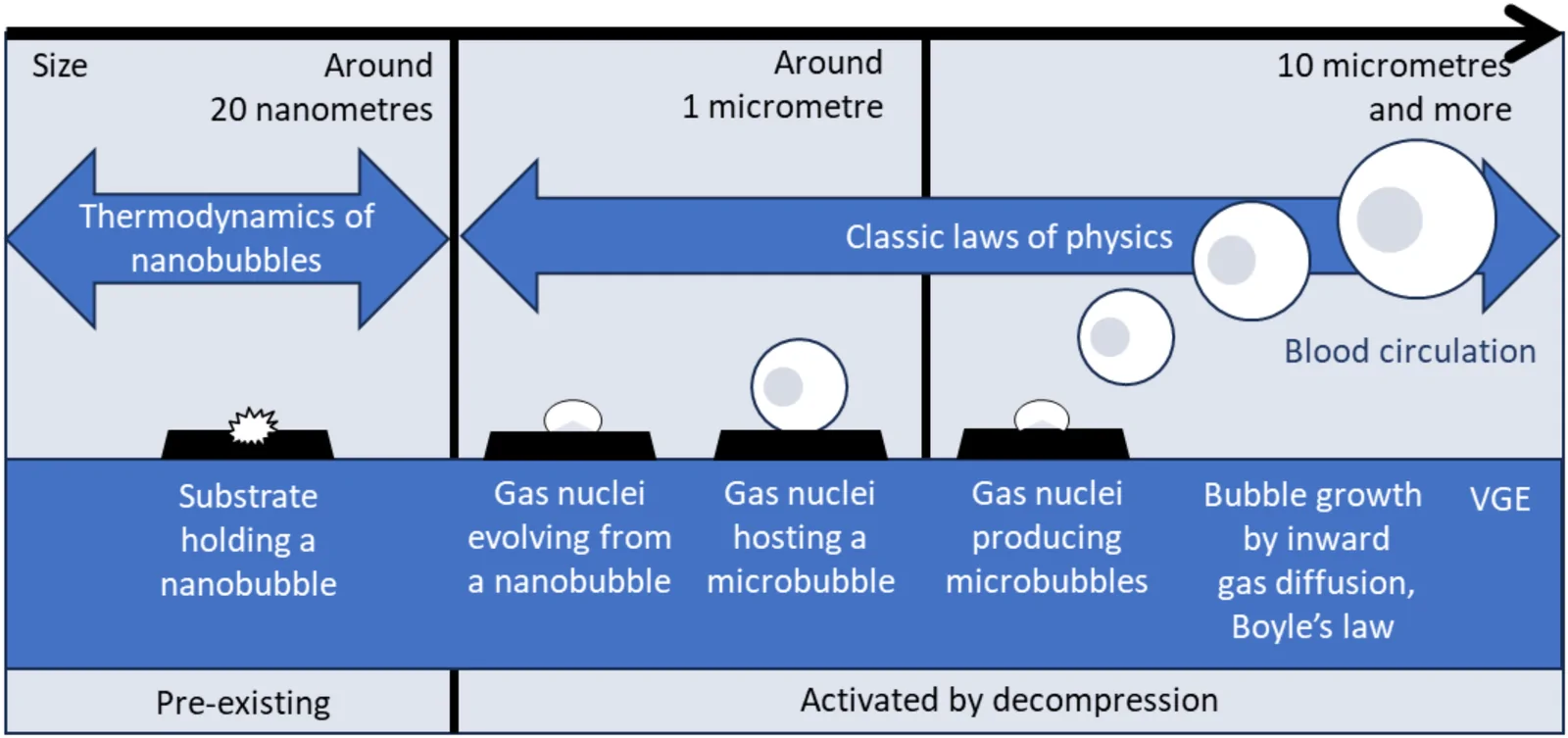
Proposed terminology based on the evolution of a nanobubble to VGE.

### 4.4 Concept: specific sites for gas nuclei formation

The concept is that gas micronuclei form preferentially at specific sites. Other nucleation routes are treated as unresolved rather than excluded.

Silicon-wafer experiments show decompression-induced gas formation on hydrophobic but not hydrophilic surfaces. *Ex-vivo* ovine vessels show reproducible DPPC-positive bubble-forming sites with repeated detachment, supporting vascular plausibility. These findings neither prove the same route in living divers nor exclude alternatives; DPPC co-localization does not establish causality. The complete sequence from pre-existing micronucleus to embolization has not been observed end-to-end and remains Level 2 evidence.

When a hydrophobic surface is submerged in pure water, nanobubbles are formed from dissolved gas. In the first series of experiments, Arieli exposed hydrophobic and hydrophilic silicon wafers to different pressures (Arieli and Marmur, 2013). After decompression, bubbles appeared on the hydrophobic but not the hydrophilic wafers (**Figure 5A**).

**Figure 5:**
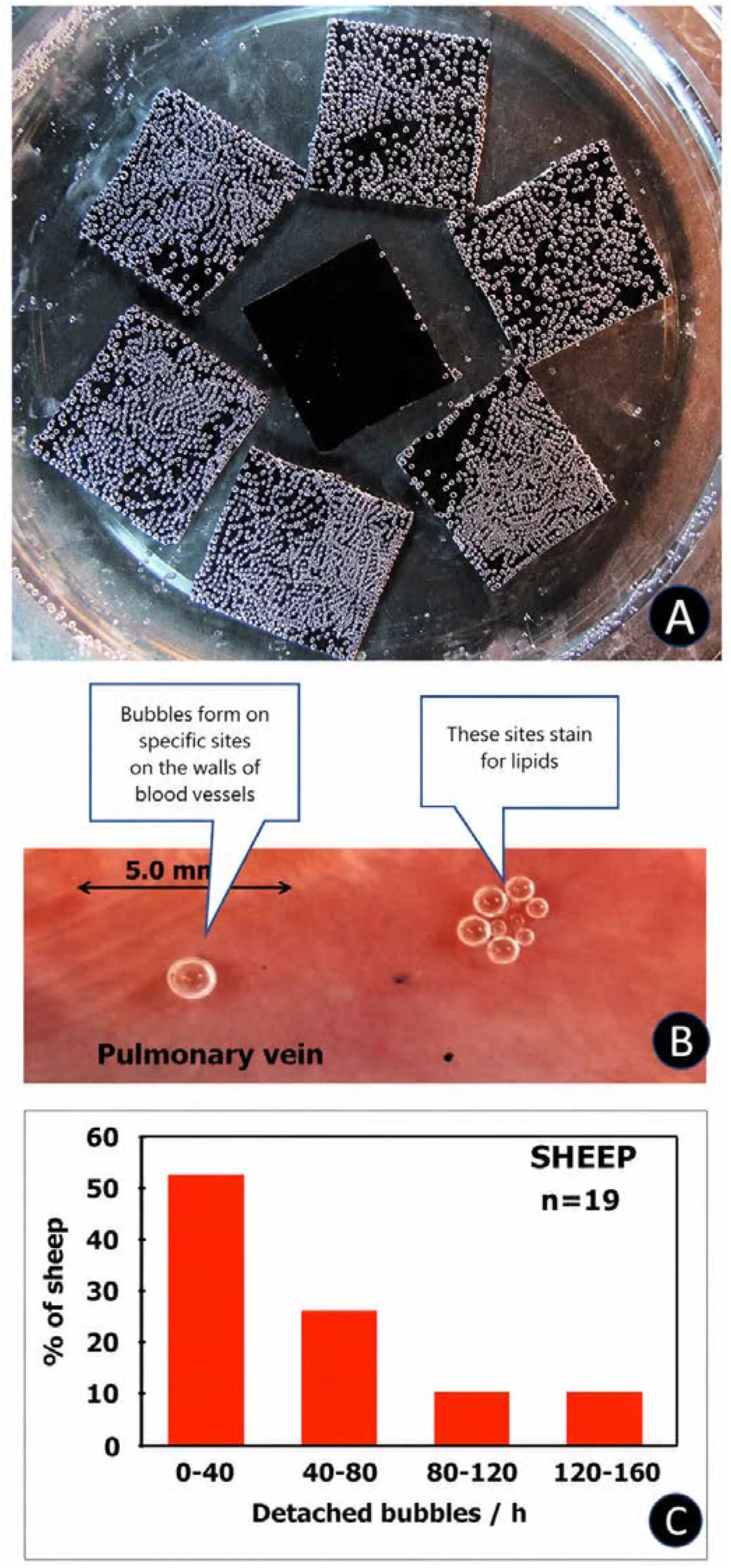
**(A)** Arieli’s experiment with hydrophobic (6 in the periphery) and hydrophilic (one in the center) silicon wafers. After decompression, bubbles only appear on the surface of hydrophobic wafers (Courtesy of Dr. Arieli). **(B)** Photography of bubbles appearing on the wall of the pulmonary vein (with permission from the reference (Arieli, 2019c)). **(C)** Variability of bubble production, expressed as number of bubbles detached per hour, observed from the various AHS in sheep experiments (Reproduced with permission).

The same specificity applies to living tissues. Hills found hydrophobic surfactants by measuring contact angles on various endothelial surfaces from sheep and humans. He proposed that these surface-active phospholipids migrate from lung tissues into the pulmonary circulation and reach downstream intravascular sites (Hills, 1992a). He suggested that pre-existing nanobubbles form from dissolved gas at specific hydrophobic surfaces, identified as the oligolamellar lining, and then migrate into the vascular lumen in certain organs. He showed that phospholipids responsible for hydrophobicity can be found in several tissues, such as synovial fluid (Hills and Butler, 1984), joint surface (Hills, 1989), and nerve myelin (Hills, 1992b). He then postulated that these lamellar bodies are the preferred sites for extravascular bubble formation, in particular, in the spinal cord DCS (Hills, 1992b).

Recently, Arieli conducted *ex-vivo* experiments on large ovine blood vessels, which were placed in a cooled high-pressure chamber at 1,000 kPa for about 20h. Upon decompression, he observed that bubbles formed at discrete sites in all types of blood vessels. These bubble-producing spots stained positive for lipids and were termed “active hydrophobic spots” or AHS (Arieli, 2019a) (**Figure 5B**).

Arieli then sampled the AHS and plasma from the sheep and confirmed that they contained a lung surfactant component: DPPC (Arieli et al., 2016). He concluded that these DPPC deposits constitute the AHS and are the sites of vascular bubble formation (Arieli, 2017). The quantity of DPPC deposits in a tissue indicates the number of potential AHS. The development of DPPC deposits over time indicates the diver’s capacity to form bubbles and, to some extent, their susceptibility to DCS.

### 4.5 Assumption: pre-existing gas micronuclei

It has long been suspected that gas nuclei, responsible for the VGE detected during decompression, were already present in the diver before the dive (Blatteau et al., 2006). Although the current technology does not allow the detection of these nuclei, their existence has been demonstrated by indirect experiments in shrimp (Evans and Walder, 1969). Using the rat model, Vann tried to crush these pre-existing gas nuclei by a rapid compression to 300 msw at the beginning of the dive (Vann et al., 1980).

Many authors have attempted to model gas nuclei as a potential source of VGE formation (Van Liew and Raychaudhuri, 1997). They have imagined gas nuclei stabilized by a skin of material at the liquid/gas interface (Yount et al., 1977), a diffusion barrier (Yount, 1979), or tissue elasticity (Goldman, 2010). They have also proposed local geometry, like hydrophobic crevices, that could stabilize bubbles by surface tension (Harvey et al., 1944; Chappell and Payne, 2006). Finally, they have described how their population could fluctuate and regenerate (Yount, 1982).

### 4.6 Concept: the oxygen window as the factor stabilizing gas nuclei

Gas nuclei, like any gas phase, require an inside–outside pressure difference to be stabilized. Van Liew explained that the inherent tissue unsaturation, or OW, maintains a slight pressure difference between the total tissue gas tensions and the ambient pressure, which can stabilize bubbles (Van Liew et al., 1993).

This can be demonstrated for a spherical air bubble equilibrated with venous gases. The bubble pressure equilibrium for a spherical bubble equilibrated in venous blood is presented below (the v subscript denotes venous) (Equation 1):

(1) $$P_{N2,V}+P_{O2,V}+P_{CO2,V}+P_{w}=P_{amb}+\frac{2\text{γ}}{r}$$

Using the definition of the extended OW provided by Kot et al. (2015) (Equation 2):

(2) $$OW=Pamb-\left(P_{N2,V}+P_{O2,V}+P_{CO2,V}+P_{w}\right)$$

Combining Equations 1 and 2 yield to Equation 3:

(3) $$OW=\;\frac{2\text{γ}}{r}$$

This means that when the bubble is in equilibrium with surrounding gases, venous or tissular, the OW determines the radius of gas bubbles.

We postulate that the OW can be active at the nanoscale. To describe the meta-equilibrium at the level of the nanobubble, we used Non-Extensive Thermodynamics (Letellier and Turmine, 2019) and developed the concept of static metabolic nanobubbles as precursors to gas nuclei (Imbert et al., 2019).

This OW is approximately 7 kPa at standard conditions and falls outside the expected range for a nanobubble’s surface tension. This implies that the nanobubbles are also stabilized by other factors, such as a local geometry (crevices, tubes, …) and a local chemistry (hydrophobic site, large protein cavities, vesicles) to survive this range of undersaturation (Arieli, 2018a; Arieli, 2021a; Arieli, 2021b).

This concept suggests that being alive and maintaining a metabolism could be sufficient to generate and maintain nanobubbles, which would evolve into gas nuclei when a decompression is initiated (Behnke, 1967; Hills and LeMessurier, 1969).

In this scheme, metabolism controls the initial population of nanobubbles. On the one hand, a high metabolism, with high oxygen consumption, lowers the partial pressure of oxygen and increases the partial pressure of CO_2_ in the tissue, resulting in a higher OW (Van Liew and Burkard, 1993). This suggests that exercise and fitness may affect the nanobubble population by increasing their inner–outer pressure difference, thereby enhancing their stability. On the other hand, lower metabolism (aging, rest) would reduce this difference and increase the nanobubble capacity to be activated into gas nuclei by decompression.

### 4.7 Concept: initial gas nuclei population

We combined Hills’ tissular oligolamellar bodies and Arieli’s vascular hydrophobic sites into a general vision of AHS, located either in the tissue or in the lumen of blood vessels. This forms a body of assumptions, figuring the diver, before the dive, as a “bag of bubbles”:

- Nanobubbles pre-exist and are located on specific AHS on the walls of blood vessels or in the tissues’ oligolamellar bodies.
- The OW defines the activation threshold of the pre-existing nanobubbles into gas nuclei when subjected to decompression.
- The initial AHS density defines the number of available sites for gas nuclei and therefore their number. This density varies across tissues depending on the extent of DPPC deposition.
- Vascular gas nuclei can be dislodged from their native AHS by mechanical vibrations, vasoconstriction, etc., and dumped into the blood circulation. At a given time, not all the AHS host nanobubbles.
- The OW can eventually regenerate nanobubbles and therefore gas nuclei.

The theoretical consequence is that the density of available AHS and the initial activation threshold of nuclei located on these sites at the beginning of the dive influence the number of available gas nuclei. The combined AHS and nanobubbles/gas nuclei concepts:

- Give a different role to the tissue and the vascular AHS.
- Provide a way to characterize individual variability in the production of venous bubbles.
- Allow for changing this venous bubble production by pre-conditioning the diver before the dive.

### 4.8 Implication: location of the AHS

Two locations can be considered for AHS (**Figure 6A**).

**Figure 6:**
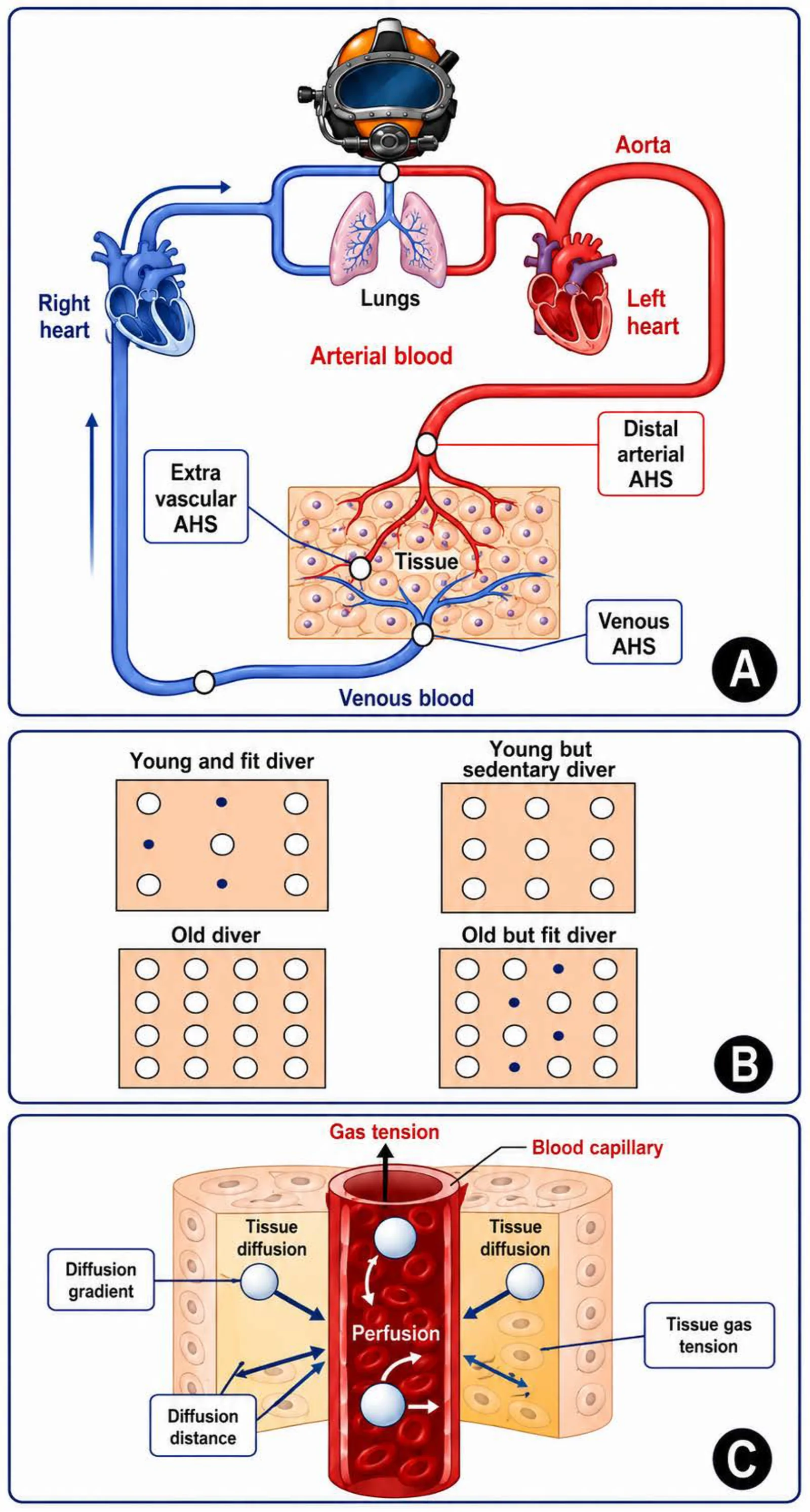
**(A)** Paths to the microbubbles produced at venous and distal arterial AHS. **(B)** Schematic characterization of the diver’s initial gas nuclei population. **(C)** Tissue gas exchanges combine perfusion and diffusion which in turn influence the bubble gas exchanges.

- An extravascular AHS supports a gas nucleus that remains static. Its role is purely local.
- A vascular AHS has a long-range action because it can generate bubbles that travel through the body with the blood flow.

DPPC can be assumed to make deposits anywhere on the blood vessel endothelium, and AHS can form on either the venous or arterial side (Hills, 1992a). Considering decompression, a further distinction must be made depending on whether the vascular AHS is located upstream or downstream of the target tissue:

- A microbubble produced at the level of a venous AHS is exposed to a high inert gas gradient. It is likely to grow and detach during decompression. Once a microbubble becomes bloodborne, it follows the venous blood, passing through progressively larger vessel diameters until it reaches the lung capillaries. A microbubble produced at the level of venous AHS does not become a problem for the diver unless it travels to the lung and passes to the arterial side (Arieli and Marmur, 2017).
- A microbubble produced at the level of a distal arterial AHS is very stable because the local gas gradient is low. However, if it grows and detaches, it would immediately threaten the downstream organ.

### 4.9 Proposed model: individual bubbling variability

The number of VGE detected during a given decompression has long been recognized as an individual characteristic. Hayden et al. have exposed subjects first to a dive and later to an altitude exposure of 3658 m, and measured inter- and intra-individual variations in the VGE grades using cardiac echography (Hess et al., 2021). Cialoni et al. have classified divers from the DAN database into three categories of “bubblers” (non-bubblers, occasional bubblers, prolonged bubblers) (Cialoni et al., 2017). Similarly, Lambrecht et al. have sorted divers into four categories based on their KISS bubble score after a scuba dive at 400 kPa for 30 min (Lambrechts et al., 2013). Other authors preferred to characterize the divers by their bubbling patterns (Papadopoulou et al., 2018).

Considering that the number of pre-existing gas nuclei influences VGE production during decompression, we developed the following assumptions:

- The number of available AHS influences the number of gas nuclei that can be activated by decompression (AHS density).
- Metabolism influences the OW and the activation threshold of pre-existing nanobubbles. This way, metabolism influences the number of available gas nuclei on the AHS (gas nuclei density).
- The preconditioning of the diver prior to the dive may detach some of the gas nuclei and change their density on the AHS.

Animal experiments have permitted the observation of the variability in AHS density and in AHS bubble production (**Figure 5C**). Bubbling in sheep blood vessels depends on the DPPC content of the AHS. It seems that the variations in the density of AHS in sheep are similar to the variations of bubblers/non-bubblers in divers (Arieli and Marmur, 2017).

We hypothesize that the OW contributes to individual bubbling variability. For a spherical gas phase, Equation 3 predicts equilibrium radius inversely related to inherent unsaturation: reduced metabolic oxygen extraction decreases OW, increases equilibrium radius, and lowers the supersaturation needed for growth. Reduced metabolism could therefore allow more micronuclei to reach the growth threshold. This remains Level 3 because surface-bound nuclei are not free spheres, local OW has not been directly linked to whole-body metabolism, and perfusion may compensate; nevertheless, it provides a testable metabolism–bubble link.

We then hypothesize that the continuous deposition of DPPC on blood vessel walls contributes to this health evolution. As the diver ages, the number of AHS increases. This might explain some diseases that develop with age (Arieli et al., 2021). Regarding decompression, the consequences could be:

- Aging reduces overall metabolism, reduces OW, and thus could lower the activation capacity of the nanobubbles and increase the density of gas nuclei.
- Aging increases the number and size of DPPC deposits. Because DPPC is the main component of AHS, a higher density of AHS is expected to generate more decompression bubbles, as observed in older divers (Carturan et al., 1999).

Combining the two assumptions above suggests that the diver’s bubbling variability could also relate to fitness. Exercise increases metabolism, releases mechanical energy, and produces shear stress. Shocks and vibrations tend to mechanically dislodge the gas nuclei from their substrate and reduce their population:

- The protocol for Doppler bubble detection includes a flexion that triggers a rush of circulating bubbles.
- Whole-body vibration performed before the dive reduces the number of decompression bubbles observed after the dive (Germonpré et al., 2009). This action will depend on the activity, as yoga and trampolines may have different impacts in terms of vibrations.
- Exercise induces vasodilation, which stretches the vessel endothelium and may modify the AHS active sites and their capture of nanobubbles. Increased blood flow could mechanically “sweep” nanobubbles away from their AHS, temporarily reducing the number of active sites (Castagna et al., 2011).
- Exercise generates local heat, which modifies the gases’ local thermodynamic properties. Over time, the immediate effects of heat and mechanical energy fade, and the inherent unsaturation likely regenerates nanobubbles. Consequently, not only the intensity of the physical activity but also its frequency will impact the control of the initial nanobubble population.

Finally, we postulate that the diver’s variability in bubbling could be related to their diet. The OW calculation involves the respiratory quotient, which depends on nutrition. High-fat diets lead to additional fatty deposits and, thus, more AHS (Kaczerska et al., 2013). There is renewed interest in nutrition in commercial diving to improve divers’ performance and, eventually, reduce their oxidative stress; the diver’s diet may also have a measurable effect on VGE levels (Valadao et al., 2014).

All these factors can be combined into the broader concept of lifestyle. A survey of Dutch diving instructors found that 90% of responding instructors suffered from cardiovascular disease with above-normal body mass index (BMI), and almost 60% used prescribed or non-prescribed medication (Komdeur et al., 2021). In a study based on VGE detection, Carturan et al. examined age, weight, maximal oxygen uptake, and body fat percentage, and concluded that maximal oxygen uptake (VO_2_ max) was the most significant factor (Carturan et al., 1999). In a first study on the DAN DSL database, which at the time contained 39,099 dives recorded by 2,629 divers, age was identified as a risk factor (Cialoni et al., 2017). In a second recent study of the DAN DSL database, which contains 127,957 dives from 5,907 divers, age was no longer identified as a risk factor, but BMI was (Marroni et al., 2026). It seems from the above that lifestyle factors outweigh age, and an active, healthy lifestyle could help protect against bubble formation and DCS risk (**Figure 6B**).

Other specific individualities, such as gender, could account for differences observed in bubble peak number and time to peak between divers exposed to the same dive, and this is discussed in Chapter 13.

We summarize the individual characteristics identified above and their effects on the post-dive VGE count for a given dive (**Table 2**).

**Table 2:** The potential role of the initial gas nuclei density and AHS density in understanding individual susceptibility, taking post-dive VGE count as an endpoint.

Individual characteristic	Effects	Consequences	Post-dive VGE count
Exercise	Vibrations, mechanical activation, increased shear stress, vasodilation, and heat	Gas nuclei density **↘** Gas nuclei density **↘**	**↘**
Exercise frequency	Frequent exercise counteracts nanobubble regeneration	Gas nuclei density **↘**	**↘**
Fitness	Increased metabolism Increased OW Lower nanobubble activation threshold	Gas nuclei density **↘**	**↘**
Age	Increased number of AHS Decreased metabolism Lower OW Reduced nanobubble activation threshold	AHS density **↗** Gas nuclei density **↗**	**↗**
Fatty diet	Increased number of AHS	AHS density **↗**	**↗**

Down arrow: decrease, up arrow: increase.

### 4.10 Implication: divers’ pre-conditioning

The concept of an initial gas nuclei population has inspired numerous preconditioning experiments (**Table 3**).

**Table 3:** Role of the initial nanobubbles and AHS density in the understanding of diver pre-conditioning, taking post-dive VGE count as an endpoint.

Pre-dive conditioning	Effects	Consequences	Post-dive VGE count
Exercise Vibration Sauna	Vibration, Vasodilatation Heat	AHS density ↘	↘
Oxygen	Increased OW Increased nanobubble activation threshold	Gas nuclei density ↘	↘
Bed rest	Reduce metabolism Reduced activity	AHS density ↗ Gas nuclei density ↗	↗

Down arrow: decrease.

Up arrow: increase.

Pre-dive exercise (Dujic et al., 2004; Castagna et al., 2011), vibrations (Germonpré et al., 2009), sauna (Pontier et al., 2008), mini trampoline (Lambrechts et al., 2022), oxygen breathing, vibration, and oxygen (Balestra et al., 2016b; Germonpre and Balestra, 2017) reduce the VGE observed after decompression, as explained above.

Pre-dive oxygen breathing was shown to reduce post-dive VGE (Castagna et al., 2009). As expected, pre-oxygen breathing first de-nitrogenizes the tissues and protects the diver by reducing the tissue gas load before decompression. It replaces the resident gas in the gas nuclei with oxygen, which is consumed by the mitochondria (Arieli and Aviner, 2020). According to the equation E 3, pre-dive oxygen breathing also drastically increases the OW, increases the nanobubble activation threshold, and reduces the initial gas nuclei density.

Oxygen breathing and vibrations may counteract their effects. In terms of VGE count, pre-dive vibrations better protect the diver than pre-dive oxygen breathing (Arieli et al., 2011; Balestra et al., 2016b). This could indicate that reducing the number of gas nuclei is more effective than increasing their activation threshold.

Bed rest is an extreme situation that minimizes activity and metabolism. According to our model, the nanobubble population could grow out of control in such a situation. Gennser et al. have shown that bed rest significantly increases the post-dive VGE count (Gennser et al., 2018).

### 4.11 Implication: exercise and decompression

Exercise produces muscle contractions, mechanical vibrations, heat, increased metabolism, and perfusion. We have discussed exercise as a preconditioning factor. Its role at the bottom, during decompression, and after decompression is more complex.

Exercise during the bottom phase is expected to increase the muscle tissue gas load by increased perfusion. Shield showed during his survey of North Sea air diving that supervisors are aware of the risk and select the next longer bottom time available in the table when their divers are exposed to hard work or bad conditions (Shields et al., 1989).

Exercise during decompression may have different consequences. On the one hand, it can dislodge vascular bubbles attached to the vessel walls by mechanical action and increase the level of VGE. Depending on the decompression phase, it could facilitate decompression by more effectively eliminating VGE from the lung. It could be expected that exercise during decompression reduces the post-dive VGE level (Jankowski et al., 2004). Dujic et al. have shown that exercise during a 3-min decompression stop reduces post-dive venous gas bubbles (Dujić et al., 2005).

On the other hand, exercise could reduce the lung filtration capacity because it influences ventilation, increases venous return pressure and flow, and reduces the time available for bubble diffusion in the alveoli. It affects the lung’s capacity to filter VGE. It could push shunted microbubbles into the arterial side (Madden et al., 2015).

All these effects can combine after the end of decompression and contribute to the development of MPs (Madden et al., 2016). Dujic et al. showed that VGE count declines during strenuous exercise after an open sea dive to 30 m (Dujic et al., 2006). However, Madden et al. showed that exercise after scuba diving increases the incidence of AGE (Madden et al., 2013).

Finally, high-intensity eccentric muscle work may cause substantial overstretching and damage, thereby inducing bubble formation during decompression. Gottschalk et al. have shown that exercise-induced muscle damage provokes the release of VGE in response to acute decompression (Gottschalk et al., 2023).

The risk of exercise during decompression must be appreciated. It depends on the exercise intensity and the decompression procedure’s safety margin. Today, in the North Sea, saturation divers are authorized to perform light exercise (stepping, bungies) during decompression: divers report feeling and sleeping better during this long, boring decompression time (Imbert et al., 2024a). 

## 5 From gas nuclei to microbubbles

Decompression activates gas nuclei by reducing ambient pressure and creating dissolved-gas supersaturation. Nuclei grow into microbubbles and may detach. *Ex-vivo* observations indicate that an AHS can release successive bubbles, so it is better viewed as a persistent nucleation site than a one-use source. The following sections therefore separate activation, growth, and detachment.

It remains that in any decompression algorithm, gas exchanges must be modeled to describe the capacity of the surrounding blood or tissue to supply this gas nuclei evolution.

### 5.1 Concept: tissue perfusion gas exchanges

In gas exchanges by perfusion, molecules are carried by the blood to the tissue. Blood perfusion depends on tissue characteristics, local metabolic needs, and physiological status (heat/cold, activity/inactivity, etc.). Additional physiological factors, such as intermittent perfusion, uneven capillary distribution, and tissue heterogeneity, further complicate the issue (Homer and Weathersby, 1986). 

Haldanian models bypass the difficulty rather than solve it, by assigning a set of perfusion half-times to a set of theoretical compartments. The approach is convenient and computationally cheap. It is not a physiological description: the compartments do not correspond to identified tissues, and the half-times are fitted to outcome data rather than measured.

Assuming that the tissue is well stirred (even tension across the tissue dimension) and equilibrated with the venous blood, the rate of tissue tension change, dPtis/dt, is expressed by the classic equation (Equation 4):

(4) $$\frac{dPtis}{dt}=\frac{0.693}{T_{perf}}\left(Pa-Ptis\right)$$

Where: Pa and Ptis are the inert gas tensions in arterial blood and tissue. T_perf_ is the perfusion half-time, which characterizes the rate of gas exchange of the tissue compartments.

### 5.2 Concept: tissue diffusion gas exchanges

In exchanges by diffusion, the medium is fixed, and the molecules travel along the gas gradient of the first Fick’s law (Equation 5):

(5) $$J=\;-D\frac{dC}{dx}$$

Where J is the gas molecule flow, D is the diffusion coefficient, and C is the gas concentration at x. Considering the spatial distribution of C, this yields the second law of Fick by differentiation (Equation 6):

(6) $$\frac{\partial C}{\partial t}=-D\;\frac{\partial^{2}C}{\partial x^{2}}$$

Solving the diffusion equation requires defining a local geometry and boundary conditions. It is heavier to handle than the perfusion gas exchange equations. However, when diffusion reaches a steady state, the concentration profile becomes linear, and the inert gas pressure inside the bubble can be expressed from the surrounding tissue gas tension as Equation 7:

(7) $$\frac{dPb}{dt}=\frac{0.693}{T_{diff}}\left(Ptis-Pb\right)$$

Where *T*_diff_ represents a diffusion half-time. In this situation, the rate of tissue gas exchange by diffusion has the same expression as perfusion gas exchange. This duality in the mathematical expressions of perfusion and diffusion exchange might explain why different options yield similar results.

### 5.3 Concept: combined perfusion and diffusion exchanges

Hennessy studied tissue gas exchanges and concluded that they combine perfusion and diffusion (Hennessy, 1971). Hence, both perfusion and diffusion are expected to play a complex role in the development of a gas phase during decompression. Three options are available depending on the AHS location.

The first option is the classic Haldanian gas model that uses perfusion-limited exchanges and assumes well-stirred compartments (Workman, 1965). It works for the microbubbles on the blood vessel endothelium that evolve rapidly because the gradient between tissue and venous blood can be significant.

The second option is to use diffusion-limited gas exchanges, in which the tissue diffusion gradient controls the time response of bubble growth. Hills promoted the concept of tissue bulk diffusion in his “Thermodynamic Model” (Hills, 1977). It works for tissue bubbles that evolve slowly because the tissue diffusion gradient is reduced.

The third option is to use a combined model to better describe the physiological responses. Many perfusion/diffusion models have been proposed, which increase mathematical complexity but have no obvious advantage (Hennessy, 1974; Di Muro et al., 2020).

### 5.4 Implication: which process limits the rate

Both perfusion and diffusion are always involved; no biological situation excludes either. What varies with location and three situations may occur (**Figure 6C**):

- If the diffusion distance is small, exchange is perfusion-limited.
- If the diffusion distance is significant, exchange is diffusion-limited.
- If the perfusion is low, neither process clearly dominates and both gradients must be represented.

Strauss et al. have theorized the consequences of perfusion and diffusion in the “Gradient/Perfusion Model” that they used to explain the occurrence of pain-only DCS associated with tissue bubbles after long and deep dives (Strauss et al., 2018a).

### 5.5 Implication: gas compartment models versus physiological models

Micronucleus growth requires a model of inert gas tensions in surrounding tissue and vascular beds. These are calculated, not measured, values and computational assumptions about perfusion, solubility, and compartment structure.

Setting a critical endpoint in terms of dissolved gas tensions requires defining a target organ with its perfusion and diffusion characteristics, but not its mass or volume. Compartment models are designed to manage dissolved gas thresholds.

However, evaluating the amount of VGE produced during decompression requires modeling gas quantities and, consequently, defining mass or volume for each tissue. As discussed, the lung transfer function depends on the VGE dose in venous blood return and therefore requires a different gas-exchange model. Flook implemented Mapleson’s model to estimate VGE levels detected after decompression (Mapleson, 1963; Flook, 2011). This simple electrical analog includes the physiological definition of eight tissues. Computing venous return gas tensions is based on mass-balance and, therefore, on blood volume, tissue-blood partition, and tissue characteristics (mass, solubility, and diffusion coefficients) for each tissue considered.

Recently, Théron et al. published a model that includes a broader set of physiological terms than classical compartment models, enabling the integration of physiological parameters (blood and respiratory flows, alveolo-capillary diffusion, respiratory and blood volumes, oxygen consumption rate, etc.) into a model of nitrogen saturation/desaturation, in which oxygen and CO_2_ partial pressures can also be incorporated (Theron et al., 2025). Such a model includes morphological parameters (weight, body composition, fat content of each compartment, VO2 max) and dive situations (heat and cold, hard work, hyperventilation), provided it establishes a link between dissolved gas and a critical decompression condition. Most of its parameters are assigned from population values in the literature. Its interest lies in making the physiological assumptions explicit enough to be examined and, eventually, measured.

## 6 From distal arterial AHS to arterial microbubbles

We postulate that AHS exist on the walls of distal arteries/arterioles and could support a population of gas nuclei.

The arterial tree, which bifurcates into distal arteries and arterioles, supplies blood to the tissue microcirculation. No supersaturation is expected in arterial blood because it is saturated with alveolar gas, and there is no OW. Further downstream, at the level of distal arteries and arterioles, decreasing vessel diameters and slower flow rates enable gas exchanges with the surrounding tissue (**Figure 7A**). During decompression, inert gas gradients will induce inward diffusion, microbubble growth, and detachment. A microbubble produced at the level of a distal arterial AHS is a direct threat to the downstream tissues (Arieli, 2023).

**Figure 7:**
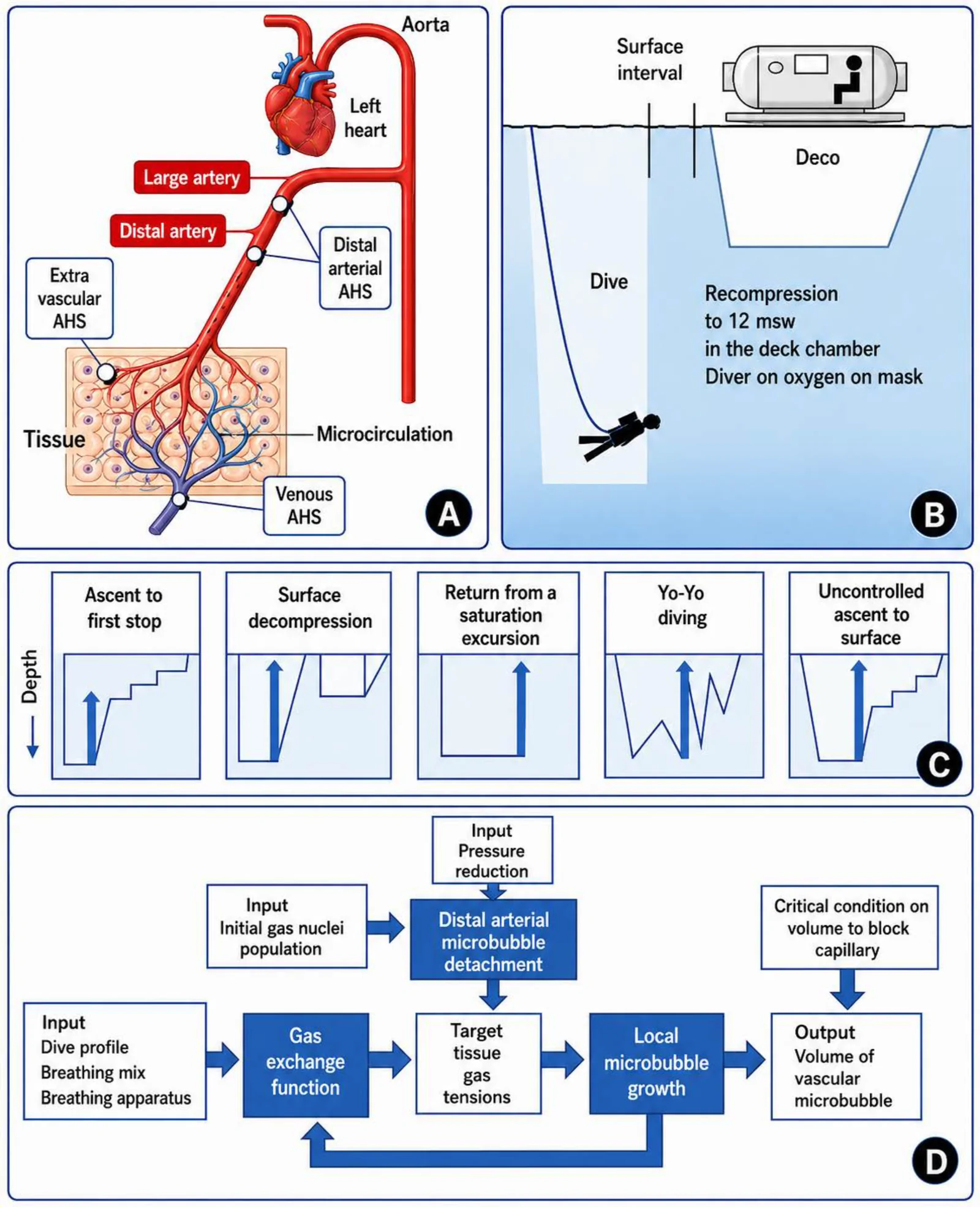
**(A)** The scenario for distal arterial AHS. **(B)** Surface decompression using oxygen. **(C)** Diving situations associated with rapid pressure reductions that potentially activate distal arterial AHS and release arterial microbubbles. **(D)** Description of the distal arterial microbubble growth once stuck at the level of the target tissue.

The scenario of an arterial microbubble formed at the level of a distal artery/arteriole is unlikely to compete with that of a shunted arterial bubble caused by right-to-left shunts. The literature on inner DCS indicates that the large majority of the cases are associated with a right-to-left shunt (Gempp and Louge, 2013; Mitchell and Doolette, 2015). Lung shunts intrapulmonary arterial-venous anastomosis (IPAVA) likely account for the remainder of cases. However, the distal arterial bubble hypothesis provides an alternative that can explain some marginal cases when:

- The time course is too short to allow VGE production and subsequent shunted arterial bubbles, as during the initial ascent to the first stop.
- There is not enough dissolved gas to generate VGE, as in breath-hold diving (Arieli, 2019b).

### 6.1 Concept: activation of arterial AHS by Boyle’s law

Gas nuclei located at a distal arterial AHS are exposed to little or no gas gradient, even during decompression. They are equilibrated with their arterial environment and are likely to be very stable (Arieli, 2023). However, we postulate that a sudden pressure reduction, such as a rapid ascent to the first stop, could expand their volume according to Boyle’s law, thereby causing their detachment.

Several diving situations can expose divers to a rapid reduction in ambient pressure. Some are part of routine dive procedures (ascent to first stop, surface decompression, downward saturation excursions), and some are accidental (yo-yo diving, uncontrolled ascent to the surface, omitted decompression). Dive situations in **Figure 7C** illustrate how dive procedures or profiles can cause arterial embolization. Yo-yo diving (Gempp and Peny, 2016) and upward saturation excursion dives of high amplitudes (Brubakk et al., 1986; Flook, 2004) are known to produce type II DCS.

### 6.2 Implication: rate of ascent to the first stop

Decompression tables begin with an ascent to the first stop, which lasts only 2 to 4 minutes. During this short period, it can be assumed that:

- The diffusion exchanges between the bubble and its surroundings are minimal, and Boyle’s law prevails.
- The pressure reduction causes the AHS gas nuclei to expand and detach microbubbles from their substrate.

A rapid ascent to a shallow first stop could activate distal arterial AHS and generate a flush of arterial microbubbles with direct access to downstream tissues. Carturan et al. have confirmed that a faster rate of ascent to the first stop corresponds to increased production of VGE (Carturan et al., 2000).

### 6.3 Implication: deep stop

Scientific diver Richard Pyle reported feeling less post-dive fatigue after deep rebreather dives during which he collected fish. For these dives, he described having to introduce an additional deep stop to puncture the fish’s bladders before surfacing. He suggested that this additional stop in his deep technical dive was the reason for his lesser post-dive fatigue (Pyle, 1997).

Bennett and co-authors found that deep decompression stops can reduce VGE count and fast tissue gas tension (Marroni et al., 2004; Bennett et al., 2007). However, there has been a long-standing controversy over the benefits of deep stops (Pyle, 2001; Fock, 2007).

One reason could be that deep stops must be associated with deep dives. A study conducted by DAN at 25 msw using VGE count as an endpoint seemed to indicate the benefit of a 15 msw stop for a few minutes, but the experimental protocol was biased by changes in the shallow stops (Bennett et al., 2007). When a similar study was conducted with equal amounts of ascent time in both groups (stops 3 min “deep” + 4 min “shallow” vs. 7 min “shallow”), the result was reversed (Schellart et al., 2008).

Another reason is that deep stops have limits. A deep stop may be detrimental if the additional stop time increases the tissue gas load. The US Navy conducted an extensive study involving 390 men x dives to 51 msw for 30 min bottom time on air, using two decompression profiles: one with a first stop at 21 msw, and the other at 40 msw. They recorded a higher DCS incidence with the deeper stop profile (Doolette et al., 2011).

A last reason could be a scientific protocol bias. The French Navy showed that adding deep stops increased the VGE count in some deep-air experimental dives. The time added to the deep stop was subtracted from the time of the shallow stops, which changed the end of the decompression (Blatteau et al., 2005).

Deep stops can increase inert-gas uptake in undersaturated compartments, which must later be eliminated at shallower depths. The penalty depends on exposure, gas, ascent, and stop pattern; these findings suggest that deep stops provide options for decompression strategies rather than implying that every deeper stop is harmful.

Recently, two groups of technical divers breathing trimix as bottom mix and Nitrox 50 as decompression mix, performed a dive at 40 m for 40 min. The first group started decompression stops at 21 m and the second at 9 m. VGE were significantly reduced after the deep stop profile. This observation suggests that deep stops can also provide a procedural advantage as the divers had the opportunity to switch to N50 earlier in the ascent (Marroni et al., 2026).

### 6.4 Implication: surface decompression

Commercial air diving uses two decompression methods for surface-supplied diving:

- In-water decompression (no-stop decompression or stop decompression).
- Surface decompression using oxygen (SDO2), mainly used when the sea state conditions do not permit in-water stops (**Figure 7B**).

Surface decompression using oxygen begins with a direct ascent to the surface, followed by recompression into a deck chamber. The surface interval is measured from leaving the last in-water stop to reaching chamber depth, and the procedures from which this figure is drawn specify a limit of 3 min. The diver resumes decompression at 12 msw inside the chamber, breathing pure oxygen on mask.

The survey performed by the British authorities over air diving operations in the North Sea has shown that SDO2 was a risk factor for type II DCS (Shields and Lee, 1998).

A similar survey was performed using the Comex database, comparing DCS incidence rates for in-water and TUP air decompressions (continuous ascent) with SDO2 (Imbert, 1991). Based on their Prt index, the exposures were classified into three severity levels. Comparing type I DCS incidence did not differentiate between decompression methods. In both cases, the observed incidence rate increased with exposure severity, indicating a direct relationship between the risk of type I DCS and tissue gas load. The comparison of type II DCS incidences showed that SDO2 had a significantly higher incidence than in-water decompression for severe exposures.

These two studies suggest a specific risk of Type II DCS associated with SDO2 for severe exposures defined as Prt > 35. Given the short time interval between ascent to the surface and recompression to 12 msw, we postulate that this specific risk results from the activation of distal arterial AHS by Boyle’s law.

### 6.5 Proposed model: the distal arterial AHS scenario

We propose translating the above concepts into a module called the arterial microbubble growth function. It combines the effect of sudden pressure reduction, which detaches an arterial microbubble that travels downstream to a target organ. There, the local tissue supersaturation determines whether the microbubble passes by, or stops and grows until it eventually blocks the blood supply (**Figure 7D**).

Occlusion is an intuitive consequence of an arterial microbubble but may not be the most common. Collateral circulation, dissolution during transit, and the small size needed to cross the pulmonary circulation limit complete blockage. Repeated microbubble contact may instead disturb endothelial function, increase permeability, and initiate local inflammation without persistent occlusion.

The function input parameters include:

- The dive profile.
- The breathing mix or diluent mix supplied.
- The breathing apparatus used with its settings.
- The diver’s bubbling “function.”
- And the sudden pressure reduction.

The function adjustment parameters include:

- The parameters required for defining the gas exchanges at the tissue level.
- The parameters of a bubble growth model.

The function output is:

- The volume of the distal arterial microbubble, which is compared to a critical threshold with the capacity to block the distal artery and/or capillary.

## 7 From venous AHS to VGE

Venous AHS and their hosted gas nuclei initiate the second bubble scenario. It corresponds to the journey of vascular microbubbles located on a venous AHS that (Erreur! Source du renvoi introuvable.):

1. Grow until they detach.
2. Travel with the venous blood, where they develop to the size of VGE.
3. Reach the lungs, where they are expected to be trapped by size and eliminated by diffusion into the alveoli.

### 7.1 Concept: site for nanobubbles activation

Once the diver begins their ascent, a gas nucleus on the venous AHS is subjected to an ambient pressure reduction and gas gradients that cause a volume increase and inward diffusion (Papadopoulou et al., 2013). The gas nucleus becomes a microbubble and reaches a critical volume at which it detaches from the AHS substrate by buoyancy, becomes blood-borne, and continues to grow within the venous flow, either by inward gas diffusion or coalescence (**Figure 8A**).

**Figure 8:**
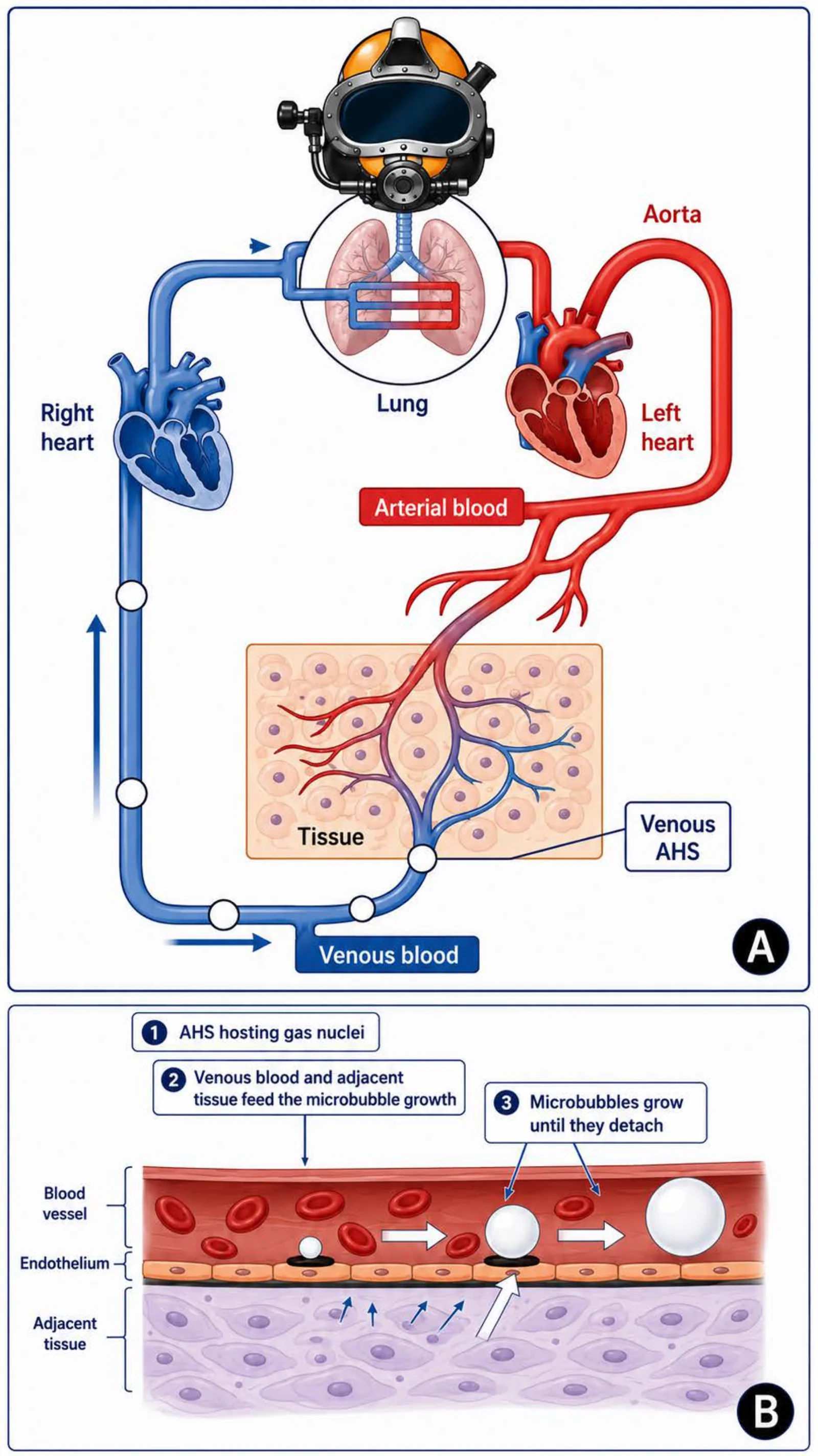
**(A)** Description of the venous AHS scenario. **(B)** Activation of gas nuclei located on a venous AHS supporting the growth and the detachment of microbubbles by decompression.

### 7.2 Concept: delay in microbubble growth

Diffusion requires time and delays VGE production. Microbubble growth depends on the available diffusion area: the larger the size, the greater the surface area, and the faster the expansion. In excised sheep vessels observed optically after decompression, growth appeared to proceed in two phases. The values below come from a single preparation type, in an excised vessel at atmospheric pressure with a fixed orientation, so that buoyancy which drives detachment there does not act as it would in a diver in any posture; the smallest bubbles are at the limit of what the optical method resolves, so the early phase is characterized less reliably than the late one; and the sample sizes are small. They should be read as orders of magnitude (**Figure 9A**) (Arieli et al., 2015):

**Figure 9:**
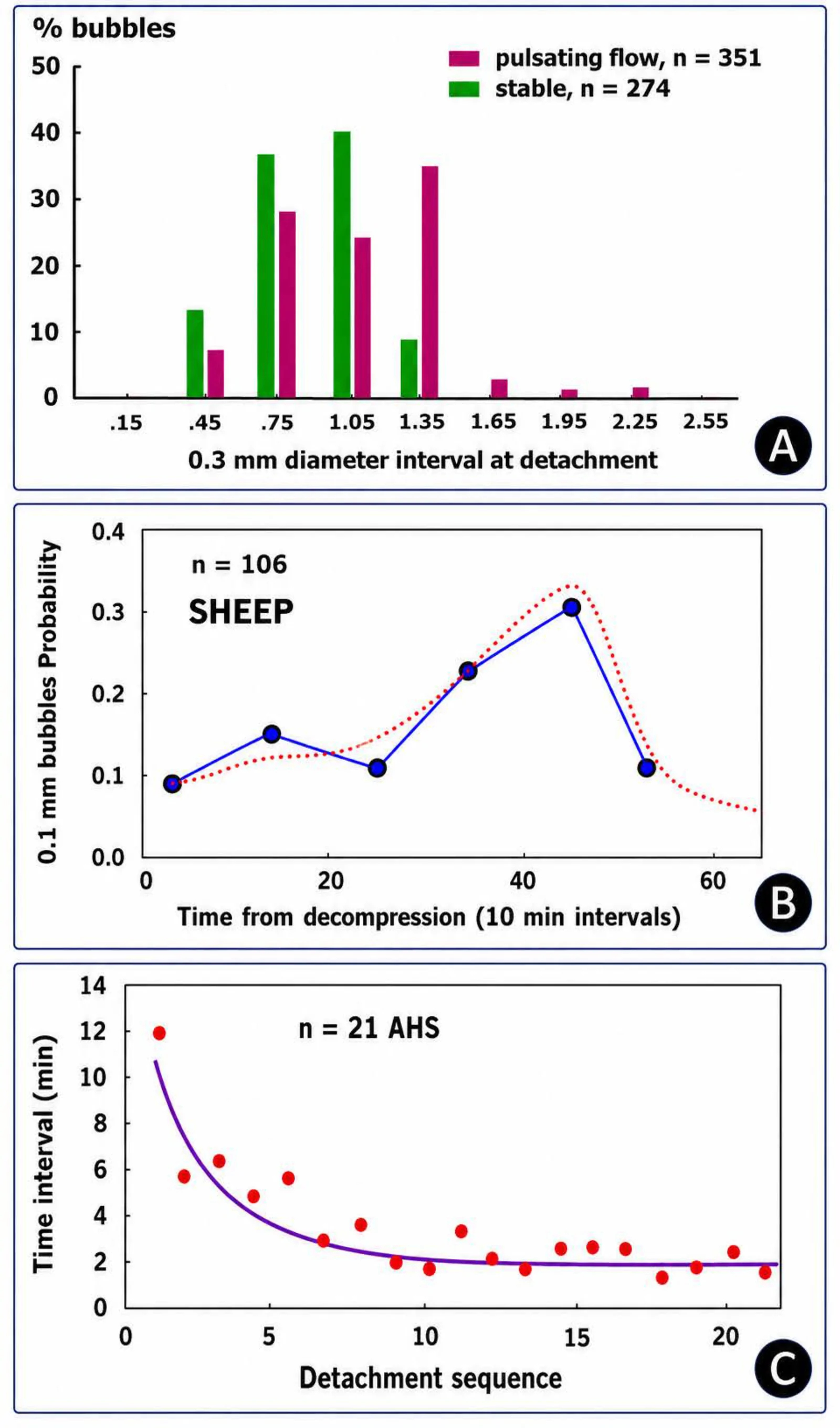
**(A)** Probability for the initial detection of a bubble with a diameter of 0.1 mm versus time, versus the time elapsed after decompression, on a sheep blood vessel [Modified with permission from Arieli (Arieli and Marmur, 2016)]. **(B)** Size distribution of bubbles detached from the blood vessel [Reproduced with permission from Arieli (Arieli et al., 2015)]. **(C)** Time intervals between bubble detachments from the AHS of sheep blood vessels after decompression as a function of detachment order [Reproduced with permission from Arieli (2017)].

- A slow initial expansion until the microbubble reaches a diameter of the order of 0.1 mm, peaking about 45 min after decompression.
- A subsequent faster growth, over about 12 min, to a diameter of the order of 1 mm.

### 7.3 Concept: detachment of microbubbles

The conditions that allow a microbubble to detach from its substrate are critical to subsequent decompression. Detachment occurs when the buoyancy force exceeds the AHS adhesion forces (Arieli et al., 2015; Arieli and Marmur, 2017).

Decompression of large sheep blood vessels has shown that buoyancy causes bubbles to detach from the AHS at a mean diameter of about 1 mm (**Figure 9B**). This compares with the 4.2 mm bubble detachment diameter observed in the previously mentioned hydrophobic silicon wafer experiment. This difference could be due to tearing off the underlying endothelial cells, and the corresponding size in a diver, about 100 µm, is an estimate derived from these observations and not a measurement (Arieli and Marmur, 2014).

### 7.4 Implication: AHS activation by repetitive diving

Experiments on sheep blood vessel tissue have shown that microbubbles developing at the AHS level strongly adhere to their support (**Figure 10**). Microbubbles of 1–2 mm diameter, subjected to a pulsatile flow simulating blood circulation, remained attached to the wall (Arieli et al., 2015).

**Figure 10:**
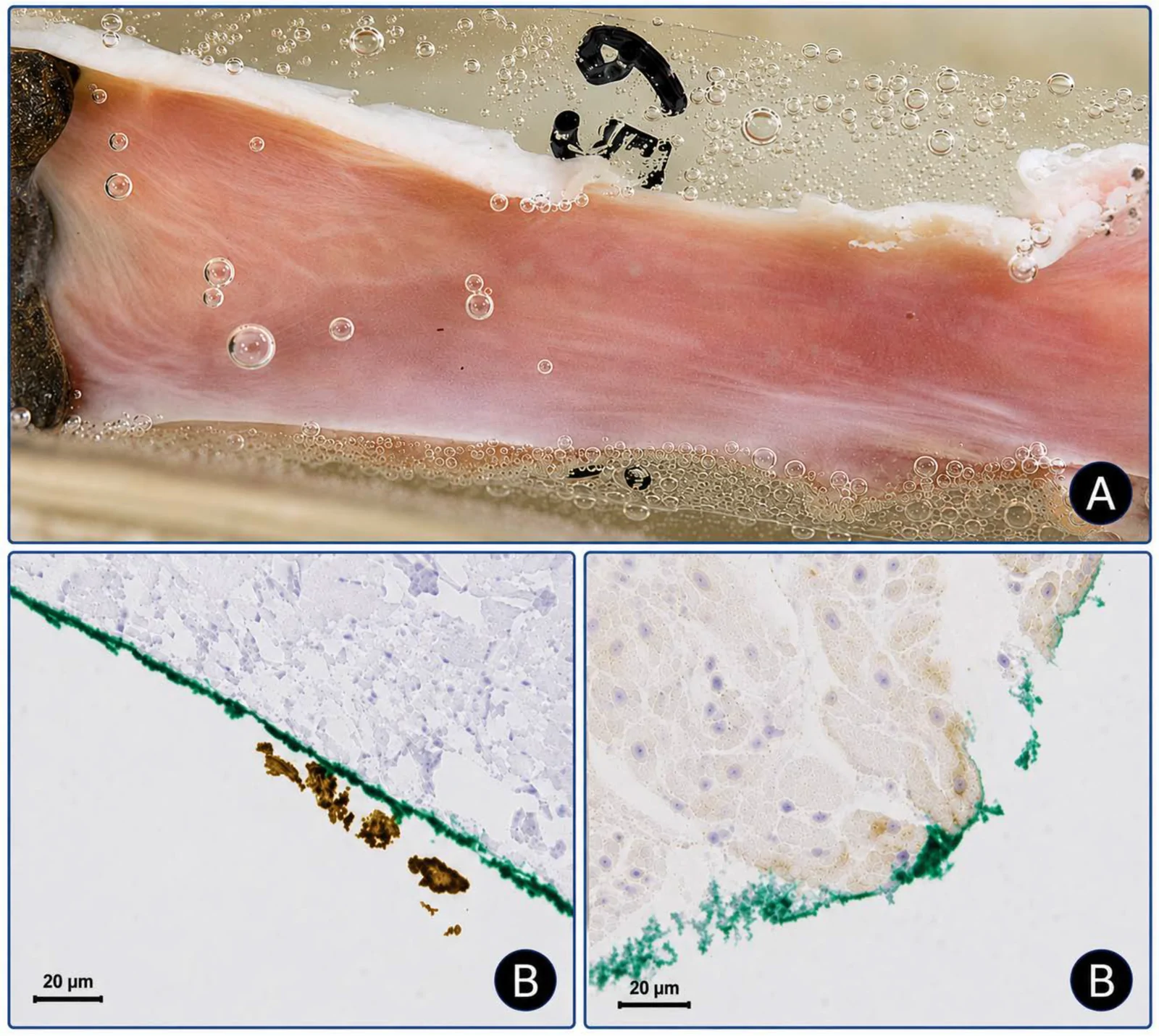
**(A)** Screen copy of a video showing bubbles (2 mm diameter) attached to a sheep vessel wall and exposed to a pulsatile flow. Despite their large dimension, the bubbles firmly adhere to their base support (AHS). **(B)** Light microscopy of the inner part of the blood vessel after decompression. Endothelial cells are stained brown. The left panel was taken at the AHS site, where detached bubbles left debris on the endothelial cells. The right panel was taken at a distant location from the AHS and showed intact endothelial cells. Reproduced with permission from Arieli (2021c).

After the first bubble detachment, a given AHS can produce a continuous flow of microbubbles, and the production rate varies with time (Arieli, 2017). **Figure 9** shows the bubbles produced on sheep blood vessels after decompression. It took 12 min from decompression to the first bubble detachment. This time interval was 4 min between the fourth and fifth detachments and only 2 min between the eighth and subsequent detachments. This indicates that an AHS increases its microbubble production rate until it reaches a steady rate after a certain number of microbubble detachments.

This could explain why more bubbles are produced after a second dive (Dunford et al., 2002). This finding is particularly relevant to repetitive diving, which involves a dive performed with a surface interval shorter than 12h, and is associated with a higher level of detected VGE (Schellart and Sterk, 2012). Short surface intervals are critical in repetitive dives (Imbert et al., 1992).

In recreational diving, repetitive diving is common (two-tank dive packages, night dives), but dive depths generally remain shallow, and bottom times are limited to the no-decompression envelope. During live-aboard diving cruises, the overall DCS incidence is low (Gillian, 1991).

In commercial diving (deeper, longer, and more demanding dives), repetitive diving has long been associated with an increased risk of DCS. As mentioned, the survey of North Sea commercial air diving identified repetitive diving as a risk factor (Shields et al., 1989).

### 7.5 Implication: AHS depletion by continuous diving

Mediterranean red coral divers have developed a progressive approach to increasing depths during their diving season. They start in April with moderate dives at 40 msw, then increase depths as the weather improves. By June, they operate at about 60 msw, reaching 80 msw in July. Only in August and September would they consider diving a specific spot at 90 msw, which they expect to bring an exceptional harvest.

Their progressive exposure to increasing depths has many operational justifications, but can also be considered an empirical approach to managing their AHS stock, developed during the winter when they do not dive. Light microscopy has shown that microbubbles detaching from their substrate damage the endothelium and carry away debris from the AHS substrate (Arieli, 2021c). This suggests that, dive after dive, the AHS are reduced or damaged, and their population is progressively depleted (**Figure 10B**).

Similarly, Zanchi et al. studied repeated daily diving over four consecutive days and reported a reduction in bubble formation across the series, which they themselves discussed in terms of acclimatization to diving (Zanchi et al., 2014).

Risberg later reviewed the effect under the same heading (Risberg, 2021). Our framework offers one candidate mechanism for this reduction—progressive depletion of available AHS—that would not require any biological adaptation. We cannot claim that no biological change occurs: nothing in these studies was designed to detect one, and a response developing over several days falls within the timescale at which adaptive changes in endothelial function, antioxidant capacity or fluid balance would be expected. The two explanations are not exclusive, and the available data do not separate them. On terminology, we use preconditioning for single interventions applied shortly before a dive, where the intended effect is immediate and transient, and retain acclimatization (which may better understood sometimes as depletio of pre-existing micronuclei) for the effect of repeated exposure over days, which is the established usage and does not prejudge the mechanism (Blatteau et al., 2008; Germonpré et al., 2009; Balestra et al., 2016b; Germonpre and Balestra, 2017; Lambrechts et al., 2022).

### 7.6 Implication: VGE distribution in venous return

The venous system collects the vascular bubbles produced by various tissues during decompression. Merging venous flows brings together bubbles of different volumes, depending on the tissue supersaturation history (**Figure 11A**). Coalescence may occur. As a result, the VGE converging to the vena cava presents a large distribution of sizes that can easily be observed during cardiac echography in the right heart cavities (**Figure 11B**).

**Figure 11:**
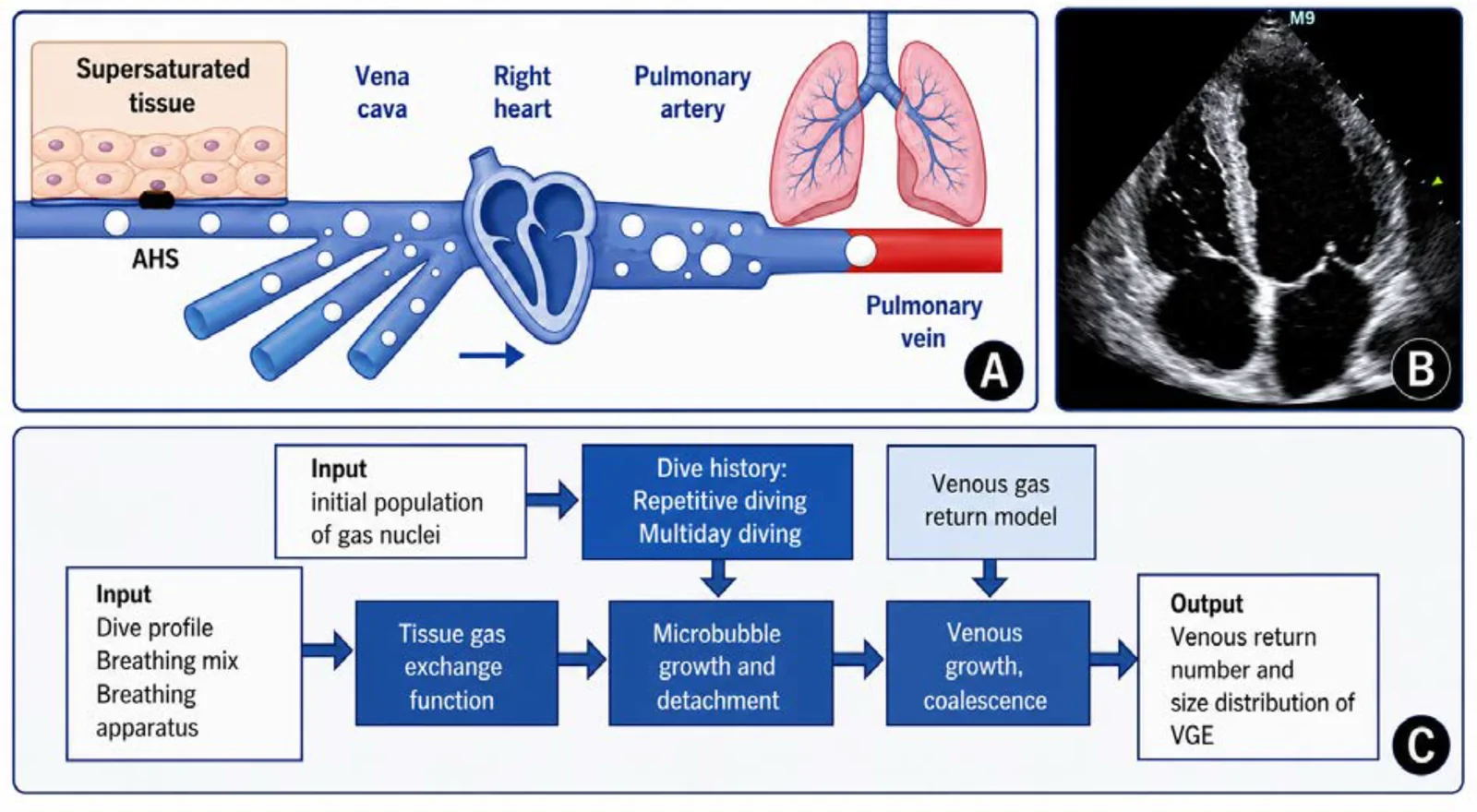
**(A)** Echocardiography of a decompressing diver showing VGE in the right cavities of the heart (the right cavities of the heart are on the left side of the image with the cardiac apex upward). **(B)** Size distribution of VGE in the venous return. **(C)** The VGE dose function.

This dose of incoming VGE to the lung is critical as input to the lung transfer function, which is detailed in the following chapter.

### 7.7 Proposed model: the VGE dose function

We propose to translate the above concepts into a module called the VGE dose function. The function models the various steps of nanobubble activation, growth, detachment, and further evolution in the venous return (**Figure 11C**).

## 8 From VGE to shunted arterial microbubbles

Arterial microbubbles can be traced back to Haldane who stated in his 1908 historical paper:

“If small bubbles are carried through the lung capillaries and pass, for instance, to a slowly desaturating part of the spinal cord, they will there increase in size and may produce serious blockage of the circulation or direct mechanical damage”. (Boycott et al., 1908).

Hills was the first to detect arterial bubbles using the Doppler ultrasound technique in animal experiments (Butler and Hills, 1979). Arterial bubbles were then detected in divers, and their possible role was discussed during the deep Norwegian experimental dives (Vik et al., 1990). The model was then proposed by James et al. for the onset of central neurological symptoms (James, 1982). Finally, Hennessy published in 1989 all the physical aspects in a paper that became the foundation of the arterial bubble scenario (Hennessy, 1989a).

Arterial bubbles became less prominent as measurable VGE became the dominant endpoint. However, right-to-left shunts are strongly associated with several DCS manifestations and provide a plausible route for venous bubbles to arterial target organs. We therefore treat arterial bubbles as one component of a broader testable framework, not an exclusive explanation of DCS.

In this scenario, venous bubbles reach the lungs, are trapped in alveolar capillaries by size, diffuse gas into the alveoli, and dissolve. Hennessy hypothesized that a bubble crossing the lung filter could reach supersaturated tissue, grow, occlude circulation, and cause DCS. Lung bubble filtration is therefore critical to safe decompression.

We identify four situations for the failure of lung bubble filtration:

- Size smaller than the filtration threshold.
- Exceeding the diffusion capacity.
- Shunt in the lung through anastomoses.
- Right-to-left cardiac shunts.

### 8.1 Concept: lungs as a low-pass filter

The lung filter is a low-pass filter with a threshold radius around the size of a blood cell [7.5 to 8.7 μm in diameter and 1.7 to 2.2 μm in thickness (Diez-Silva et al., 2010)].

The threshold has been estimated using an intravascular ultrasound technique with a contrast agent called Levovist (Besnard et al., 2006). The agent contains stable gas bubbles trapped in galactose with a calibrated size distribution of 3 to 8 microns. The contrast agent was injected intravenously, and measurements were taken a few minutes later in the cerebral, renal, and lower-limb arteries. If contrast bubbles up to 8 µ can cross the lungs, it means that tiny decompression bubbles and MP can freely pass to the arterial side (**Figure 12A**).

**Figure 12:**
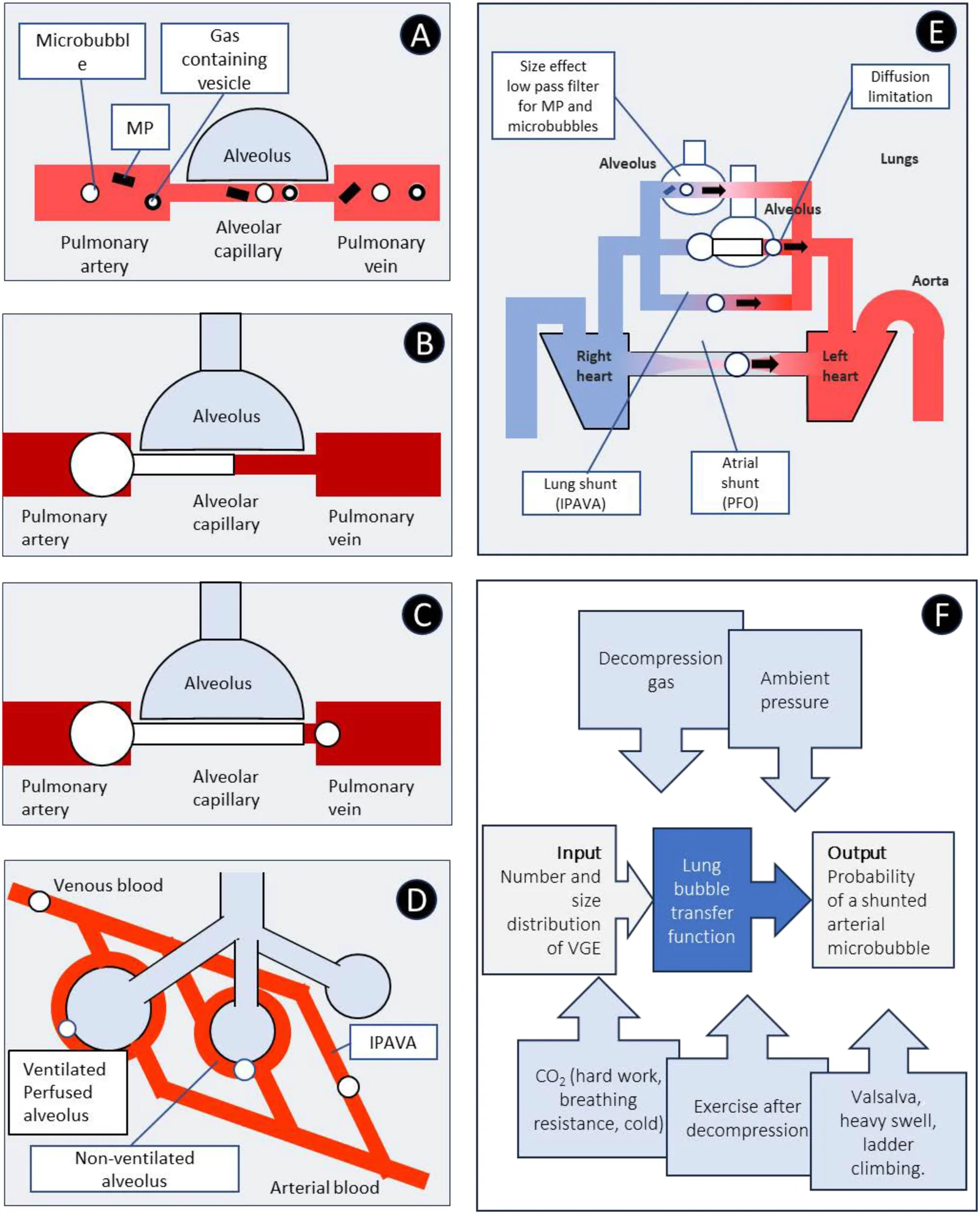
**(A)** The lungs as a low-pass filter. Microparticles, vesicles, and microbubbles less than 8 µ can freely pass from the venous to the arterial side. **(B, C)** The lung as a diffusion filter. A bubble squeezes into an alveolus capillary to form a gas tube. Normally, gas diffuses from the tube into the alveolus until the bubble collapses. If the tube is too long, it can reach the end of the capillary and dump gas into the arterial system. **(D)** Two possibilities for shunting the bubble through the lung: non-perfused alveoli and IPAVA. **(E)** Four ways of passing bubbles through the lung to the arterial side. **(F)** Proposed model for linking VGE and arterial bubbles.

Gas-containing MPs may cross freely, while a microbubble with adherent debris may lose its gas in the alveolus and continue as a gas-poor vesicle. Thus, filtration should be considered in terms of both particle size and gas diffusion, not simply complete retention of every circulating object.

### 8.2 Concept: lungs as a diffusion-limited filter

For bubbles larger than the filtration threshold, filtration is based on diffusion. When a bubble reaches the alveoli, it squeezes into the capillary and forms a gas tube. This increases the diffusion area and allows the excess gas to be eliminated into the alveoli (Hennessy, 1989b).

Hennessy proposed a full mathematical description of this diffusion model (Hennessy, 1989b). However, diffusion requires time. If the gas from the bubble is pushed into the capillary too rapidly, or if the bubble gas pressure is too high, the gas tube can reach the end of the capillary and dump gas directly into the arterial side (**Figures 12B, C**). Assuming the dimension of the incoming microbubbles remains the same, this physical model suggests that:

- Helium diffuses faster than nitrogen, so a helium bubble in an alveolar capillary should lose gas faster and be filtered more effectively than a nitrogen bubble of the same size. This concerns pulmonary bubble clearance only. Heliox decompressions are not necessarily shorter because helium also loads tissues faster; deep-bounce heliox schedules may be longer than comparable air schedules, and gas switching is constrained by isobaric counter diffusion.
- Microbubble inert-gas content depends on ambient pressure, and diffusion from capillary to alveolus should take longer at greater depth. This may help explain why deep technical divers using trimix or heliox select lower gradient factors for more conservative decompression.

This model also introduces the possibility of linking lung filtration to two diving situations:

- A high dose of incoming VGE will likely cluster the alveoli, increase pulmonary blood pressure, and accelerate the blood flow through the alveolar capillaries. This reduces the transfer time available for diffusion and allows microbubbles to pass into the arterial circulation (Butler and Hills, 1985; Vik et al., 1990).
- Exercise increases the pulmonary blood pressure and reduces the alveolar transfer time. This could account for Madden et al., who showed that post-dive exercise increases the number of arterial bubbles detected after scuba diving (Madden et al., 2013).

### 8.3 Concept: shunts in the lungs or IPAVA

Another mechanism for passing bubbles through the lungs is the presence of lung shunts.

Lung ventilation and perfusion adapt to metabolic needs; however, exchanges can be reduced in some situations (right-to-left shunting of venous blood, diffusion-limited alveolar-capillary oxygen exchange, and ventilation-perfusion inequality). It is generally considered that the venous blood shunted through the lung at rest is less than 1%, but can increase with immersion (Derion et al., 1992). Pulmonary oxygen toxicity reduces alveolar gas exchange, as indicated by measurements of the diffusing capacity of carbon monoxide (Clark, 1993). The ventilation-perfusion inequality increases with age (Cardús et al., 1997). If perfused alveoli are not ventilated, blood passes through the lungs without exchanging gases (**Figure 12D**). A bubble could be conveyed into a capillary thread, leading to non-ventilated or poorly-exchanging alveoli and, hence, a chance to bypass the lung.

Madden et al. have imagined an IPAVA that could open in specific situations, such as post-dive exercise or a high VGE dose (Madden et al., 2015). The failure mechanism for the diffusion filter model is the same, making it difficult to differentiate between the two hypotheses.

Ljubkvic et al. found bubbles in the left heart cavities of divers previously screened negatively for patent foramen ovale (PFO) after decompression without procedure violation, but with a high VGE score in the right heart (Ljubkovic et al., 2010). The authors explained their results by pointing to increased pulmonary pressure, which expands the capillaries and opens IPAVA.

### 8.4 Concept: right/left cardiac shunt

Since the early 1990s, the presence of a right-to-left shunt has been identified as a major risk factor for DCS in diving (Moon et al., 1989). Numerous case reports have illustrated the so-called “undeserved DCS,” occurring after dives within the accepted decompression limits and without violating accepted decompression procedures, but associated with a right-to-left shunt (Germonpré et al., 1998; Sykes and Clark, 2013; Lee et al., 2023). A PFO plays a critical role in arterial embolization, leading to inner ear DCS (Mitchell and Doolette, 2015).

### 8.5 Implication: increased CO_2_ level

CO_2_ levels may increase during a dive because of operational conditions (e.g., physical effort, work of breathing, gas density) and may lead to hyperventilation and CO_2_ retention.

Diving situations that produce CO_2_ retention and hypercapnia have always been associated with a higher risk of DCS:

- Anxiety and stress, either because of the dive conditions or of insufficient training.
- Exhaustion or hyperventilation due to intense activity or work at the bottom (Madden et al., 2015).
- Cold at the bottom or during decompression (Gaustad et al., 2021).
- Breathing resistance due to the poor performance of the regulator (bad maintenance, wrong adjustment, insufficient flow).

High PCO_2_ actually increases pulmonary vascular resistance (Dorrington et al., 2010). Hence, it is reasonable to consider that such a situation could either affect the lung transfer function or open a PFO.

### 8.6 Implication: pulmonary pressure increase

Some diving situations can increase pulmonary pressure, reduce the trapped bubble size, and bypass the bubble. For instance (**Table 4**):

- An excessive Valsalva maneuver. This is typical of a beginner diver with difficulty controlling their stop depth.
- Pressure variations of divers hanging on a dive line during a decompression stop with a heavy swell.

**Table 4:** Four diving situations suspected to affect the lung transfer function and favor shunted arterial microbubbles.

Dive conditions	Effects	Consequences
Hard work at bottom, stress, cold during decompression, breathing resistance, hyperventilation.	Hypercapnia, increased alveolar capillary diameter.	Larger threshold diameter, shorter diffusion time, opening of IPAVA.
Exercise after decompression.	Higher levels of CO_2_, higher pulmonary blood pressure.	Larger threshold diameter, shorter diffusion time, opening of IPAVA.
High level of incoming VGE.	Higher pulmonary blood pressure.	Shorter diffusion time, opening of IPAVA.
Valsalva during decompression, Decompression in heavy swell, Ladder climbing at the end of the dive.	Increased pulmonary pressure, Reduce bubble size.	Squeeze bubbles into the arterial side.

Down arrow: decrease, up arrow: increase

The mere presence of bubbles in the pulmonary circulation increases the pulmonary artery pressure (Vik et al., 1993b). In a pig model, animals with a PFO had lower pulmonary artery pressures and higher arterial bubble counts than those without a PFO (Vik et al., 1993a).

The model also accounts for heavy efforts involving closed-glottis thoracic overpressure, such as climbing the ladder at the end of the dive with full gear, which reduces and then relaxes the venous return (Balestra et al., 1998). Such venous blood variations could pass bubbles trapped in the lung into the arterial side.

### 8.7 Proposed model: the lung bubble transfer function

We propose to translate the above concepts into a module called the lung bubble transfer function. This function defines the lungs’ capacity to stop and eliminate VGE during a given decompression (**Figure 12F**).

Right-to-left shunts are excluded from this function. Although cardiac shunts are believed to be more important than the other pathways, they are an individual factor that cannot be considered in a “standard diver.” Based on retrospective data on diving accidents, several attempts have been made to quantify the increased risk of diving for divers with a PFO (Bove, 1998; Torti et al., 2004). In these papers, an increased incidence of DCS of the order of 3%–6% has been reported, although the latter figure has been criticized due to the subjective criteria used to define DCS (Torti et al., 2004).

Some decompression algorithms, such as the VPM, track the total volume of venous gas produced and keep it below a critical threshold. These algorithms could propose a simplified version of this lung transfer function in which the risk of arterial embolization (and DCS) would be proportional to the volume of incoming VGE (Hugon, 2014).

### 8.8 Perspective: relation between VGE grades and DCS risk

A simple view of the relationship between VGE and DCS would be that:

- The VGE grade is the “signature” of the diver. It provides information on the diver’s age, fitness, previous dive history, and pre-dive conditioning.
- A “good diver is a good bubble filter”. When VGE are stopped at the level of the lungs and filtered by the alveoli, they remain asymptomatic.

Vann tried to further characterize lung filtration by establishing the relationship between Doppler bubble grade and the incidence of DCS for air diving, altitude exposures, and nitrox saturation diving. He found that DCS risk is low with low bubble grades and increases with higher grades. He concluded that low bubble grades are a good indicator of decompression safety, but that high grades are poor predictors of DCS (Vann et al., 2005).

Doolette confirmed, using data from the US Navy, that VGE detected by two-dimensional echocardiography are an imperfect surrogate endpoint for DCS (Doolette, 2016).

We identify four scenarios that could explain the relation between the VGE grade and the risk of DCS (**Figure 12E**). When lung filtration capacity is exceeded, VGE are shunted through the heart, and microbubbles arrive on the arterial side. We believe that arterial embolization, rather than VGE grades, is the true signal of bubble scenarios leading to DCS, as discussed below.

From this hypothesis, it is possible to deduce a dose-response curve linking the VGE bubble grades and the shunted arterial microbubbles where:

- If the incoming VGE grade is low, the lungs can stop and filter out the venous bubbles. VGE have no direct implication for the safety outcome of the decompression (Nishi et al., 2003).
- If the incoming VGE grade is high, it can increase the pulmonary arterial blood pressure and possibly force bubbles into the arterial side. Given the role of shunted arterial microbubbles, massive arterial embolization may coincide with a high risk of DCS.

This could explain the deceptive attempts to associate the number or grade of detected VGE with the risk of DCS.

## 9 From arterial microbubbles to target organs

We have identified so far two scenarios that produce arterial microbubbles:

- The microbubbles potentially produced on the distal arterial/arteriole AHS after a rapid pressure reduction. They are called distal arterial microbubbles.
- The arterial microbubbles shunted through the lung or the heart from incoming VGE. They are named shunted arterial microbubbles.

These arterial microbubbles, filled with venous gas and surrounded by arterial gas, are in survival mode.

### 9.1 Concept: survival distance of shunted arterial microbubbles

Hennessy explained that a venous bubble passing through the lungs and entering the arterial system is likely to dissolve rapidly (Hennessy, 1989a). The bubble contains venous gas, and once it passes through the lungs, the excess gas diffuses into the surrounding arterial blood. Hennessy estimated that the distance a shunted arterial bubble must travel to reach critical size and collapse is approximately 30 cm to 40 cm. He defined this distance as the bubble survival distance. This distance depends on the number of molecules initially present in the arterial microbubble and, therefore, on depth.

During PFO echocardiography, large quantities of air bubbles may reach the left atrium after injection of agitated saline, yet neurological symptoms are rare. Because the brain has high metabolic demand and a high OW, cerebral tissues may tolerate circulating bubbles by remaining relatively “unsaturated” through the OW mechanism.

It was shown that very few exercise-induced arterialized gas bubbles reach the cerebral vasculature (Barak et al., 2015). However, if an arterial microbubble were to reach the brain while it is supersaturated, the bubble would draw gas from the surrounding, stop shrinking, and possibly grow to a size sufficient to change local perfusion (**Figure 13A**).

**Figure 13:**
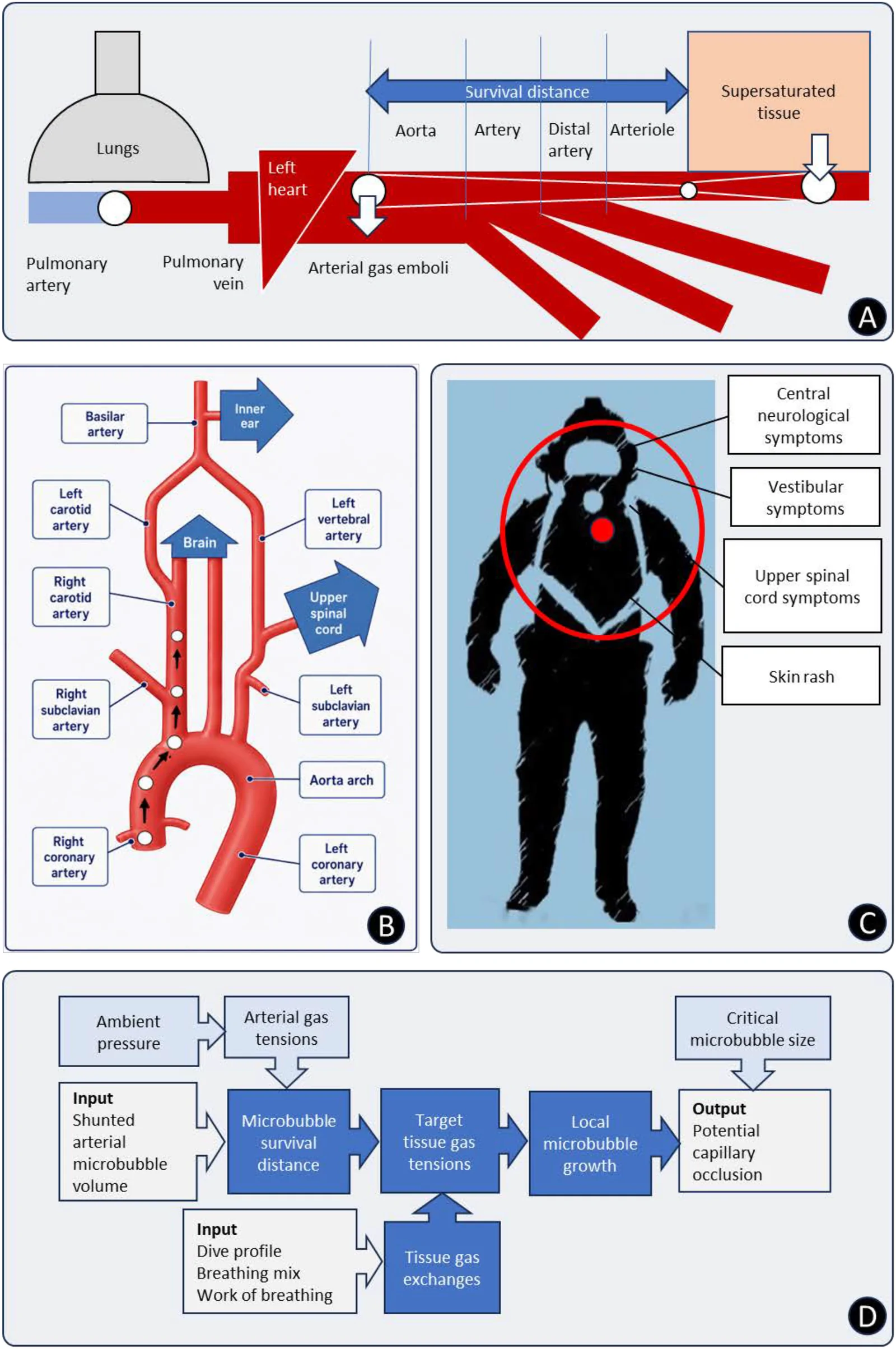
**(A)** The survival distance limits the range of action of the shunted arterial bubbles. **(B)** The privileged transit of an arterial bubble in the aortic arch. **(C)** The range of action of shunted arterial bubbles estimated by a 30–40 cm circle around the left heart. **(D)** The shunted arterial microbubble function.

Goldman et al. numerically computed the dissolution time of a bubble by solving the full diffusion equation for a spherical geometry (Goldman et al., 2017). The result depends on the initial bubble size; for a radius of 10 microns, consistent with lung filtration capacities, the dissolution time ranges from 5 to 40 s. The authors then estimated the arterial distance from the left ventricle to several target organs. They determined the transit time of an arterial bubble to these organs at different arterial blood velocities. They found that the transit time from the left heart to the inner ear was approximately 2 s, within the survival time they calculated for the arterial microbubbles.

The survival distance concept explains:

- The prevalence of right-to-left shunts in DCS symptoms associated with arterial embolization. A cardiac shunt can pass much larger bubbles into the systemic circulation than the lungs can through capillaries. Consequently, these larger arterial bubbles have longer survival times and greater capacities to reach a target organ.
- The effect of depth. Hydrostatic pressure determines the number of gas molecules in the microbubble volume when it leaves the left heart. The greater the depth, the longer the survival time of the shunted arterial microbubble.
- The nature of the gas, which directs the inward/outward diffusion rate.

### 9.2 Concept: the target organs

Hills and James were the first to identify the role of arterial microbubbles. They injected calibrated microbubbles into guinea pigs at the surface, showing consecutive damage to the brain blood/brain barrier (Hills and James, 1991). They concluded that arterial embolisms could cause a gradual onset of neurological symptoms by disrupting endothelial function and inducing slow edema development in the area.

Arterial embolization designates certain organs as the privileged destination for shunted arterial bubbles. This is related to the “piping” of the blood vessels, branching directly from the aortic arch that sends blood by centrifugation into a few specific arteries (**Figure 13B**). The organs that are likely to be reached by arterial bubbles include:

- The brain through the carotids.
- The upper part of the spinal cord through the left subclavian artery.
- The inner ear through the vertebral and basilar arteries.
- Other surrounding areas, such as the skin on the torso, can be affected by a shower of arterial emboli.

If one of these organs is supersaturated when the arterial bubbles reach the place, it will feed bubble growth and initiate a critical insult scenario for DCS. Such an organ at such a time is defined as a target organ that arterial bubbles reach before they collapse, thereby feeding local growth and causing DCS symptoms.

Target organs will not become supersaturated simultaneously; instead, they will play their critical role sequentially during decompression.

### 9.3 Proposed model: the shunted arterial microbubble survival

We propose to translate the above concepts into a module called the microbubble survival function. It defines the survival distance of an arterial microbubble passed into the arterial circulation (**Figure 13D**).

### 9.4 Perspective: the critical distance from the left heart

The first pathway to DCS only covers the physical role of bubbles based on two events:

- The development of VGE and their possible transfer to the arterial circulation as a shunted arterial bubble.
- The development of arterial bubbles from distal arterial AHS.

Both occurrences result in the same critical insult: upstream tissue embolization. Yet, the difference is in the action range:

- A venous microbubble arriving on the arterial side is limited to its survival distance. This defines a circle with a 30–40 cm radius around the left heart. This area includes specific DCS symptoms, such as vestibular hit, central and spinal neurological symptoms, and skin problems. The brainstem falls within that radius.
- Beyond this radius, the bubble will collapse. A shunted arterial bubble cannot be held responsible for knee pain.
- When it detaches, a microbubble produced at a distal arterial AHS level remains stable. It is not “surviving” in its surrounding arterial blood. Its range of action is extended because it can reach any body part.

The concept of the target organs defines a critical radius around the left heart that delimits the range of action (the survival distance) of shunted arterial bubbles (**Figure 13**). This radius depends on the survival distance and, therefore, on depth, but ranges from 30 cm to 40 cm.

## 10 Extravascular bubbles

Tissue bubbles have been detected during decompression (Papadopoulou et al., 2015). In tissue, surface tension and tissue elasticity stabilize the gas phase (Strauss et al., 2018b). Tissue gradients are expected to be lower than those across the blood vessel endothelium, and tissue gas nuclei will grow more slowly. When extravascular bubbles participate in DCS, their effect takes longer to appear.

Lymphatic bubbles also exist, drained from the interstitial fluid, slowly transferred by the lymph, and finally injected into the bloodstream at the end of the thoracic ducts (Hugon et al., 2009; Balestra, 2014). This pathway could explain the detection of VGE long after decompression in purely lymphatic forms of DCS.

### 10.1 Concept: the extravascular volume of gas released

Extravascular AHS that support gas nuclei exert a local effect. The physiological damage they can cause is related to the volume of gas released. The problem is identifying the tissues in which gas-phase growth occurs. Considering musculoskeletal symptoms as the only endpoint, the decompression model can be reduced to one tissue representing the joint (**Figure 14**).

**Figure 14:**
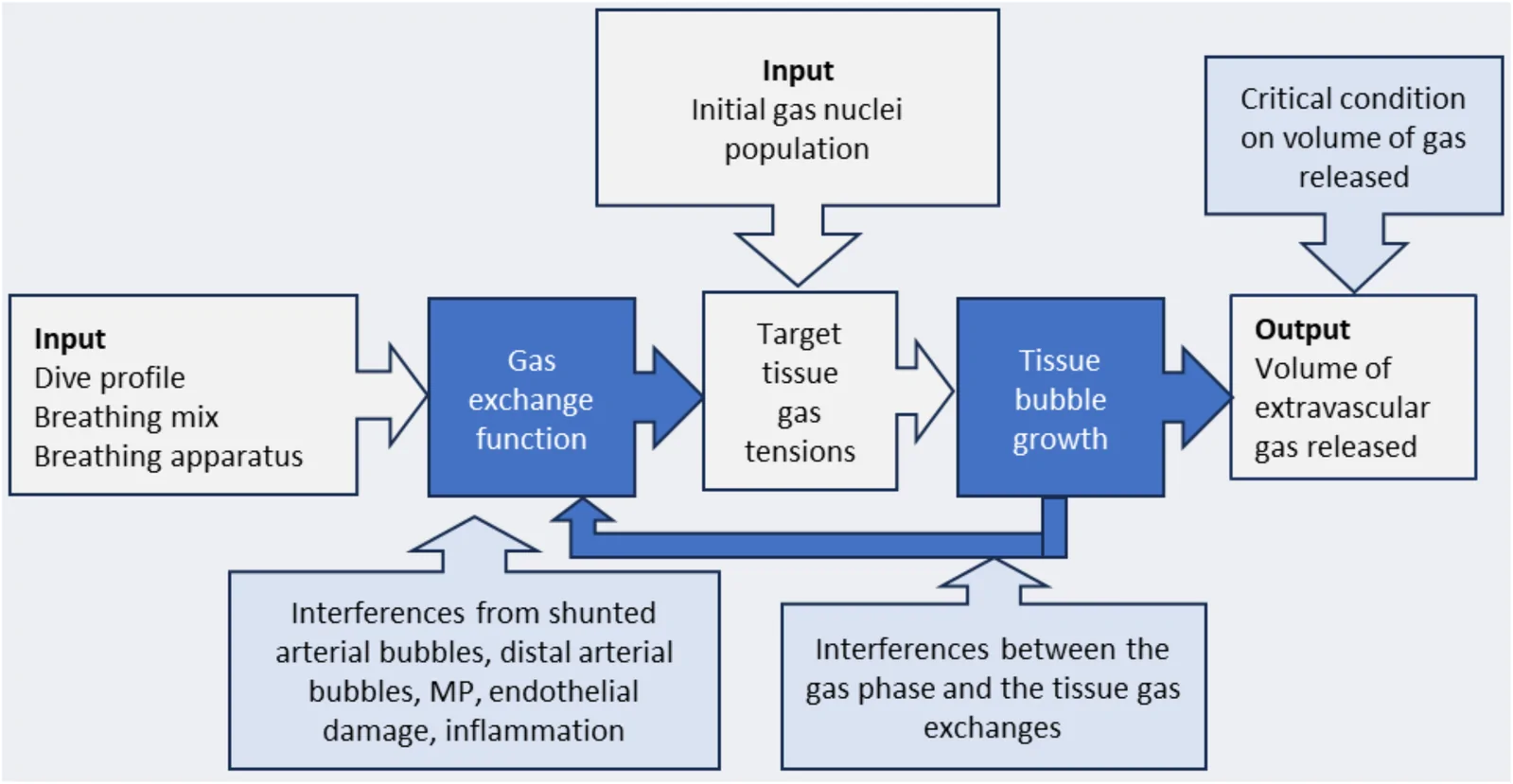
Definition of extravascular bubble growth function.

Hempleman used the square root of time to approximate the diffusion equation and obtained an excellent fit of no-stop decompression with only one compartment (Hempleman et al., 1952). The Royal Navy then investigated a single tissue model based on a semi-infinite slab for which Rashbass derived the gas exchange equations (Rashbass, 1955). Finally, Hills proposed a thermodynamic model based on a bulk diffusion tissue slab with a spatial dimension that permitted separating the primary insult (phase separation) from the critical insult (volume of gas released) (Hills, 1968; Hills, 1977).

Hugon et al. reviewed decompression models and identified several algorithms using a critical volume of gas released as a safe ascent criterion (Hugon, 2014). These models include, for instance, Hempleman and Hennessy’s critical volume assumption (Hennessy and Hempleman, 1977), which assumes an instant equilibrium between the gas phase and the tissue, or Vann’s model, where gas phase formation interferes with tissue gas exchanges (Vann, 1982).

We postulate that a shunted arterial bubble may perturb tissue gas exchange without occluding flow by altering perfusion and diffusion gradients; repeated passages could amplify the effect. MP-induced endothelial damage and systemic inflammation may act similarly, as discussed later.

### 10.2 Proposed model: the extravascular bubble growth

We propose to translate the above concepts into a module called the extravascular bubble growth function (**Figure 14**). It combines a gas-exchange model with a tissue-bubble growth model. The function input parameters include:

- The dive profile.
- The breathing mix or the diluent mix supplied.
- The breathing apparatus used with its settings.
- The diver’s bubbling function.

The function adjustment parameters include:

- The parameters required for the definition of the diffusion (or combined perfusion/diffusion model) at the location of the nanobubble.
- The parameters of a bubble growth model.
- A possible adjustment parameter after changes in the local perfusion or diffusion.

The function output is:

- The volume of the released gas phase.
- This output is to be compared to a critical volume of released gas, considered a threshold for musculoskeletal symptom onset, according to the critical volume hypothesis of Hennessy and Hempleman (1977).

## 11 Inflammation

Experience with commercial air diving worksites suggests that divers’ susceptibility may change after repeated exposures. Divers performing similar work at similar depths and using the same tables started developing DCS after several weeks. The divers called it “decompression fatigue”, which rightly captures the cumulative nature of the stress and justifies a recovery.

In a puzzling experiment, Yang et al. subjected mice to severe decompressions and sampled blood MP from animals with severe neurological symptoms after the dive. They then isolated these MP and injected them into a second group of mice, free of any dive exposure. The mice in this second group displayed the same endothelial symptoms as if they had been subjected to a decompression (Yang et al., 2012). Elevations in pro-inflammatory MP have since been associated with decompression, and MP were shown to be responsible for vascular injuries in an animal model (Thom et al., 2011). This suggests that the inflammatory process responsible for divers’ decompression fatigue has a material basis.

### 11.1 Concept: MP produced by bubble detachment from AHS

We have already discussed that a microbubble detaching from the AHS tears the substrate and damages the endothelial membrane (Arieli, 2017), resulting in (**Figure 15A**):

**Figure 15:**
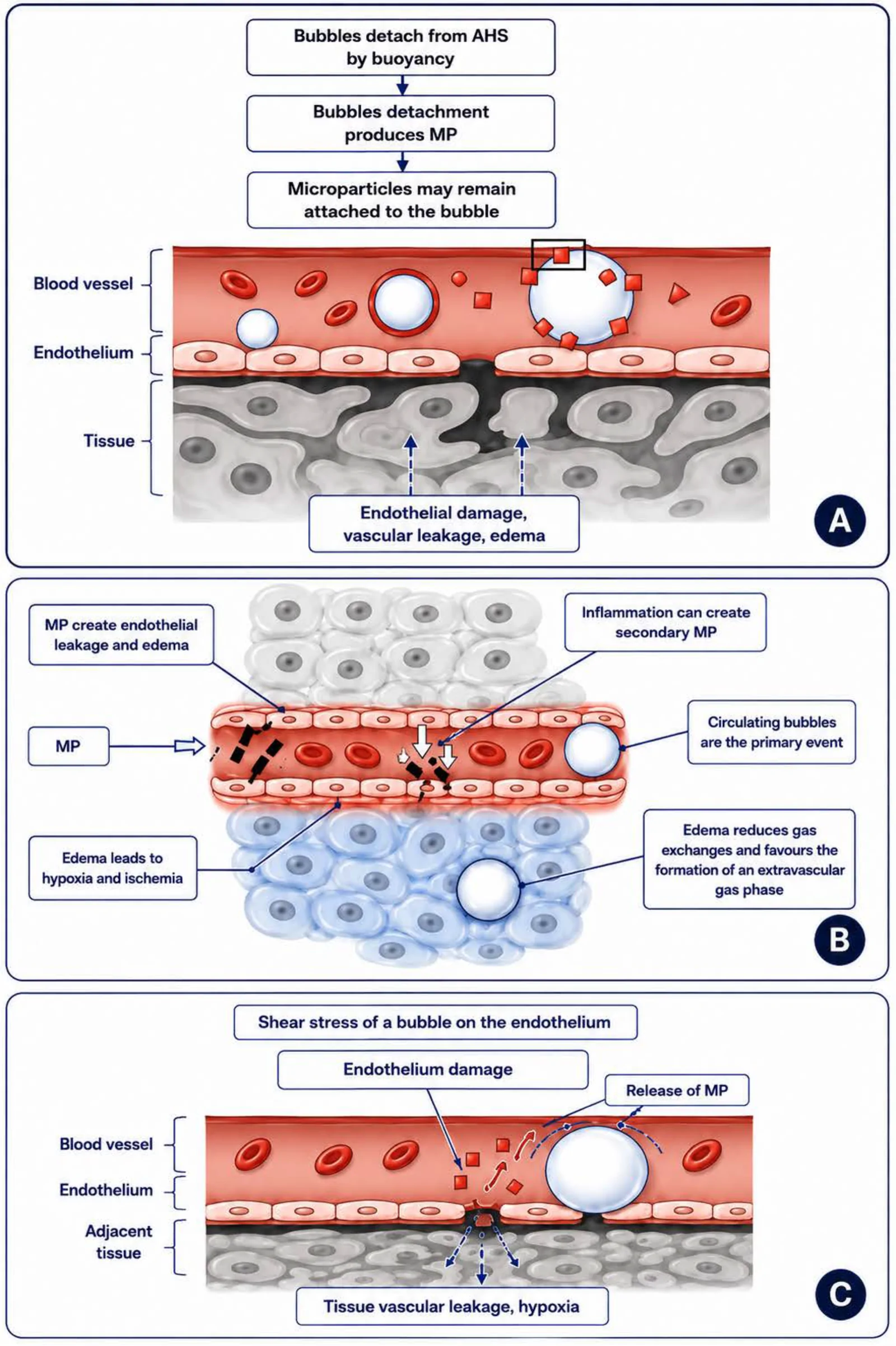
**(A)** MP produced by bubbles can trigger a cascade of events leading to endothelial damage, tissue hypoxia, extravascular bubbles, and secondary MP production. **(B)** Shear stress produced by a bubble against the vessel wall, causing local endothelium damage, vascular leakage, edema, and hypoxia. **(C)** Bubble detachment from the AHS during decompression, producing MP and MV, endothelial damage, vascular leakage, and edema.

- Blood-borne MP from the debris of the AHS or the underlying endothelial membrane.
- Local endothelial damage and dysfunction.
- Microbubbles wrapped with the debris of endothelial cells crossing the lung capillary as gas-containing MPs.

A given AHS can continuously generate bubbles and, therefore, sustain MP production during decompression, thereby increasing endothelial damage.

### 11.2 Concept: MP produced by circulating bubbles

The continuous production of microbubbles and VGE is expected to have three consequences.

First, circulating bubbles cause mechanical damage to the lining of downstream blood vessels by shear stress (**Figure 15B**). Hills et al. injected tiny microbubbles (15 microns) into the carotid of guinea pigs and later found evidence of blood–brain barrier dysfunction and focal edema (Hills and James, 1991).

Second, circulating bubbles interact with the endothelium triggering platelet aggregation on the bubble surface and the formation of microthrombi (Eckmann and Armstead, 2006). Platelet aggregation and complement activation have been associated with circulating bubbles in decompression (Thorsen et al., 1993; Pontier et al., 2008). These processes induce vasoconstriction, endothelial dysfunction, and tissue edema, reducing tissue gas exchange and favoring further bubble growth (**Figure 15C**).

Third, circulating bubbles and platelet aggregation trigger neutrophil activation, generating secondary MP.

### 11.3 Concept: MP produced by oxidative stress

Reactive oxygen and nitrogen species reactive oxygen species/reactive nitrogen species (ROS/RNS) are normal products of aerobic metabolism. Mitochondria are a major ROS source, with additional production by enzymes including NADPH oxidases and xanthine oxidase. Exogenous stresses such as tobacco, pollution, smoke, and drugs can further increase oxidative stress and tissue damage.

In diving, the list of potential stresses is long:

- Confinement, hydration, diet, and sleep pattern changes.
- Hard physical work, dense gas breathing, cardiovascular changes.
- Psychological pressure related to work performance.
- Thermal balance.

Diving has been shown to generate ROS, like any other stressful human activity (Brubakk et al., 2005; Obad et al., 2010). The presence of ROS triggers an antioxidant response (total antioxidant capacity or total antioxidant capacity), and specific scavengers (catalase, glutathione peroxidase, and superoxide dismutase) are produced to control the ROS level (Sureda et al., 2009). Oxidative stress corresponds to an imbalance between ROS production and the antioxidant response. When cellular defenses are exceeded during episodes of oxidative stress, the consequences include (Juan et al., 2021) (**Figure 16A**):

**Figure 16:**
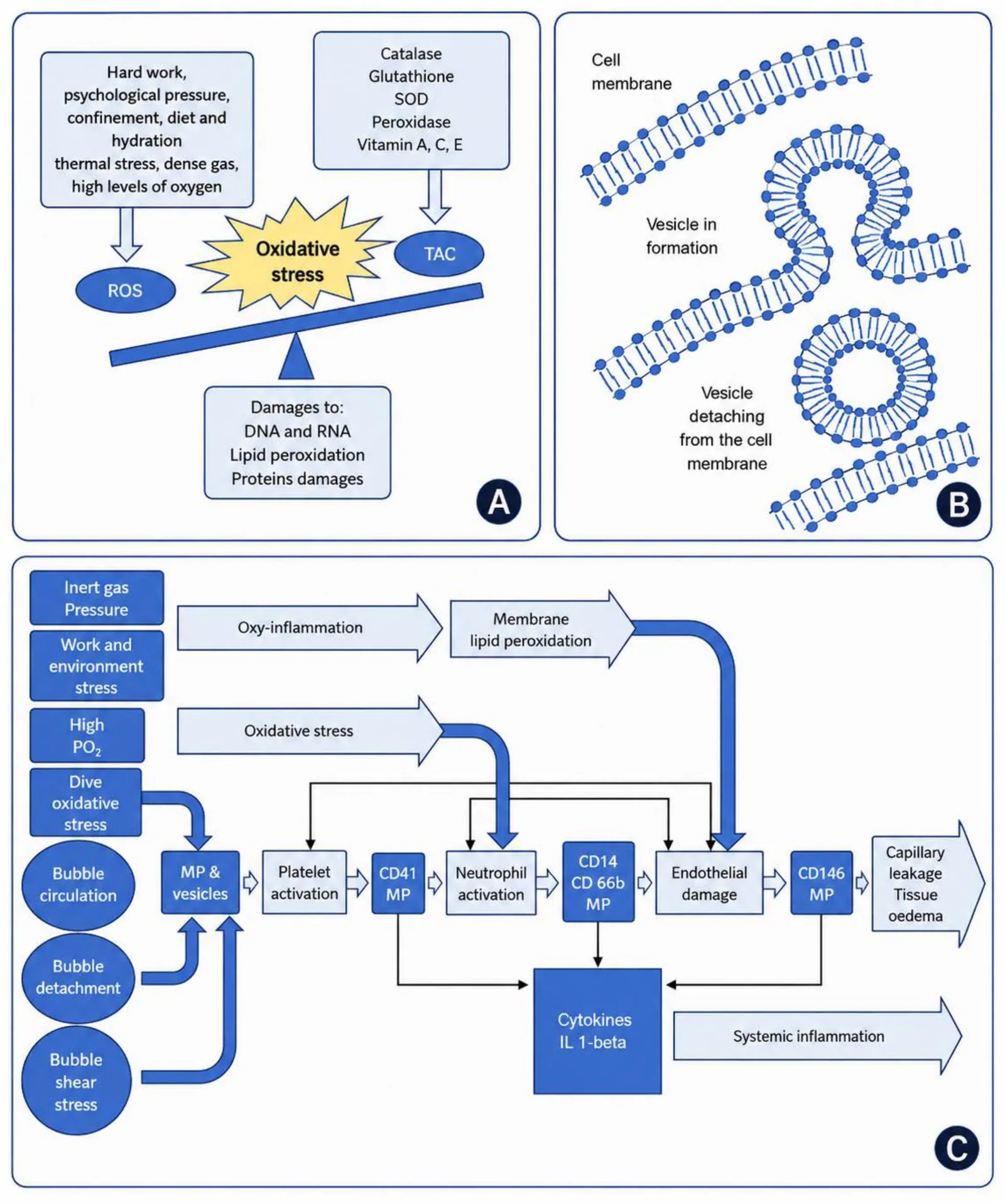
**(A)** A simple definition of oxidative stress: ROS, Reactive Oxygen Species; TAC, Total Antioxidant Capacity. **(B)** Cell membrane vesicles formed by lipid peroxidation following oxidative stress. **(C)** Events triggered by oxidative stress and bubbles with relevant biomarkers.

- Damage to DNA or RNA.
- Lipid peroxidation of polyunsaturated fatty acids (such as membrane phospholipids).
- Oxidation of proteins.

During oxidative stress, ROS damage the cell membrane by peroxidation of the phospholipid bilayer, creating vesicles. MP corresponding to lipid bilayer vesicles are present in every body fluid. Their number increases with inflammation (**Figure 16B**).

Vesicles are divided into three sub-groups.

- Exosomes > 20–120 microns from the endosomal pathway.
- Microparticles > 0.1–1 micron, a bulging expression of cellular membranes.
- Apoptotic bodies > 0.5–5 microns, generated after cellular self-destruction.

Exosomes and vesicles play a role in cell-to-cell communication due to their contents (nucleic acids, inflammatory mediators, enzymes, or organelles that generate free radicals). MP can stimulate the release of proinflammatory cytokines, and platelet-derived MP stimulate leukocyte activation and aggregation. Annexin V–positive platelet MP exhibit procoagulant activity (Thom et al., 2011). Consequently, pro-inflammatory biomarkers such as interleukin IL-1β or IL-6 are released (Arya et al., 2023; Thom et al., 2023a).

Because the dive and decompression trigger events leading to MP production and inflammation, circulating MP are increased in humans subjected to simulated or actual scuba diving (Madden et al., 2010; Thom et al., 2012).

### 11.4 Concept: MP produced by breathing elevated PO_2_

Prolonged exposure to hyperoxia produces oxidative stress, which leads to cellular damage, lipid peroxidation, enzyme inactivation, and oxidation of biological molecules (Vezzoli et al., 2024).

It has already been shown that the partial pressures of oxygen associated with long exposures, such as saturation diving (Mrakic-Sposta et al., 2020) or deep rebreather dives, contribute to MP production (Bosco et al., 2018; Arya et al., 2023; Thom et al., 2023a).

The fraction of inspired oxygen may also influence inflammation. Balestra investigated the metabolic responses of subjects exposed to oxygen levels from 10% to 100% and to pressures above ambient, up to 250 kPa. We analyzed MP-expressing proteins from different origins and found distinct and sometimes contradictory responses (Balestra et al., 2022).

### 11.5 Concept: MP produced by inert gas pressure

Inert gas pressure may create MP before decompression. Bhullar et al. investigated the MP production mechanism in human and mouse platelets exposed to high inert-gas pressure. Results indicated that MP production occurs via an oxidative stress response in a dose-dependent manner (Bhullar et al., 2016). Although this study does not clarify whether PO_2_ or the partial pressure of inert gas was elevated, it appears that high pressures of N_2_, Ar, and He can enhance singlet O_2_ generation from superoxide. An electron transfer from molecular collisions, similar to that observed in atmospheric chemistry, may explain this phenomenon (Hild and Schmidt, 1999). In summary, the dose-response of gases and hydrostatic pressure indicates that gas molecular characteristics determine whether platelets generate MPs. The mechanism could involve gas interactions with superoxide from NOX activity, generating singlet O2, which initiates the events.

Studies with human volunteers have demonstrated that there are elevations in MP numbers and those expressing filamentous (F-actin) on the membrane surface while under pressure and before decompression (Brett et al., 2019; Bhopale et al., 2021).

The problem with experiments involving exposure to pressure and inert gases before decompression is that small pressure changes, such as exposure to 125 kPa on air, can also modulate cytokine levels (MacLaughlin et al., 2023). The inflammatory effects of inert gas/pressure are reported in manned experiments at low pressure and in the absence of increased PO_2_ (Balestra et al., 2024a). We acknowledge that the current literature does not permit modeling their impact on decompression procedures.

### 11.6 Concept: MP and inflammation biomarkers

Six mechanisms of MP production have been listed that involve the dive, the decompression, and the gas mixture (**Figure 16C**).

This primary MP generation triggers a cascade of events leading to platelet and neutrophil activation and endothelial dysfunction.

The MP are characterized by the surface expression of antigenic markers from the cells of origin: platelets (CD41), neutrophils (CD66b), monocytes/neutrophils (CD14), endothelial cells (CD146), and erythrocytes (GlyA). This allows postulation of their implication in the inflammatory process (**Figure 16**).

### 11.7 Concept: systemic inflammation

MP are known to trigger a series of biomarker expressions, particularly cytokines, which are signaling proteins that help regulate inflammation in the body. Among the cytokines associated with MP, Interleukin-1-beta (IL-1-Beta) causes diffuse capillary leakage in the mouse model (Thom et al., 2018).

Elevated levels of IL-1-Beta have been detected in neutrophil MP in subjects exposed to 60 min at 18 msw and 30 min at 30 msw before decompression (Brett et al., 2019).

After a week of deep diving to 100 msw, blood-borne extracellular vesicles and inflammatory biomarkers were evaluated in closed-circuit rebreather (CCR) divers. Elevated levels of IL-1-beta and plasma gelsolin (pGSN) were measured after 7 days (Arya et al., 2023; Thom et al., 2023a).

### 11.8 Implication: endothelium dysfunction

Changes in endothelial function during diving were first published by Brubakk et al., who measured flow-mediated dilatation (Brubakk et al., 2005). This effect was later confirmed in saturation divers after decompression. It has been shown that diving stress also produces RNS that convert NO to NOx. Consequently, NO is depleted, and vascular function is impaired. Wisloff et al. further demonstrated that inhibition of the NO synthase induces significantly higher bubble production in decompressed rats (Wisloff et al., 2003). NO depletion will also activate coagulation (Pontier et al., 2012) and facilitate the adhesion of leucocytes (Martin and Thom, 2002). This endothelial dysfunction impacts the decompression process (Brubakk and Mollerlokken, 2009) and:

- Changes the nucleation process for the endothelial AHS.
- Impairs tissue gas exchange and reduces the oxygen supply.
- Increases the diffusion distance and favors extravascular gas phase formation.
- Decreases the blood capillary section and facilitates the blood flow occlusion by an arterial bubble.

This alters the diver’s susceptibility to the various physical bubble scenarios. Moreover, it could cause endothelial damage, leading to symptoms similar to DCS without any bubble intervention, as in the murine model published by Thom et al. (2011). The process requires time. It should be most effective in operational conditions such as repetitive and multiday diving. It is expected to act during long saturation decompressions, causing joint pain at the end of saturation decompression.

### 11.9 Implication: brain inflammation

The brain is particularly susceptible and vulnerable to inflammation. ROS, but also RNS and NO, influence the cerebral vascular tone and permeability, potentially inducing edema (Salvagno et al., 2024). The brain is the primary target organ of decompression-induced inflammation.

Thom et al. investigated the glial-based lymphatic (glymphatic) function and its role in a murine model of DCS. Blood-borne MP expressing filamentous actin on their surface were detected, which exacerbates neutrophil activation. Activated neutrophil sequestration disrupted blood–brain barrier integrity, causing further astrocyte and microglial perturbation (Thom et al., 2023b).

pGSN is a cytoplasmic actin-binding protein that can depolymerize filamentous actin (F-actin), bind to inflammatory mediators, and enhance macrophage phagocytosis. Blood pGSN levels fall during acute and chronic inflammation. The magnitude of pGSN depletion indicates the extent of tissue damage.

In the CCR divers monitoring study in the Red Sea, already mentioned, increased levels of gelsolin and IL-1β were also measured and were associated with reduced pGSN levels (Arya et al., 2023). During a dive, MP expressing transmembrane protein 119 and thrombospondin-1 (TSP) are released via the glymphatic system into the circulation and activate neutrophils, which generate a second array of MP containing F-actin. The observed reduction of gelsolin was assumed to be caused by its binding and lysis of F-actin expressing MP (**Figure 17A**).

**Figure 17:**
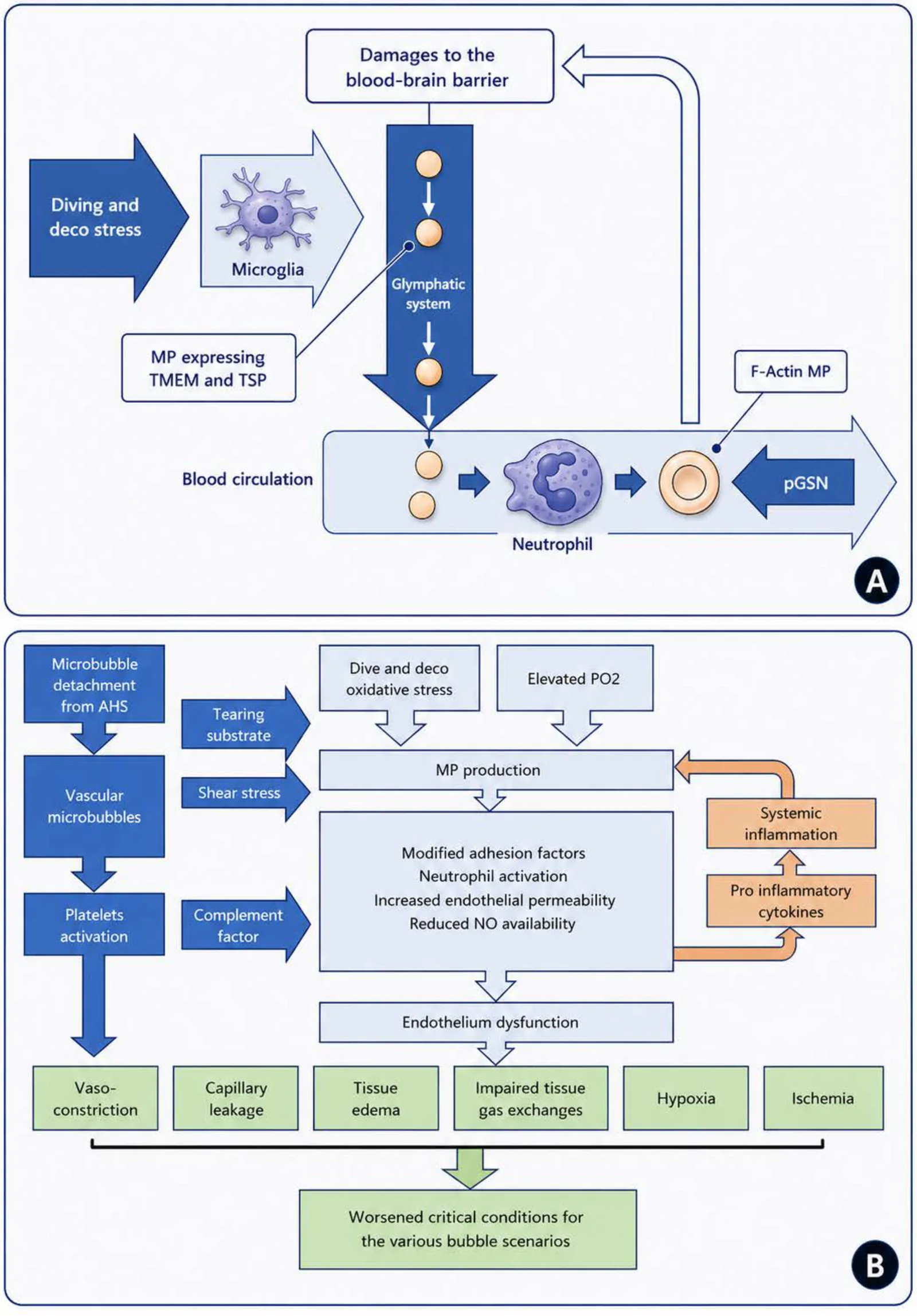
**(A)** Brain neuroinflammation: MP-expressing TMEM and TSP are produced by the microglia and released into the blood circulation via the glymphatic system. There, neutrophils convert them into F-Actin MP. **(B)** A tentative description of inflammation’s role in modifying the bubble scenarios.

### 11.10 Proposed model: the inflammation stress

The development of inflammation obviously takes time and involves multiple processes and interactions as illustrated in **Figure 17B**. Ⓐ Brain neuroinflammation: MP-expressing TMEM and TSP are produced by the microglia and released into the blood circulation via the glymphatic system. There, neutrophils convert them into F-Actin MP. Perspective: Management of saturation intervals.

Stress is part of everyday life, and the body can manage stress, provided:

- The stress does not exceed the resilience of the body.
- Enough time is available for the body to recover from the stress.

Considering diving as a cumulative stress exposure justifies the classic method of stress management and fatigue recovery:

- Training and experience are essential because they teach divers how to pace their work, adjust their breathing rate, and manage their occupational stresses.
- Fitness is essential because it helps divers to better adapt to oxidative stress and cope with the additional diving load. Fitness evaluated through VO2 max has been the main criterion for selecting deep divers operating in Brazil (Imbert, 2005).
- Rest periods must be included between dives to allow the diver to recover from the diving stress exposure.

The diving companies have long-established empirical rules for diving intervals. In saturation diving, they conveniently refer to industry standards, that is, the maximum duration and minimum interval between saturations defined in the DMAC guidance note 21 (DMAC, 2006).

The efficiency of these standards has been documented by on-site monitoring of divers, which confirmed the presence of decompression stress and the recovery of vascular function within 12 hours following the end of the decompression.

The North Sea diving population is also aging. **Figure 18A** displays the age distribution of saturation divers working on a large diving support vessel in 1979. The oldest diver was 63. The fact that he can still renew his diving certificate is a good indicator of the adequacy of the current rest patterns (Imbert et al., 2024b). The concern, however, remains when to decide to quit a diving career.

**Figure 18:**
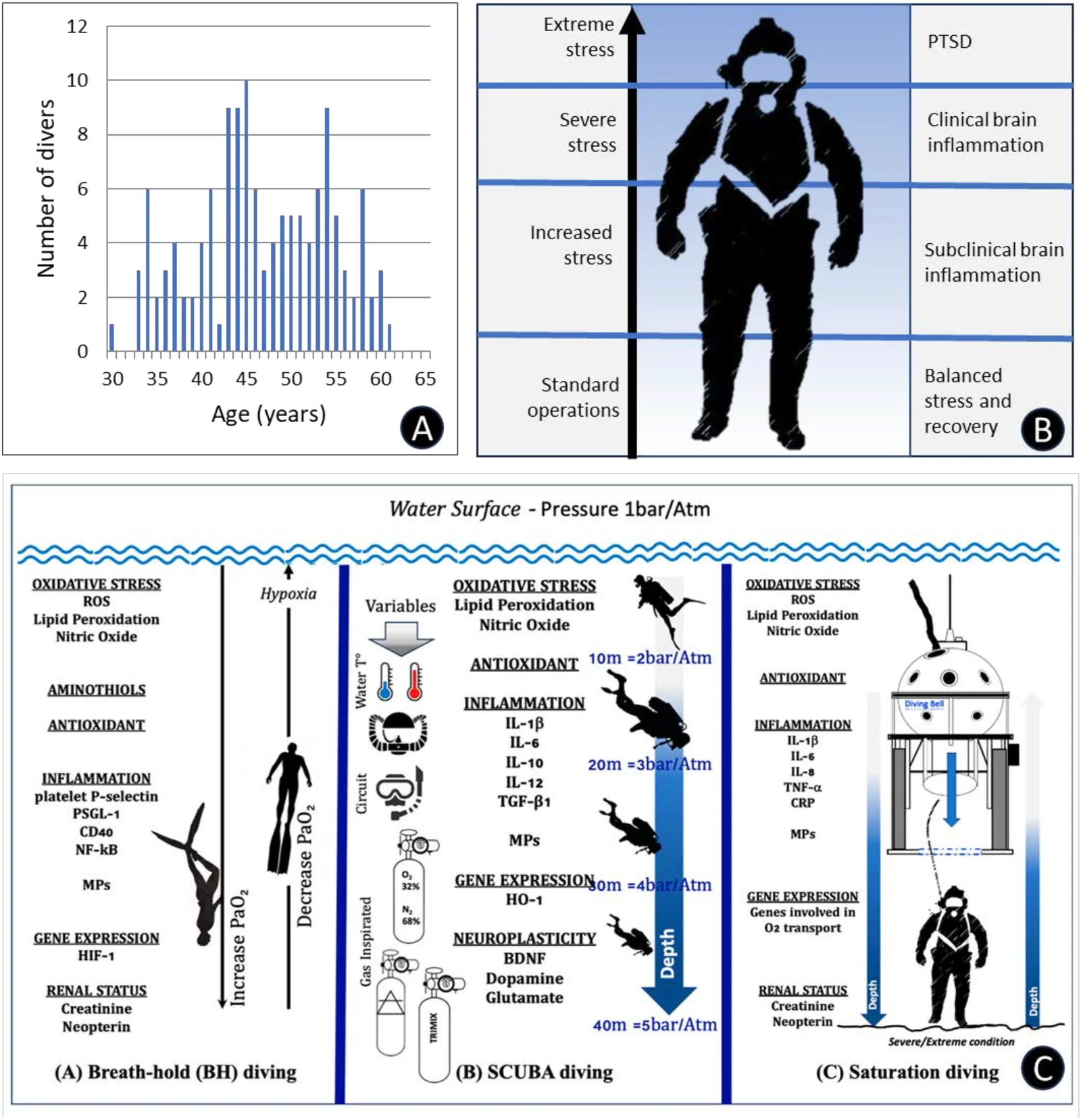
**(A)** Age distribution of saturation divers working on a large diving support vessel operating in the North Sea. **(B)** The long-term health effects of diving seen as a graded response to diving and decompression stress. **(C)** Image from reference (Vezzoli et al., 2024).

### 11.11 Perspectives: neuroplasticity

Neuroplasticity is the nervous system’s capacity for lasting adaptation. Stressful exposures can produce persistent beneficial or adverse changes, including effects on anxiety, sleep, memory, and cognition. Repeated diving exposures may therefore deserve consideration not only as acute decompression events but also as potential cumulative neurological stressors.

Neuroinflammation has been detected after diving by measuring MPs in the glymphatic system of mice after decompression (Thom et al., 2023b; Bhat et al., 2024b). Vezzoli et al. have studied the up- and downregulation of biomarkers in different human underwater activities (Vezzoli et al., 2024):

- Pro-inflammatory cytokines, including interleukins such as IL-6, play a significant role in the initial response to inflammation. IL-6 directly stimulates vascular endothelial cells to produce proinflammatory cytokines.
- Brain-derived neurotrophic factor is a neurotrophic factor that is pivotal in our understanding of the pathophysiology of depression.
- The neuromodulator dopamine influences cognitive, emotional, and motor processes. These are consequently affected in diseases with disturbed dopamine function, such as Parkinson’s syndrome or schizophrenia.
- The decrease in gelsolin is known in acute and chronic inflammatory states. Clinically, reduced gelsolin has been associated with tissue damage or adverse clinical outcomes, particularly in neuroplasticity.

These data suggest that diving can be considered a series of stress and recovery periods. A diver can plan a diving career without worries if the stress and recovery are balanced. When planning more stressful diving activities, such as deep diving, a selection must be made to ensure that the diver can cope with the associated stresses (HPNS, dense gas, thermal stress, longer decompression times, etc.). Criteria may include age, prior deep-diving exposure, VO2 max, etc. Mitigation procedures may include shorter dive times, longer post-dive periods, etc (Imbert, 2005).

When a certain inflammatory threshold is exceeded, chronic inflammatory disorders may appear that could lead, in extreme cases, to symptoms suggesting post-traumatic stress disorder (Millar, 2019). This provides a frame to address the long-lasting problem of diving long-term health effects as a diver’s graded response to inflammatory stress (Mcdiarmid et al., 2004) (**Figure 18B**).

**Figure 18C** illustrates the up- and downregulation of biomarkers during different human underwater activities (A) Breath-hold diving. Physiological changes in PaO2 during the descent and ascent of a deep breath-hold dive are one cause of biological changes at the systemic and molecular levels. (B) scuba diving. The response in scuba diving varies with the number of dives, depth, water temperature, type of breathing circuit (open vs. CCR), inspired gases, and exercise. (C) Saturation diving. In the sketch, the working depth corresponds to the maximum depth reached by the divers. All these activities involve changes in oxy-inflammation levels, redox states (aminothiols), gene expression, antioxidants, neuroplasticity markers, and renal status.

## 12 Secondary bubble generation

The cascade of events generated by MP and activated neutrophils can produce gas-containing MP that can be considered second-generation micronuclei prone to producing microbubbles.

### 12.1 Concept: gas-containing MP

Enzyme activity within the MP, such as nitric oxide synthase 2 (NOS2), can form a gas phase within the particles. Yang et al. have shown that annexin V-positive MP can expand up to 1 micron after decompression (Yang et al., 2012). Reversing particle enlargement can abrogate vascular damage (Thom et al., 2013).

Enzyme activity can also generate systemic inflammation:

- The rigidity of the MP F-actin shell allows phosphatidylserine, which is ubiquitously present in the MP membrane, to be recognized by a receptor complex that includes CD36, Toll-like receptor, and the receptor for advanced glycation end-products.
- These proteins are linked to a scaffold protein called the nitric oxide synthase 1 adaptor protein. When the receptors bind to an MP, an increase in membrane colocalization occurs, and NOS2 binds to the complex (Thom et al., 2017; Thom et al., 2023a).
- NOS2 facilitates S-nitrosylation of Src kinases and the cytoskeleton, forming the nucleotide-binding domain leucine-rich repeat (NOD)-like receptor, pyrin-containing 3 (NLRP3) inflammasome.
- The NLRP3 inflammasome is responsible for producing mature interleukin (IL)-1β (Thom et al., 2014; Thom et al., 2017; Thom et al., 2018).

These complex mechanisms suggest that gas-containing MP might constitute another form of bubble nuclei, facilitating microbubble formation from supersaturated gas (**Figure 19A**).

**Figure 19:**
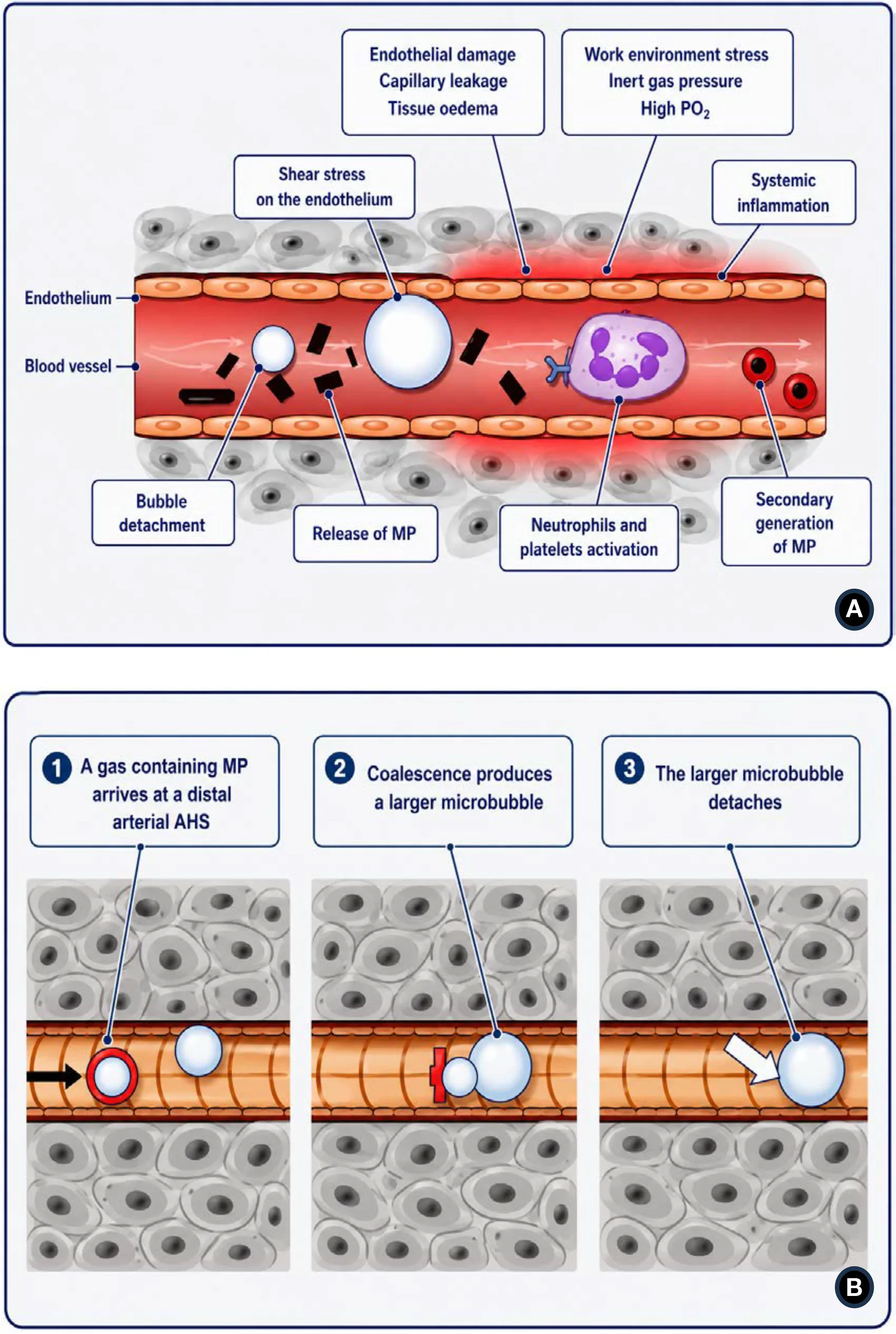
**(A)** Two ways of producing a second generation of MP/MV: inflammatory endothelium insult and neutrophil activation. **(B)** A gas-containing MP activating an AHS and producing a detached microbubble.

### 12.2 Concept: second generation of microbubbles

These gas-containing MP may lose some gas in the alveolar capillaries. Yet the transfer time is short, the exchange surface is reduced, and the MP will continue to cycle in the bloodstream, ready to grow. An AHS may allow these gas-containing MP to merge with the local microbubble by coalescence and expand. This way, the gas-containing MP could participate in the formation of secondary microbubbles, thereby explaining the delayed onset of DCS symptoms (**Figure 19B**).

### 12.3 Implication: AHS depletion versus inflammation during multiday diving

As the initial AHS population can be depleted by decompression, we postulated that multiday diving can be viewed as a sort of diver’s preconditioning that progressively reduces the number of available AHS and, consequently, the number of VGE measured after a dive. In this scheme, progressive exposure to diving is beneficial.

However, we also understand that the beneficial reduction in available AHS and the detrimental cumulative inflammation interact over time during multiday diving. The resulting effect is expected to depend on the pattern of diving days and rest days, as well as the criticality of the dive exposures.

In a technical divers’ monitoring study, VGE measurements were collected during a series of severe dives in the depth range of 100 msw (Balestra et al., 2024b). The VGE number per heartbeat decreased during the first five days (pre-conditioning effect) and then increased during the sixth day, which was also associated with the more severe dive (**Figure 20A**). The monitoring protocol involved several other measurements, including biomarker dosages. The multiple factor analysis suggests that the detrimental impact of inflammation prevailed over the beneficial effect of AHS population depletion after around one week of continuous diving.

**Figure 20:**
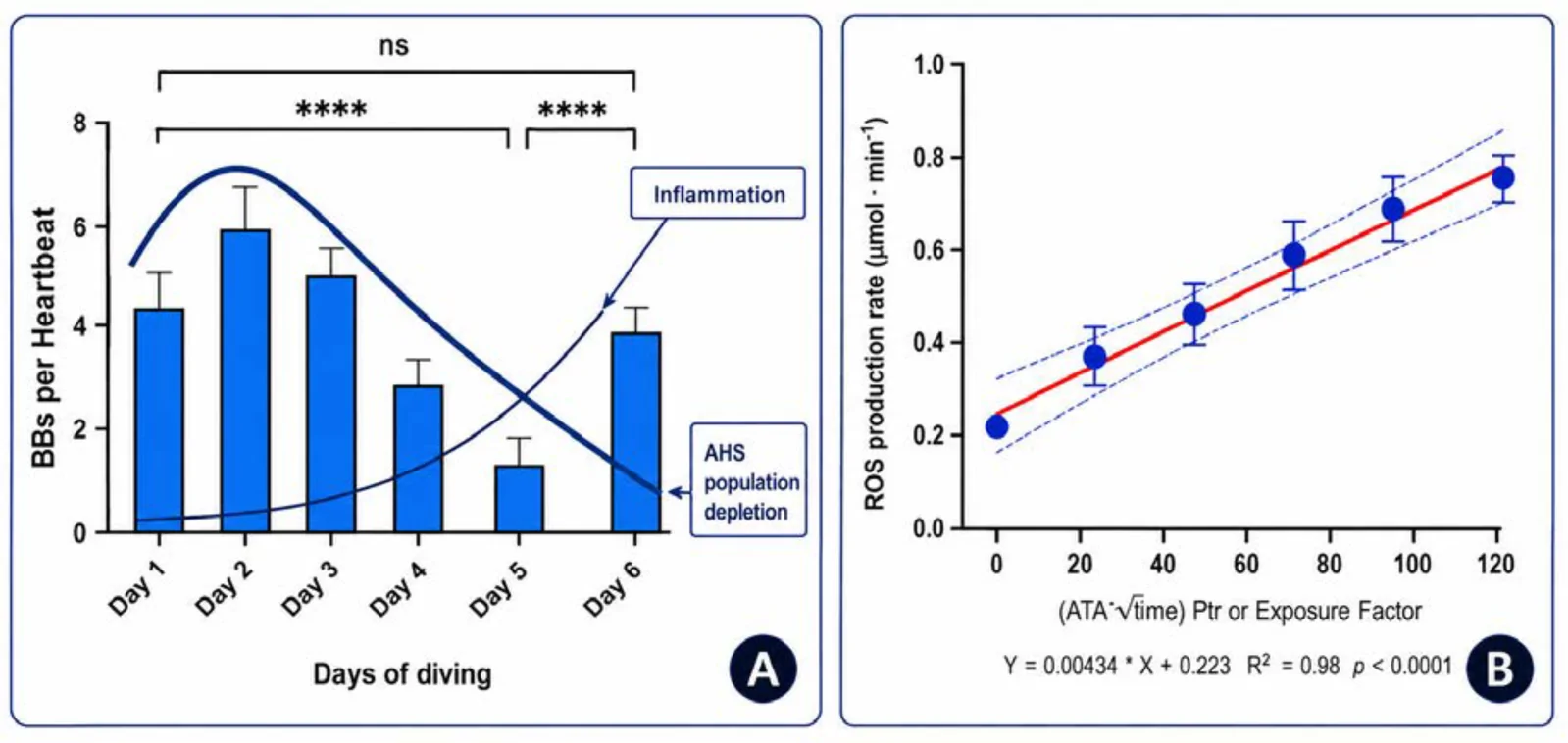
**(A)** Evolution of the bubble signal in CCR divers after a series of six 100 msw dives performed over six consecutive days. **(B)** Relationship established with the measured ROS production along the dives as a function of the cumulative inflammation dose, expressed as the cumulative addition of the Prt index of each dive. **** = p<0,0001.

### 12.4 Perspective: management of bounce diving inflammation dose

The challenge remains to estimate this inflammation level from documented cases where inflammation has been measured.

Referring to the technical divers’ monitoring session already mentioned (Balestra et al., 2024b), the daily Prt indexes defined after each dive have been added throughout the week as a tentative definition of the cumulative inflammation dose. **Figure 20B** shows a significant regression between the cumulated Prt Index and the measured ROS production in the divers. This enables modeling inflammation by estimating dive-exposure severity based on dive depth and bottom time (assuming one dive per day).

### 12.5 Perspective: management of multiday diving

Multiday diving differs from repetitive diving in that the surface interval between dives is at least 12 hours, and the dive pattern repeats over days. The two effects, AHS depletion and cumulative inflammation, are expected to act simultaneously.

For a short diving period, like a week, AHS depletion will remain beneficial. This could explain why recreational divers of any age, experience, and fitness can spend a week on a liveaboard vessel without any dramatic episode of DCS in the Maldives or similar diving paradises. These divers perform at least three dives a day, sometimes with an additional night dive. Yet these dives remain shallow (typically 30 msw, 24 msw, and 18 msw), and their bottom time is limited to the no-decompression envelope (Gillian, 1991).

For diving periods longer than a week, inflammation may prevail if efficient recovery measures are not implemented. The accumulation of primary and secondary circulating MP results in persistent endothelial dysfunction and an increased susceptibility to DCI.

Diving companies include policies in their manuals defining interruptions in air-diving operations, generally one day off for every five consecutive days of diving. This empirical approach seems to work, at least in the North Sea, where the British HSE safety memorandum limits the bottom times for air diving (UK Health & Safety Executive, 1998).

## 13 Genetics

The most consistent clinical observation in the field is that, under identical exposures, some divers and some animals develop severe DCS while others remain entirely unaffected (Balestra et al., 2019; Mitchell et al., 2022; Mitchell, 2024).

Over the last decade, the demonstration that resistance to DCS is heritable, together with the application of multi-omics platforms to selectively bred animals, has begun to define the molecular basis of that host contribution (Lautridou et al., 2017). This chapter summarizes the genetic, transcriptomic, metabolic, and physiological evidence for individual susceptibility, then develops the link between host genotype and the physical site at which bubbles actually nucleate: the surfactant-coated active hydrophobic spot on the vascular endothelium.

### 13.1 Epidemiological evidence of individual variation

Several independent lines of human evidence document that susceptibility is unevenly distributed.

A right-to-left shunt, most often a PFO, is the best-characterized structural risk factor. It is present in roughly a quarter to a third of the population, shows familial clustering, and is associated with a clearly increased risk of DCS, particularly of cerebral, spinal, inner-ear, and cutaneous forms (Germonpre et al., 2021; Lee et al., 2023).

Sex also modulates susceptibility through physiological and possibly hormonal mechanisms, as illustrated by latent-class analyses of symptom patterns in female breath-hold divers (Kim and Han, 2022). Large observational datasets of recreational and occupational divers confirm that, after accounting for exposure, residual person-to-person variation in risk remains substantial (Tournoy et al., 2024). Together, these observations establish individual susceptibility as a real and quantifiable phenomenon, but human cohorts cannot easily separate genetic from environmental determinants, which is the principal value of the animal model described next.

A complementary line of evidence comes from candidate-gene studies in humans: in a cohort of experienced breath-hold divers, polymorphisms in endothelial nitric-oxide synthase (eNOS G894T and eNOS T786C) and in the angiotensin-converting enzyme (ACE) insertion/deletion locus were significantly associated with breath-hold diving-induced hemoptysis, a form of pulmonary edema, the high-risk eNOS “TT” genotypes carrying a several-fold higher prevalence, and the ACE “II” genotype appearing entirely protective (Cialoni et al., 2015).

### 13.2 Concept: the decompression sickness-resistant rat model

The single most informative experimental tool in this field is the line of DCS-resistant rats obtained by breeding from animals that remained asymptomatic after standardized hyperbaric exposures over successive generations until a stable resistant strain was fixed (Lautridou et al., 2017; Lautridou et al., 2020). The success of this selection demonstrates that resistance is, at least in part, heritable (Lautridou et al., 2017). The asymmetric rise in resistance in females before males suggested a possible X-linked contribution, a hypothesis supported by an X-chromosome single-nucleotide polymorphism reported in the selected line. Resistant animals also differ from standard Wistar rats in vascular physiology: Dugrenot et al. (2022) found a tendency toward lower vascular tone in animals that had never undergone hyperbaric exposure. Importantly, this study did not use injected bubbles. Later molecular studies examined baseline gene-expression phenotypes rather than bubble injection, including antioxidant and mitochondrial-biogenesis pathways (Dugrenot et al., 2026). These observations should therefore be interpreted as characteristics associated with the resistant phenotype, not as responses to experimentally injected bubbles.

### 13.3 Concept: genetic and molecular determinants of susceptibility

#### 13.3.1 The MYD88–NFKB1 innate-immune axis

Working with the resistant line, Vallée et al. performed the first systematic functional analysis of single-nucleotide polymorphisms in MYD88 and NFKB1, two central nodes of innate-immune signaling (Vallée et al., 2023). MYD88 is the principal adaptor of Toll-like receptor and interleukin-1 receptor signaling. NFKB1 encodes a core transcription factor of the NF-κB pathway. Together, they govern the acute inflammatory response to tissue injury.

The data suggest that bubble-induced release of damage-associated molecular patterns activates this cascade, and that resistant animals carry variants that restrain the resulting pro-inflammatory amplification (Vallée et al., 2023). This, as already described, reframes part of DCS as an immune-inflammatory, host-modulated disorder rather than a purely mechanical injury.

#### 13.3.2 Transthyretin variation

A complementary candidate emerged from Orsat et al. (2026), who reported a synonymous single-nucleotide polymorphism in transthyretin (TTR) associated with the DCS-resistant phenotype, together with lower hepatic TTR mRNA expression in resistant animals. Because this was a small pilot study, the finding should be regarded as a candidate association requiring replication rather than a demonstrated causal mechanism.

As the principal transporter of thyroxine, TTR could plausibly connect basal metabolic state, inflammation and protein homeostasis to DCS susceptibility, but the proposed link to pre-existing gas micronuclei remains hypothetical (Orsat et al., 2026).

#### 13.3.3 miRNA networks and the acute transcriptome

Beyond DNA-level variants, Dugrenot et al. (2024) used a crossed circulating-miRnome/hepatic-transcriptome approach to identify regulatory networks distinguishing resistant from standard rats. The study implicated inflammatory, circadian, signaling, phagocytic and apoptotic pathways, supporting a host-regulation component without establishing that any single pathway is causal.

In humans, Magri et al. characterized the acute peripheral blood transcriptome after DCS and found activated innate immunity with suppressed adaptive immunity in the acute phase, most differences resolving on recovery (Magri et al., 2021).

A distinct experiment by Orsat et al. (2026) directly addressed the response to bubbles independently of hyperbaric exposure. Standard Wistar and DCS-resistant rats received intravenous air microbubbles. Resistant rats showed lower pulmonary wet-to-dry ratios and a different hepatic transcriptional response involving tissue factor, tissue-factor-pathway inhibitor, heat shock protein 70, heat shock protein 27, and early growth response protein 1. This is the study in this literature set that specifically used injected air microbubbles; it should not be attributed to Dugrenot et al. (2026).

The Orsat et al. (2026) findings are consistent with better tolerance of bubble-induced thrombo-inflammatory and cellular stress in the resistant line. They complement, but are experimentally distinct from, Dugrenot et al. (2026), who studied baseline liver and lung expression of antioxidant-defense and mitochondrial-biogenesis genes in resistant versus standard rats rather than responses to injected bubbles. In experienced human divers, Eftedal et al. (2013) showed that even asymptomatic scuba diving alters the peripheral-blood transcriptome toward innate-immune, inflammatory and apoptotic programs, including tumor necrosis factor receptor 1 (TNFR1)–related caspase signaling.

### 13.4 Concept: metabolic, microbial, and lipid determinants

Metabolomic studies of the resistant line reveal a consistent rerouting of host metabolism. The cecal metabolome of resistant or unscathed animals carries a recognizable fingerprint, including a hormonal reshuffle and modified bile-acid profiles, which distinguishes them from susceptible animals and tracks with outcome (de Maistre et al., 2020; Vallée et al., 2021; Desruelle et al., 2022).

The gut microbiota participates causally rather than incidentally: manipulations that alter the microbiota, such as CO₂ pre-breathing, change DCS risk in rodent models (Daubresse et al., 2024). Lipid biology is a particularly important thread because inert-gas solubility is far higher in lipid than in aqueous phases. The fatty-acid composition and molecular structure of adipose triacylglycerols govern nitrogen solubility and thus local gas loading (Gabler-Smith et al., 2020), while individual differences in pulmonary gas transfer add a further physiological modifier of risk. The recurring observation that resistant animals modify bile-acid and lipid handling marks the point at which the metabolic layer begins to connect to the physical event, because the same lipid economy controls the supply of the surfactant that stabilizes bubble nucleation sites.

### 13.5 Concept: alveolar fluid clearance

A further host interface emerges at the alveolar epithelium. In the intravenous air-microbubble experiment, DCS-resistant rats accumulated less pulmonary fluid than standard Wistar rats, as indicated by a lower lung wet-to-dry ratio (Orsat et al., 2026). This observation is compatible with reduced vascular leakage and/or more effective pulmonary fluid handling, but the experiment did not directly measure alveolar fluid-clearance mechanisms.

Human evidence for genetically modulated pulmonary susceptibility comes instead from breath-hold divers: eNOS (G894T and T786C) and ACE (I/D) variants were associated with diving-induced pulmonary edema/hemoptysis (Cialoni et al., 2015). This human observation should not be cited to Dugrenot et al. (2026).

The molecular machinery of that clearance is well defined: active Na⁺ reabsorption across distal lung epithelial cells, driven by apical epithelial sodium channels (ENaC) together with the basolateral Na⁺–K⁺-ATPase, limits the degree of alveolar edema in acute lung injury and cardiogenic edema (Matalon et al., 2015). Because ENaC activity is strongly downregulated by reactive oxygen and nitrogen species (Matalon et al., 2015), the antioxidant economy that distinguishes resistant tissues would be expected to modulate epithelial fluid clearance, linking the favorable oxidative phenotype of resistant animals to their lower pulmonary fluid load.

The interface also connects to coagulation: plasmin both regulates ENaC and lies at the crossroads of fibrinolysis and inflammation, and elevated plasminogen has been proposed as a common host susceptibility factor in epithelial-vascular injury (Ji et al., 2020).

The coagulation link is supported specifically by Orsat et al. (2026): after intravenous air-microbubble exposure, resistant rats showed a distinctive transcriptional pattern involving tissue factor and tissue-factor-pathway inhibitor. Alveolar fluid clearance remains a plausible host layer between bubble load and phenotype, but ENaC involvement in DCS resistance has not yet been demonstrated directly.

Direct human evidence that this epithelial-vascular interface is under genetic control comes from breath-hold divers, in whom eNOS (G894T, T786C) and ACE (I/D) variants—loci that govern nitric-oxide availability and peripheral vascular tone—strongly predict susceptibility to diving-induced pulmonary edema and hemoptysis (Cialoni et al., 2015). This reinforces the view that heritable control of endothelial and fluid-clearance function shapes the pulmonary response to bubbles.

### 13.6 Implications: from genotype to the bubble, surfactant (DPPC) at active hydrophobic spots

Genetics of DCS is only mechanistically complete if it can be connected to the place where bubbles actually begin. Experimental and theoretical work presented previously indicates that decompression bubbles form at specific sites (AHS): discrete hydrophobic patches on the luminal surface of blood vessels at which pre-existing nanobubbles are stabilized and from which gas micronuclei grow on decompression (Arieli, 2017; Arieli and Marmur, 2017).

The same principle extends to extravascular hydrophobic surfaces implicated in spinal, articular, and osteonecrotic disease (Arieli, 2018b). Because the AHS is the physical origin of the gas phase, any host factor that affects the number, distribution, or activity of these AHS will change the bubble production and act on the “physical” aspect of decompression rather than only on the inflammatory response.

Surfactant DPPC is proposed as the molecular link. DPPC and related dipalmitoyl phosphatidylcholines are strongly surface-active and are thought to coat and stabilize the hydrophobic luminal spots, favoring nanobubble persistence and bubble nucleation (Arieli, 2021a).

Comparative physiology supports a protective value in reducing this surfactant: the DPPC content of deep diving mammals falls as a function of maximal diving depth, as if they had evolved to limit the very sites at which bubbles would otherwise form (Arieli, 2021a). This leads to a hypothesis for the resistant rat: if resistant animals carry less DPPC at their vascular AHS—plausibly through the reduced plasma carrying capacity implied by their altered bile-acid and lipid metabolism (Desruelle et al., 2022)—they should nucleate fewer or less stable bubbles for the same exposure, and the level of DPPC at the vessel wall would become a direct, measurable determinant of both bubbling and DCS severity.

This hypothesis closes the loop with the genetic data:

- The metabolic and bile-acid phenotype reported in resistant or unscathed animals (de Maistre et al., 2020; Vallée et al., 2021; Desruelle et al., 2022) could influence the plasma lipid environment and, hypothetically, the supply of DPPC,
- The supply of DPPC would set the density and stability of AHS,
- The AHS density would set the bubble load presented to the MYD88–NFKB1 inflammatory machinery (Vallée et al., 2023) and to the coagulatory and cellular-stress responses that the Orsat et al. (2026) intravenous air-microbubble experiment found to differ between resistant and standard rats.

### 13.7 Proposed model: linking host genotype and DCS

In this reading, host genotype influences DCS at two distinct stages by tuning:

- How many bubbles form on the surfactant deposit at the AHS,
- How damaging the bubbles are, through their immune, coagulatory, and antioxidant responses.

Direct quantification of vascular DPPC in resistant versus control animals is therefore the natural next experimental step, and would convert the surfactant bridge from a plausible model into a measured mechanism (Arieli, 2026).

The classical model treats DCS risk as a function of the physical bubble burden (B), set by the pressure–time profile and inert-gas supersaturation (left). Evidence from selectively bred DCS-resistant rats, multi-omics studies, and human candidate-gene and transcriptomic data adds a host susceptibility term (H), organized as four interacting layers (center).

- Layer 1: Genetic variation in innate-immunity genes MYD88 and NFKB1, in TTR and gelsolin (GSN), and—from human breath-hold divers—in the vascular-tone genes endothelial nitric-oxide synthase (eNOS G894T, T786C) and ACE I/D, whose variants predict susceptibility to diving-induced pulmonary edema and hemoptysis (the ACE II genotype appearing protective).
- Layer 2: Molecular and epigenetic regulation through miRNA networks and the peripheral-blood transcriptome—in which even asymptomatic scuba diving activates innate-immune, inflammatory and apoptotic programs (notably the TNFR1 caspase axis), with a stationary, potentially persistent between-dive signature in experienced divers—together with plasma gelsolin, MPs/extracellular vesicles, circulating tau and mitochondrial DNA.
- Layer 3: Metabolic and microbial state, including hepatic bile-acid remodeling, the gut microbiota and lipid metabolism relevant to inert-gas solubility and to the lipid environment from which vascular surfactant could be supplied.
- Layer 4: Physiological phenotype, including vascular surfactant/DPPC at active hydrophobic spots, pulmonary fluid handling, antioxidant and mitochondrial capacity, vascular tone, and right-to-left shunting. Some components are established physiological modifiers, whereas the proposed connection between host lipid biology, DPPC deposition and AHS activity remains a Level-3 mechanistic hypothesis.

### 13.8 Perspectives: candidate biomarkers and effector molecules

Several circulating molecules link the framework to measurable risk.

pGSN is currently the most translationally attractive: it constrains the release of interleukin-1β-bearing MPs after pressure exposure (Bhopale et al., 2021), and recombinant human pGSN suppresses persistent neuroinflammation and restores hippocampal neurogenesis in murine DCS, identifying it as a candidate adjuvant therapy (Kumar et al., 2024).

MPs and extracellular vesicles are themselves both effectors and biomarkers of decompression stress, are elevated in divers, and drive vascular and neuro-inflammation in part through the scavenger receptor CD36 (Thom et al., 2019; Thom et al., 2023a; Bhat et al., 2025). Peripheral blood tau rises after diving and may report subclinical neural injury, plausibly related to distal arterial bubbles (Arieli, 2022; Rosén et al., 2022), while circulating mitochondrial DNA behaves as a damage-associated signal that falls during uneventful diving but rises with injury (Blatteau et al., 2018). None of these markers is yet validated for routine clinical use, but collectively they provide the tools needed to quantify individual risk.

### 13.9 Perspectives: an integrated host-bubble framework

These observations can be organized as a host-modified extension of the classical model (**Figure 21**). Where the classical model treats DCS risk as a function of bubble load alone, the evidence reviewed here adds a host term with interacting layers: genetic variation (MYD88–NFKB1, TTR), epigenetic and molecular regulation (miRNA networks, pGSN, MPs), metabolic and microbial state (bile acids, lipid handling, gut microbiota), and the resulting physiological phenotype.

**Figure 21:**
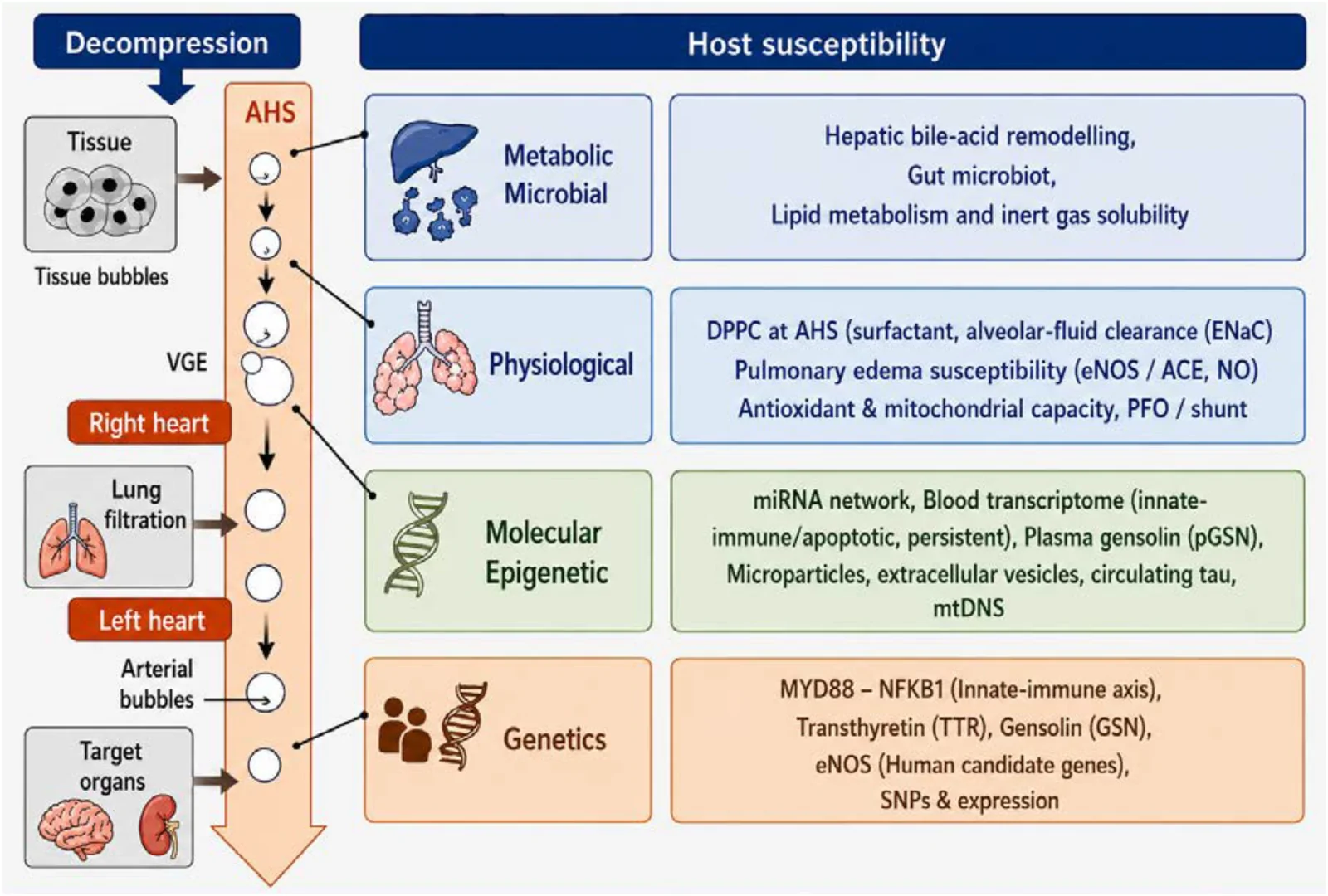
From host genotype to bubble nucleation: the surfactant (DPPC) bridge. Genetic susceptibility to DCS, integrated as a host-modified bubble model. Solid arrows denote the principal causal chain; dashed arrows denote host modulating influences.

The surfactant bridge adds an important refinement: through DPPC at active hydrophobic spots, the host also acts on the bubble signal, so the genetic and physical models are not alternatives but two ends of a single causal chain that runs from genotype, through lipid metabolism and vascular surfactant, to bubble nucleation, and finally to a host-determined inflammatory and coagulatory response (Arieli, 2017; Arieli, 2021c; Vallée et al., 2023; Orsat et al., 2026).

### 13.10 Perspectives: precision prevention and future directions

A host-aware view of DCS suggests procedures interventions matched to individual susceptibility. Established measures such as pre-dive hydration and different preconditioning such as exercise or other (Gempp et al., 2009a; Balestra et al., 2016b; Germonpre and Balestra, 2017; Lambrechts et al., 2022), are known to reduce bubble formation and could be prioritized in higher-risk individuals (Wang et al., 2020; Lambrechts et al., 2022), while structural management such as PFO evaluation addresses a defined anatomical risk factor (Germonpre et al., 2021; Vallée et al., 2023).

Emerging pharmacological targets, for example, agents that limit oxidative stress (Balestra et al., 2016a; Daubresse et al., 2026) or restore gelsolin reserves (Bhat et al., 2024a), map directly onto specific layers of the framework.

Progress will require adequately powered human genomic studies, careful phenotype stratification, attention to species differences, and analysis of gene–environment interactions. International collaborative databases linking exposure profiles, DCS phenotypes, biomarkers, and genomic data will be essential for translating animal findings into human risk prediction.

Genetic susceptibility shifts DCS from a purely physico-chemical description toward a host-modulated framework. Heritable resistance in rats, immune and metabolic differences, candidate human variants, and physiological interfaces support a model in which host biology modifies the response to a given decompression exposure. These findings do not replace bubble physics; they identify biological factors that may alter bubble formation, tolerance, and downstream injury.

## 14 Pathways to DCS

We have identified several bubble scenarios that constitute pathways to DCS. Each pathway has its own time scale.

In the first scenario, the microbubble starts its journey from a venous AHS, detaches from its substrate, travels through the venous vessels as a VGE, and becomes a shunted arterial microbubble within a dozen minutes. If the target organ is still supersaturated, the bubble can grow to a critical size. This explains why skin rash and vestibular symptoms are reported within 20–30 min after the end of the decompression (Mitchell et al., 2022). The shunted arterial bubble will have no direct effect if the target organ is no longer supersaturated. We postulate that this scenario is the first to be triggered by the decompression.

The second scenario concerns spinal neurological symptoms. In a study involving 1,070 cases of neurological DCS, Francis et al. reported that 50% became symptomatic within 10 min and only 15% after 1h (Francis et al., 1988). We postulate that this scenario is the second to interfere with the decompression ascent.

The last scenario concerns Type I DCS, and delays are measured in hours.

These scenarios are further exacerbated by MP production and systemic inflammation. We understand that endothelial damage makes divers more susceptible to DCS, but we lack data to estimate when this inflammatory process begins. Brett et al. have exposed divers to 60 min@18 msw and 35 min@30 msw on air. Results demonstrate that in humans, exposures to high gas pressures trigger production of MP containing elevated concentrations of IL-1β and neutrophil activation, which are still persistent 2h after the end of the dive (Brett et al., 2019). We believe the process could go on for hours, with prolonged saturation decompression, repetitive dives, and multiday dives.

### 14.1 Model for the pathogenesis of skin DCS

Cutaneous manifestations of DCI should not be treated as a single entity. Pruritus without visible change, common after dry chamber exposures and usually attributed to inert gas entering the skin directly, is benign and self-limiting and has no established relation to systemic DCI.

Localized swelling with an orange-peel appearance suggests lymphatic involvement and follows a different time course. A non-specific erythematous rash is reported after many exposures, and its relation to decompression is often uncertain.

Hartig et al. explained that the term Cutis marmorata encompasses two clinically distinct manifestations: Livedo reticular and Livedo racemosa (Hartig et al., 2020). Livedo racemosa is associated with a thromboembolic occlusion of arterial vessels and fits into the scenario of shunted arterial microbubbles. Authors have reported the prevalence of right-to-left cardiac shunts in cutis marmorata (Wilmshurst et al., 2001). Garcia et al. used ultrasound echography on four divers exhibiting Cutis marmorata and observed rapidly moving bubbles similar to arterial bubbles beneath the site of the skin lesions (**Figure 22A**). All the divers were later confirmed to have a large PFO (García and Mitchell, 2020).

**Figure 22:**
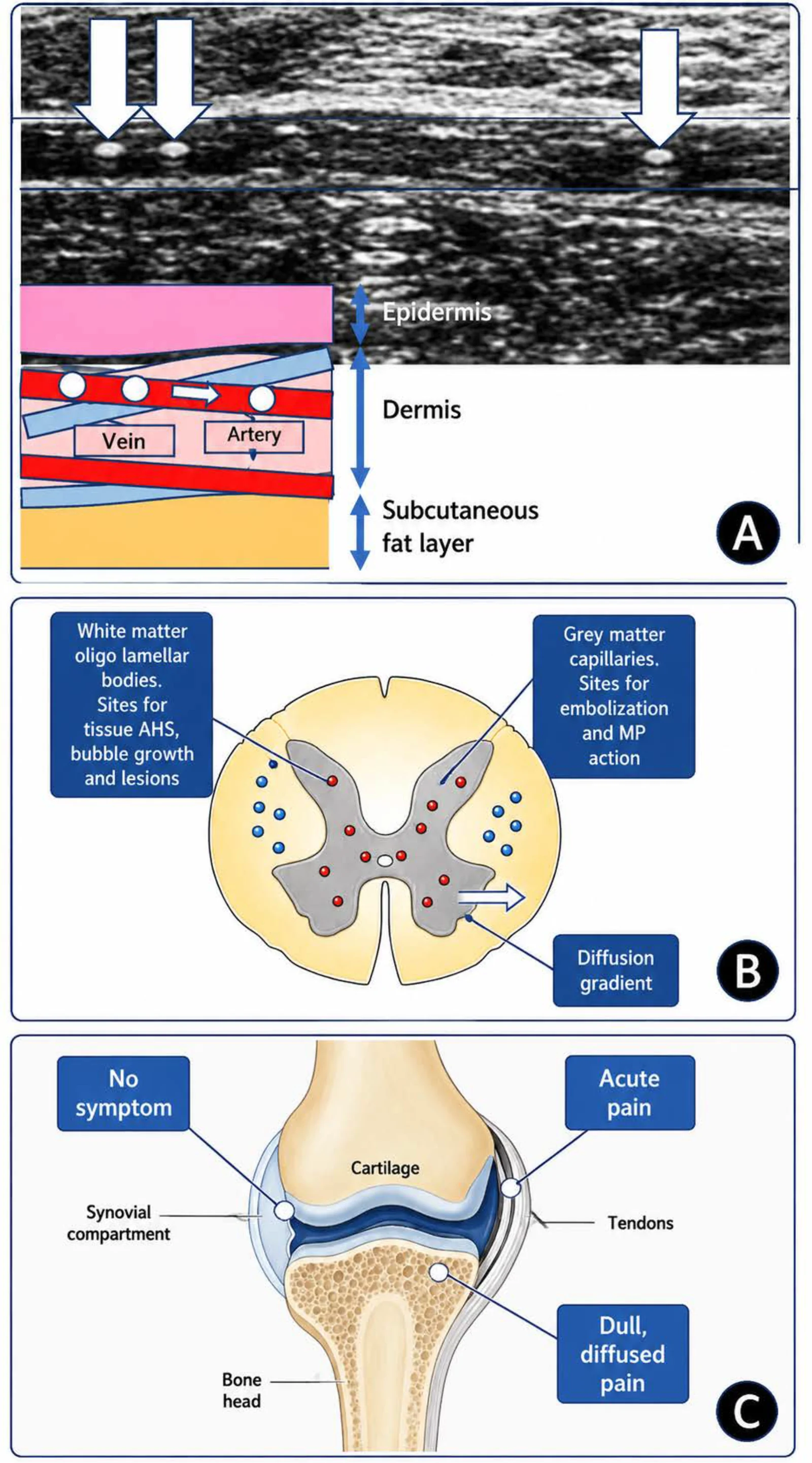
**(A)** Still capture of a video posted by Garcia and Mitchell, showing moving bubbles under a cutis marmorata lesion. The rectangle frames the blood vessel; the arrows point to the moving bubbles. Accessed 01/03/2024 from https://www.dhmjournal.com/index.php?option=com_content&amp;view=article&amp; id=70. The figure aside describes the location of the bubbles in the small arteries. **(B)** Combination of two scenarios, shunted arterial microbubbles (or MP) and tissue AHS, for the occurrence of lesions in the white matter during spinal decompression. **(C)** Three possible bubble locations for a pain-only DCS.

The second hypothesis proposes autochthonous bubbles as the etiology for skin lesions. In a study using swine, the lesion appeared after severe decompression but without heart shunts. Skin thickness from the squamous keratin to the dermis increased by 10% in the affected area. This experiment fits into the assumption of pre-existing gas nuclei and the tissue bubble scenario, given the presence of lamellar hydrophobic bodies in the granular layer of the skin (Arieli, 2018a). It would best correspond to Livedo racemosa, which involves reduced blood supply, like in cold exposure (Hartig et al., 2020).

A recent paper notes that the brainstem controls skin microcirculation. The authors, arguing that no gas has been detected in the skin during the occurrence of Cutis marmorata, propose that the skin symptoms result from a tiny arterial embolization of the brainstem due to shunted arterial bubbles (Germonpre et al., 2015; Kemper et al., 2015). This hypothesis is compatible with our arterial bubble scenario if the brainstem becomes the target organ during the decompression. Disruption of brainstem vasomotor regulatory neurons could cause inappropriate vasodilation and vasoconstriction in skin areas innervated by the affected neurons. This would elicit an inflammatory response in the skin, characterized by activation of white blood cells, activation of inflammatory biochemical pathways, fluid extravasation, and stimulation of sensitive nerve fibers. However, this hypothesis suggests that skin rash should be associated with cerebral symptoms, which is not always the case (Wilmshurst, 2015).

### 14.2 Model for the pathogenesis of inner ear DCS

Inner ear symptoms are common in recreational diving. They have also been reported in commercial bounce diving, whether on air or heliox, and after extended saturation excursion dives. Manifestations include dizziness, nausea, vertigo, vomiting, loss of balance, tinnitus, and ear pain.

Inner ear DCS is characterized by symptoms that:

- Occur rapidly, generally 15–30 min after decompression (Mitchell and Doolette, 2015).
- Are associated with skin and neurological disorders in 15% of cases (Gempp and Louge, 2013).
- Are almost always linked to a large right-to-left shunt (around 80% of the cases) (Gempp and Louge, 2013; Mitchell and Doolette, 2015).
- Are more common on the right side due to possible vascular anatomical selectivity (Gempp and Louge, 2013).

The hyperbaric center in Toulon, a region of France where intensive recreational diving occurs, has implemented a systematic procedure for detecting PFO in divers arriving at the chamber. According to their findings, 95% of divers presenting with vestibular symptoms had a right-to-left shunt (Gempp et al., 2017).

Inner ear DCS is widely recognized as being caused by shunted arterial bubbles. The inner ear has very low vestibular perfusion and longitudinal terminal arterioles without collateral supply (Arieli, 2019b). Recently, Mitchell and Doolette have proposed a complete model of the inner ear symptoms observed after saturation excursions based on the prolonged survival time of arterial bubbles (Doolette and Mitchell, 2022). They used the Srinivasan/Gerth model, which considers three regions (a well-stirred tissue, a bubble, and a shell of liquid surrounding the bubble across which gas diffuses) to simulate an actual case occurring after a saturation excursion dive at 250 msw (Srinivasan et al., 2000). They found that the symptom onset time matched the maximum supersaturation in brain tissue (35 min). They then computed the survival time of a 20-micron shunted arterial microbubble and found it consistent with the transit time to the inner ear.

Dizziness and vertigo symptoms could be associated with arterial bubbles delivered to the brain rather than the inner ear. The mechanism would be the same, although Mitchell et al. explained that the inert gas washout in the inner ear is slower than in the brain, making it more vulnerable (Mitchell and Doolette, 2015).

We still include the possible involvement of distal arterial AHS because of the occurrence of inner ear DCS during isobaric counter-diffusion (Lambertsen and Idicula, 1975; Hills, 1981), as commented by Mitchell et al (Mitchell and Doolette, 2015). Also, the increased occurrence of inner ear DCS with left/right shunt could be explained by the coalescence of the shunted bubbles with distal arterial stationary bubbles (Arieli, 2023).

### 14.3 Model for the pathogenesis of cerebral DCS

Central neurological DCS affects brain functions and is associated with signs such as personality changes, confusion, and problems with speech, hearing, vision, and balance. Paraesthesia may also be a symptom of central DCS.

The cerebral DCS cases appear rapidly after the dive, and Francis showed that the latency is around 10 min (Francis et al., 1988).

The relationship between brain DCS and the presence of a right-to-left shunt has been well-established (Cantais et al., 2003; Peppas et al., 2023). The logic is to apply the same scenario to cerebral DCS as to inner-ear DCS, that is, shunted arterial microbubbles or distal arterial AHS.

However, the brain is a high-oxygen consumer and has a large, well-distributed blood supply. Multiple adjacent vessels could provide redundancy if a bubble blocks a capillary. Brain capillaries are also smaller in diameter than those in other tissues. Shunted arterial microbubbles act on the vessel endothelium and affect blood–brain barrier permeability (Hills and James, 1991). Because the brain is particularly susceptible to oxidative stress, ROS and RNS can induce drastic changes in cerebral vascular tone (Salvagno et al., 2024). When the blood–brain barrier is breached, plasma proteins leak into brain tissue and trigger inflammation by activating the complement pathway (James, 2014). A similar effect is expected from MP that induce endothelial dysfunction and neutrophil activation (Thom et al., 2012). This change could explain increased glial-based lymphatic function in an animal model of DCS (Thom et al., 2023a).

Magnetic resonance imaging (MRI) can detect small areas of inflammation with increased water content, known as unidentified bright objects. These lesion-like signals are common in the normal population but also appear in a diver after a neurological DCS. Brain lesions in the diver have been associated with the presence of a right-to-left shunt (Gerriets et al., 2003). The presence of brain lesions is particularly worrying, as they were detected in divers who had not reported any DCS symptoms (Gerriets et al., 2003). Knaut et al. studied a population of 87 sport divers and found multiple silent brain lesions in 11, related to the presence of a right-to-left shunt (Knauth et al., 1997).

However, Balestra & Germonpré studied a randomized sample of recreational divers with extensive diving experience and no history or symptoms of DCS, and they found a higher prevalence of cerebral MRI abnormalities in divers with a PFO than in those without a right-to-left shunt. Both groups of divers showed a distinct and significant deterioration in neuropsychometric performance on test items measuring working memory and visual-spatial performance. There was no relationship between PFO, diving experience, age, and neuropsychometric findings (Balestra and Germonpre, 2016).

Arieli & Marmur suggested that these white matter MRI lesions could result from distal arterial bubbles (Arieli and Marmur, 2017). The low perfusion in white matter and the increase in these lesions with diving experience support their suggestion.

### 14.4 Model for the pathogenesis of spinal DCS

Spinal DCS affects the medullar tissue and causes severe neurological symptoms. The signs include weakness, paleness, difficulty standing up, paraesthesia, paralysis, bladder problems, and typical girdle pain.

Spinal DCS is often encountered in air diving, and in mixed gas surface decompression, but not in heliox saturation diving (Imbert and Bontoux, 1988). The hyperbaric center in Genova published that spinal DCS represents 55% of the cases they treated (Thaler et al., 2020).

Spinal DCS symptoms occur within one hour after surfacing in air diving, with a longer latency than for cerebral DCS, which suggests a slightly different mechanism (Francis, 1998).

For decades, the diving community was influenced by Hallenbeck, who explained that bubbles form locally, block the venous return, and cause blood stagnation, the so-called “venous damping” effect (Elliott et al., 1974). In animal experiments involving rats exposed to severe decompression, bubbles have been seen in the veins of the spinal cord. Still, it is difficult to determine whether they were a cause of the symptoms or a consequence of the decompression (Alvarado et al., 2024). It is admitted that:

- The absence of non-return valves in the peridural venous plexus causes the venous blood to flow against gravitational forces (in the upright position). It induces slower venous outflow than in other body systems.
- The presence of VGE, as in suboptimal decompression, increases the central venous pressure (retrograde from the pulmonary artery). This raises the possibility of shunting through a PFO. It also increases pressure in the azygos veins and slows venous return from the spinal cord.
- VGE that pool in the venous plexuses of the spinal cord, are likely to trigger coagulation effects, thus further slowing venous outflow.

All these effects modify local blood flow and gas exchange and increase autochthonous bubble formation.

Francis proposed a first mechanism for spinal cord injury involving two scenarios: arterial bubble embolization and autochthonous bubbles (Francis, 1998). He noted the multiple lesion sites detected on MRI. Togawa et al. observed these lesions and explained that neurological dissociation between sensory impairment and motor dysfunction occurs frequently in patients with spinal DCS (Togawa et al., 2006).

A second mechanism could involve shunted arterial microbubbles causing ischemia and small hemorrhages (Wilmshurst and Bryson, 2000). In the upper part of the spinal cord, blood is supplied from the subclavian arteries and the Adamkievicz arteries, and their anatomy is compatible with the survival distance of an arterial microbubble. Gempp et al. confirmed a higher risk of spinal cord symptoms in scuba divers with a large right-to-left cardiac shunt (Gempp and Blatteau, 2009; Gempp et al., 2009b).

A final mechanism could involve extravascular gas bubbles. Butler & Hills observed a higher surfactant concentration in the spinal cord than in the cerebrum, and the myelin of the white matter contains phospholipid lamellar bodies (Hills, 1994). These bodies could serve as tissue AHS for nanobubbles and maintain a population of pre-existing nanobubbles. Also, grey matter is much more vascularized than white matter, while lesions associated with spinal DCS are always detected in the white matter.

We postulate that, in addition to the mechanisms described above, spinal cord injury could result from the combined effects of the above mechanisms through the following sequence of events (**Figure 22B**) (Arieli, 2018b):

- Shunted arterial bubbles (or distal AHS bubbles) come first in the grey matter (Arieli, 2021b).
- They trigger endothelial damage, platelet activation, and neutrophil activation, which generate cytokines, complement factors, and so forth, which build up local inflammation and edema.
- Edema increases local tissue pressure and reduces blood perfusion in the surrounding white matter. This mechanical effect results from the medullar tissue being encapsulated in a vertebra, which does not allow tissue expansion (Hills and James, 1982).
- This exposes the local tissue nanobubbles on white matter AHS to greater diffusion distances and causes them to grow. Several sites can be affected simultaneously.

Consequences include grey matter endothelial damage, arterial bubbles blocking blood supply, or a succession of episodes of blood occlusion followed by reperfusion, resulting in cumulative endothelial dysfunction of the grey matter vessels.

### 14.5 Model for the pathogenesis of type I DCS

Pain in or around a joint is a common symptom of DCS, classified as type I in the US Navy diving manual. It typically appears in saturation decompressions in the last 10 msw of the ascent (Imbert and Bontoux, 1988) but is also very common in commercial air diving (Shields et al., 1989).

The pain is generally diffuse, radiating, and changing location from hour to hour. It can sometimes be mistaken for muscle pain.

Joint pain responds well to recompression, meaning that Boyle’s law acts on reducing the volume of a gas phase. The Comex medical book used simple recompression to 12 msw on oxygen, called table Cx12, which proved very efficient in treating pain-only symptoms (Imbert).

In 1986, the Comex introduced staged rather than continuous saturation decompression so chamber operators could perform other tasks during stops. In the final 10 msw, divers developed mild knee “niggles” whenever stop depth changed, interpreted as Boyle’s law acting on joint bubbles. The staged option was removed and the symptoms disappeared.

Three possible locations have been identified for this gas phase (**Figure 22C**):

- Batt et al. used the sheep model to produce joint pain and observed bubbles in the bone head below the cartilage (Ball et al., 1999). This results in diffuse, dull pain that changes location, often described as muscular pain or strain. Depending on how the accident is treated, the evolution can lead to dysbaric osteonecrosis when the lesion is in the head of the bone (Marzella and Yin, 1994) or to arthritis when the lesion is below the cartilage (Lehner et al., 1997).
- In some cases, the pain can be acute. Such pain is associated with the mechanical distortion of nerve endings and strongly suggests the physical action of a bubble in a tendon or connective tissue. In a famous animal experiment with the kangaroo rat, he associated tail biting with the location of the pain, observing bubbles in the tail tendon (Hills and Butler, 1978). Strauss et al. have suggested that this acute pain is caused by the distortion of the sensory organelles, stretching the Ruffini type-2 corpuscles (Strauss et al., 2018a).
- Bubbles have also been identified in the synovial interspace between joints, but these bubbles are reported as being asymptomatic (Jitsuiki et al., 2021).

Hills showed that joints use oligolamellar phospholipids for lubrication (Gale et al., 2007). He suggested that phospholipid surfaces could provide the sites (the AHS) for extravascular nucleation and expansion of bubbles in the synovial compartment, connective tissue, or the bone head.

Joint synovia and connective tissues have poor or no perfusion, and gas exchange is controlled by diffusion. The development and growth of microbubbles responsible for pain-only DCS are slow and diffusion-controlled.

Type I symptoms are not necessarily the result of a single dive but rather of a series of dives, as recorded with air diving worksites in the Comex database (Imbert and Bontoux, 1987). They may also result from a long decompression. In saturation, type I symptoms occur in the last few meters of the ascent after several days of decompression (Imbert and Bontoux, 1988). In these cases, their relationship to decompression inflammation seems very likely.

### 14.6 Model for constitutional manifestations

Referring to Mitchell’s publication, manifestations such as fatigue, headache, weakness, and so forth are considered “constitutional” and related to sympathetic or general reaction (Mitchell, 2024).

Fatigue, headache, paleness, weakness, anxiety, shock, and unconsciousness are considered manifestations of the cardiopulmonary system. These manifestations can occur with any symptom, depending on the psychological impact and the many physiological/biological reactions induced by DCS/DCI over time.

Constitutional manifestations may indicate the severity of the case and the diver’s growing concern about their situation. Headache, often reported with cerebral DCS, is linked to cerebral vasodilatation and may result from bubble-induced endothelial dysfunction.

Paraesthesia are characteristic of the early development of cerebral and medullar symptoms. However, paraesthesia are neurological symptoms not necessarily related to decompression; patients with medullar infarcts report paraesthesia.

Paraesthesia may also have a metabolic origin. In particular, dehydration and hyperventilation change the local ionic concentrations. A reduction in blood CO_2_ level increases the blood pH. This can change the local availability of Ca^++^ and modify the sensitivity of the neurological pathways. Anxiety can also lead to hyperventilation and paraesthesia.

## 15 Algorithm structure

The concepts identified in the previous chapters are the pieces of our puzzle. We assemble them to present a new way of structuring decompression algorithms. We recognize that this theoretical model involves simplifications and approximations. We hope that new data and knowledge will allow us to improve and complete this puzzle (**Figure 23**).

**Figure 23:**
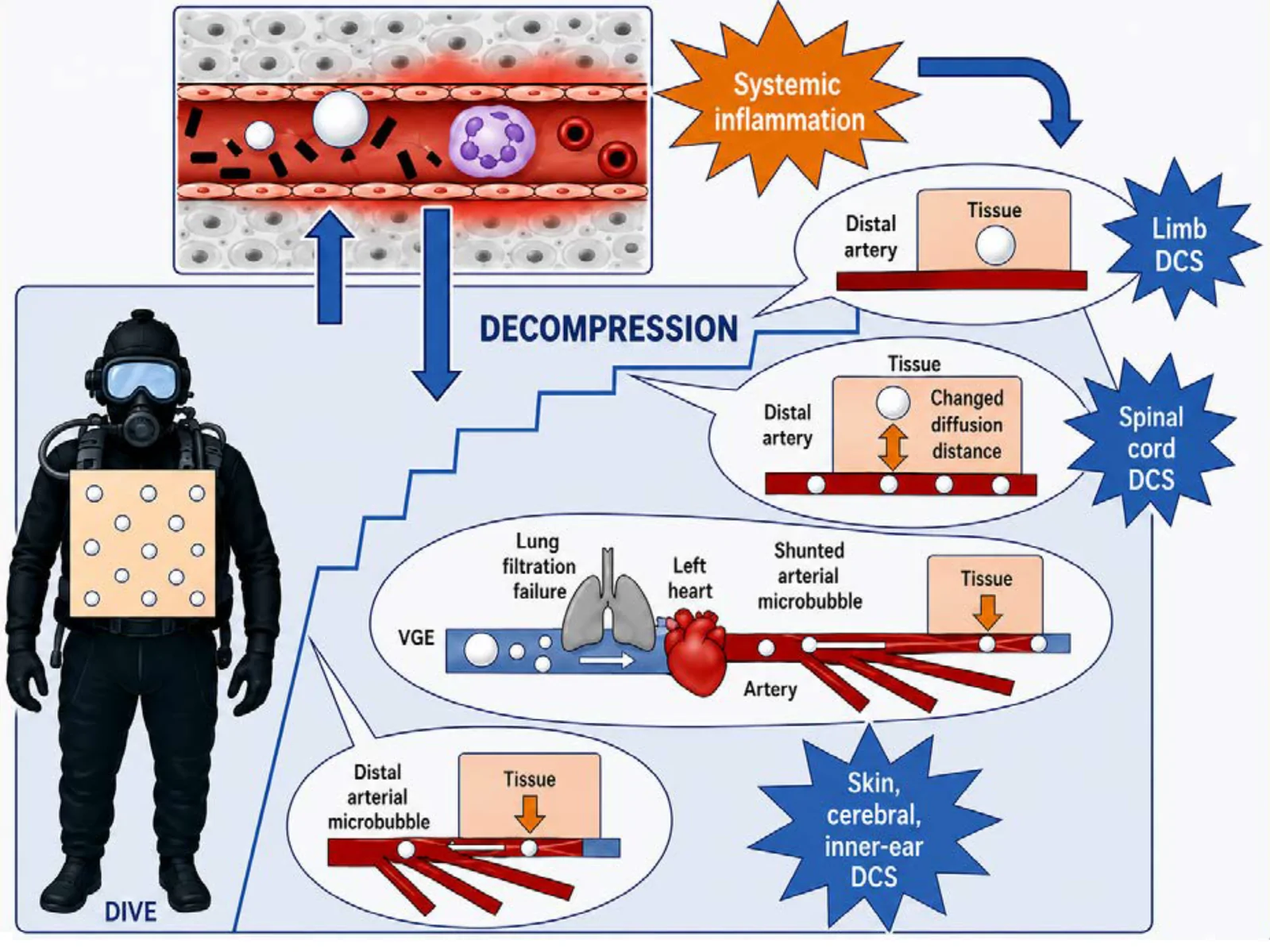
The sequence of the four physical bubble scenarios staged along the decompression, affecting the 5 target organs, and the implication of MP production and systemic inflammation.

### 15.1 The diver

The diver comes as the first source of variability. Their pre-existing population of gas nuclei depends on his AHS density, and the volume of gas nuclei depends on his metabolism. These factors depend on metabolism and determine their VGE level after a given dive.

### 15.2 The two dimensions of the decompression stress

We have split the decompression stress into two dimensions: physical and physiological. The physical aspect of this stress is well-suited to mathematical modeling. However, our current knowledge and experience do not allow us to model and quantify the inflammatory dimension of this stress. This stress is currently managed by empirical rules set by policies.

### 15.3 The three AHS locations

The physical aspect of the decompression stress remains accessible to mathematical modeling and forms the core of decompression algorithms. We have based our theoretical approach on the three different locations of the AHS:

1. Distal arterial AHS.
2. Endothelial AHS located at the tissue level.
3. Extravascular AHS.

Boyle’s law activates distal arterial AHS during rapid pressure changes (ascent to the first stop or yo-yo diving).

Other AHS activation depends on local supersaturation and gas exchange. Depending on whether these exchanges are limited by perfusion or diffusion, they activate sequentially along the ascent. Perfusion and diffusion are likely to combine as well, for instance in the spinal cord white matter.

### 15.4 The four bubble scenarios

Based on the AHS model above, we have identified four bubble scenarios. The physical processes in these scenarios suggest they are triggered sequentially and overlap along the ascent.

1. In the initial part of the decompression, Boyle’s law prevails. The ascent to the first stop activates the distal arterial AHS and generates distal arterial microbubbles.
2. Immediately after arriving at the first stop depth, the arterial bubble scenario is activated. The gas gradients are established in the tissues. Venous AHS generate microbubbles that become VGE. Failure of lung filtration may produce shunted arterial bubbles.
3. In the last part of the decompression and post-dive interval, the extravascular bubble scenario may account for musculoskeletal DCS. These slow processes involve bubble growth based on a tissue diffusion gradient.
4. Between the arterial and extravascular bubble scenarios, there is a period during which arterial bubbles interfere with the spinal cord diffusion gas exchange and may initiate white matter bubble growth.

### 15.5 The five target organs

The above bubble scenarios suggest the following sequences of events leading to DCS symptoms in the five target organs identified:

- Arterial bubbles control the first phase of the decompression. The mechanism involves a shunted arterial bubble passing through the lung or a right-to-left heart shunt, and in some cases, possibly a distal arterial bubble. For skin and inner-ear DCS, the process requires supersaturated target tissue to fuel arterial bubble growth.
- For cerebral DCS, a shower of arterial bubbles could produce DCS symptoms just by mechanical damage to the blood–brain barrier. The delay in symptom onset is short.
- The tissue bubble controls the last phase of decompression. The mechanisms involve extravascular AHS and slow, diffusion-controlled gas exchange that occurs by the end of decompression or in the few hours following decompression. It accounts for the pain-only DCS class. The delay in symptom onset is long.
- During intermediate decompression, these mechanisms may combine in the spinal cord. Arterial bubbles may damage grey-matter endothelium, cause leakage or hemorrhage, or increase diffusion distance by reducing perfusion, thereby promoting bubble growth in white-matter AHS. Symptom delay depends on the mechanisms involved.

### 15.6 The multiple stressors of dive and decompression

Several physiological components of dive and decompression stress can promote MP production and endothelial dysfunction. They do not alter the physical scenarios but change microbubble–endothelium interactions and thus susceptibility. Assessing inflammatory stress is important for post-dive intervals, multiday patterns, and possible long-term effects.

## 16 Decompression strategy

In a simple animal experiment, Hills showed how a rapid excursion to the surface, followed by an immediate recompression to the first stop, changed joint pain into neurological symptoms (Hills, 1971). These two profiles obviously promote different bubble scenarios that underlie distinct strategies.

### 16.1 Concept: decompression strategy

Options for a decompression resemble the ones for a marathon: start fast and finish slow, or the opposite. In diving, the objective is to manage the decompression stress. A decompression strategy is an informed or intuitive decision about managing the various bubble scenarios and the cumulative inflammation during the ascent.

Accordingly, one strategy uses a relatively rapid ascent to a shallower stop followed by longer shallow decompression; another begins with deeper stops and may shorten the final shallow phase. The sequence of bubble scenarios and operational constraints determines which compromise is appropriate. No single profile can optimize every mechanism simultaneously.

The decompression profile expresses the selected strategy, which depends on mission, gas mixtures, equipment, and decompression method. Thus, rebreather tables for military mine-warfare divers differ from those for commercial divers working on North Sea platforms.

Strategies must adapt to operational conditions.

Mediterranean coral divers work on deep, isolated rocks and decompress mid-water. They only carry the bottom mix and rely on their support vessel to launch an umbilical that supplies the deco mixes. Their objective is to deploy a surface buoy marker as soon as possible to get spotted by the vessel and ensure the umbilical arrives on time. Their strategy is based on a fast ascent rate and an initial stop at around 30–40 msw. Once spotted, they recover the umbilical and breathe oxygen between 15 and 9 msw in water for a 10–15 min stop, and then surface to a deck chamber where they are recompressed to 12 msw for a massive oxygen dose. Their strategy, as we understand it, is to create a large gradient at the start of the ascent to wash out the inert gas, and then treat the resulting rush of arterial or venous bubbles with deep, prolonged oxygen breathing sessions. This strategy has limits but coral divers’ bottom times do not exceed 15 min deeper than 60 msw (**Figure 24A**).

**Figure 24:**
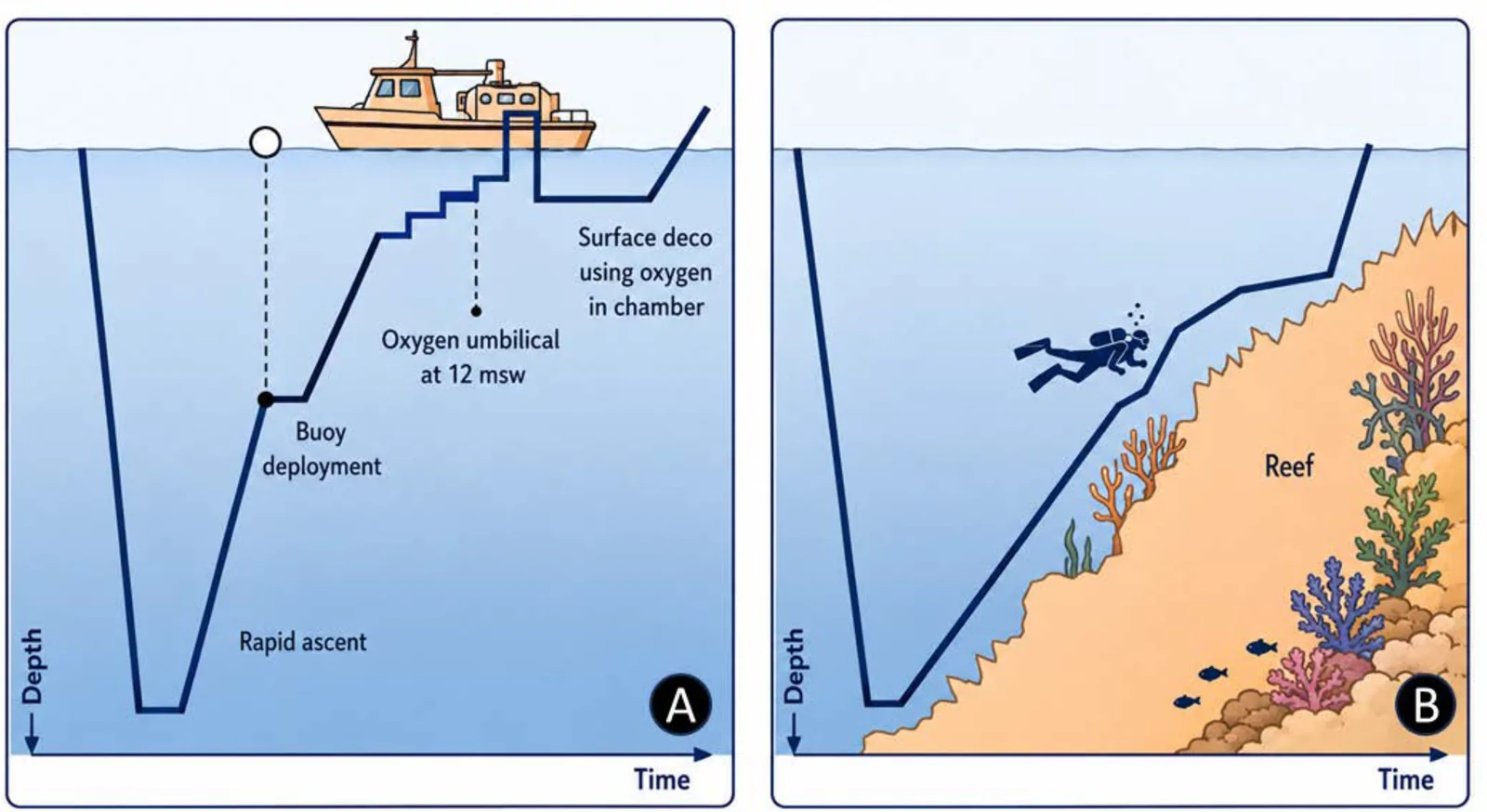
**(A)** A typical Mediterranean coral diver profile. After leaving the bottom, the diver ascends to 40 msw to launch a marker buoy. The standby vessel then comes and deploys an oxygen umbilical. The diver completes decompression at the surface using oxygen. **(B)** A typical recreational diver on a reef dive, slowly ascending along the reef without any stop.

Recreational divers often conduct their dive by slowly ascending along the reef without exceeding their ceiling depth until it equals the surface. They then ascend directly to the surface. Their objective is to avoid any stop. Their strategy is to minimize gas gradients and bubbling. This strategy also has limits because delayed gas washout could create tissue supersaturation by the end of the decompression (Angelini et al., 2022) (**Figure 24B**).

Strategies are sometimes imposed by the local conditions. A cave diver must follow the cave profile they are exploring. A North Sea diver in 6 m swell must use surface decompression as they cannot carry out in-water stops.

### 16.2 Perspective: the open decompression strategy

Decompression procedures provide options for these strategies:

- Decompression profile (first stop depth, last stop time).
- Raised breathing PO2 (decompression mixes, pure oxygen)
- Inert gas switches.
- Ascent technique (in-water deco, surface decompression, TUP decompression)

Decompression strategies may vary from table to table. Among the air tables used in the offshore industry, the French MT 92 and the DCIEM air tables are very similar in duration and profiles. However, they differ from the US Navy air tables, which use shallower first stops but longer 3 msw stops, and the Dutch NDC air tables, which use deeper first stops. All these tables are widely used in commercial diving, and if some tables lack documented track records, they all benefit from a good reputation among diving supervisors (Shields et al., 1989).

This suggests that:

- Different decompression strategies (and profiles) can achieve similar safety performances.
- Strategies can be optimized once the decompression constraints are known. For instance, the decompression procedure may vary from in-water to surface decompression, from an open-circuit dive to a CCR dive.

There is no ultimate decompression algorithm, but strategies derived from operational experience. As institutions validate procedures against their own data, profiles may therefore differ within validated domains.

### 16.3 Perspective: testing the framework

Our proposed framework requires validation of its main predictions that could be conducted the following way:

- Prediction 1—The density of AHS should predict individual VGE production. Confirmation: a measure of site density correlating with repeated VGE measurements in the same individuals. Refutation: no association once exposure and gas load are controlled. Requirement: a method to quantify site density, which does not exist *in vivo*; an *ex-vivo* assay on vascular tissue, or a validated indirect index, would be the first step. This prediction cannot currently be tested.
- Prediction 2—Modification of the site population should alter VGE production independently of the dissolved gas load. Confirmation: an intervention that changes VGE at matched tissue gas load. Refutation: the effect of vibration, exercise or oxygen preconditioning disappearing once gas load is matched. Requirement: protocols in which gas load is held constant, which the existing preconditioning studies have not generally done. This is testable now and is the most immediately informative of the five.
- Prediction 3—Distal arterial sites should be demonstrable *in-vivo* or in a physiologically relevant model. Confirmation: identification of hydrophobic deposits on arteriolar endothelium capable of hosting a gas phase, and of bubble release from them under decompression. Refutation: a careful search in a relevant model finding. This is the weakest link in the framework: the site has been observed only *ex-vivo*.
- Prediction 4—Inflammatory susceptibility should predict DCS risk or recovery independently of VGE grade. Confirmation: a biomarker panel adding predictive value over VGE grade and exposure together. Refutation: no added value. Requirement: prospective series large enough for an outcome of the order of one per thousand, which points to pooled international databases.
- Prediction 5—Inflammatory biomarkers should show a measurable cumulative dynamic during repetitive diving. Confirmation: markers that do not return to baseline across a multiday series and that track the recovery intervals imposed. Refutation: return to baseline between exposures. Requirement: repeated sampling across a diving series rather than before and after a single dive. This is testable now and would bear directly on the empirical rules currently used to set post-dive surface intervals and days off.
- Predictions 2 and 5 can be tested with existing methods, and prediction 4 can be tested with access to a sufficiently large series. Predictions 1 and 3 await a method that does not yet exist. Refutation of predictions 1 and 3 would remove the arterial site scenario from the framework without disturbing the rest of it.

## 17 Conclusion

We have set out the principles underlying the construction of decompression procedures, reviewed the concepts on which they rest, and proposed a “Neo-Haldanian” framework connecting bubble physics to decompression physiology.

We have shown that designing a decompression procedure requires bolting together five layers of complexity (**Figure 25**).

**Figure 25:**
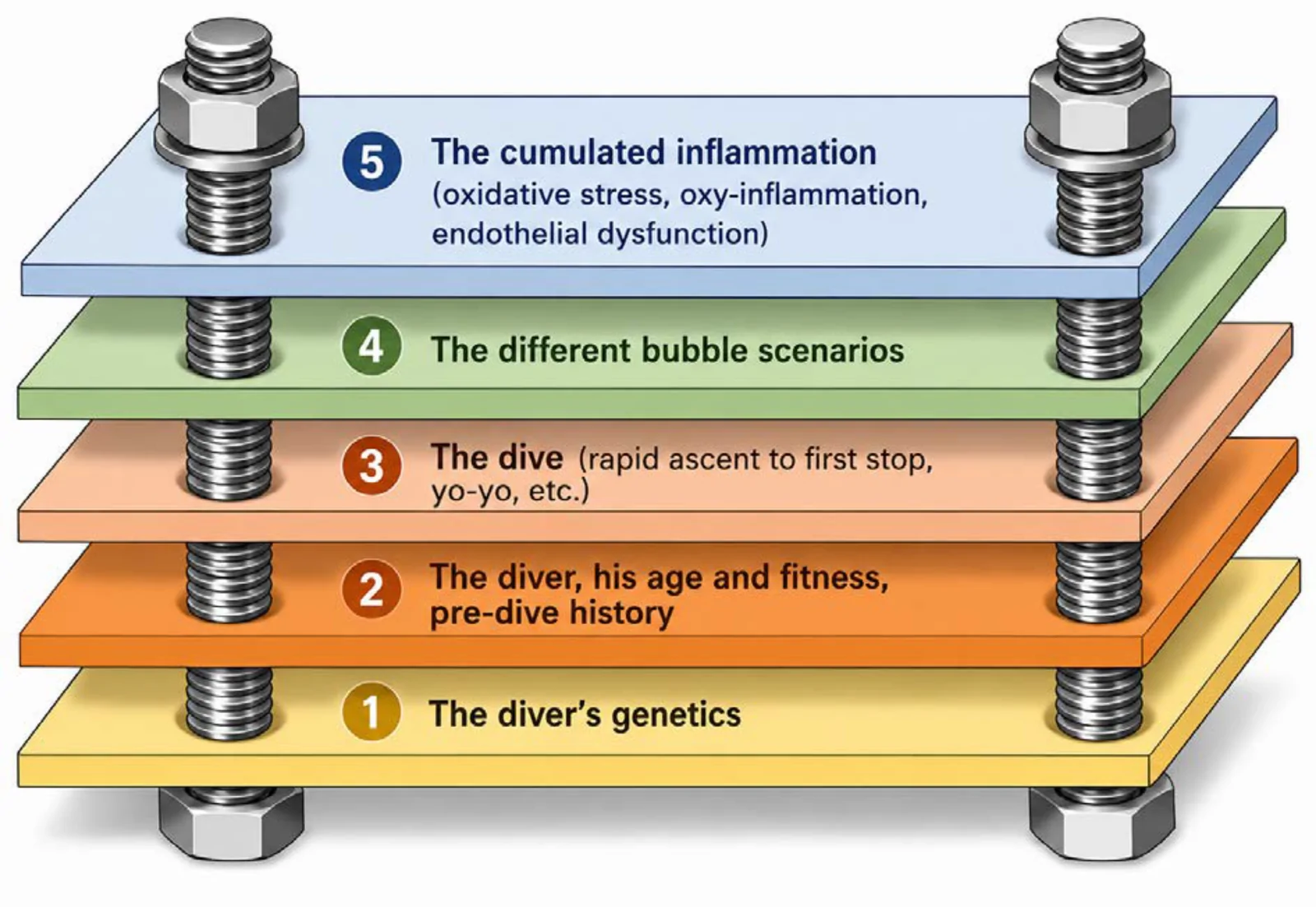
The five different layers of complexity involved in computing practical decompression procedures.

- The diver’s genetics.
- The diver’s individual susceptibility (age, fitness, diet, right-to-left shunt, DPPC) and the dive history (repetitive diving, multi-day diving, progressive adaptation to depth, diving long-term health effects).
- The dive characteristics (rapid ascents to first stop, yo-yo diving, surface decompression method, etc.).
- The various bubble scenarios.
- The buildup of dive and decompression inflammation, oxidative stress, oxy-inflammation, and endothelial damage changes the divers’ susceptibility to DCS.

This complexity brings variability. We discuss causes of individual variability, which include both operational factors (dive conditions, dive profile, repetitive diving, multiday diving) and physiological factors (genetics, preexisting gas nuclei population, lung bubble filtration, inflammation state).

The complexity also provides options that allow the diver to develop specific decompression strategies.

We conclude that there is no ultimate decompression algorithm, but rather various strategies adapted to different operational experiences.

## References

[B1] Akaike H. (2003). A new look at the statistical model identification. IEEE Trans. Autom. Control 19, 716–723. doi: 10.1109/tac.1974.110070525079929

[B2] Alvarado R. Scheven U. M. Meiners J. C. (2024). Real-time imaging of decompression gas bubble growth in the spinal cord of live rats. Magn. Reson. Med. 92, 1632–1637. doi: 10.1101/2024.02.09.578916 38651172

[B3] Angelini S. A. Tonetto L. Lang M. A. (2022). Ceiling-controlled versus staged decompression: Comparison between decompression duration and tissue tensions. Diving Hyperb Med. 52, 7–15. doi: 10.28920/dhm52.1.7-15 35313367 PMC9016140

[B4] Arieli R. (2017). Nanobubbles form at active hydrophobic spots on the luminal aspect of blood vessels: consequences for decompression illness in diving and possible implications for autoimmune disease-an overview. Front. Physiol. 8, 591. doi: 10.3389/fphys.2017.00591 28861003 PMC5559548

[B5] Arieli R. (2018a). Do skin rash and cutis marmorata stem from lamellar bodies within the skin? Diving Hyperb Med. 48, 114. doi: 10.28920/dhm48.2.114 29888386 PMC8349700

[B6] Arieli R. (2018b). Extravascular hydrophobic surfaces, fat droplets, and the connection with decompression illness: spinal, joint pain, and dysbaric osteonecrosis. Front. Physiol. 9, 305. doi: 10.3389/fphys.2018.00305 29636703 PMC5880943

[B7] Arieli R. (2019a). Calculated risk of pulmonary and central nervous system oxygen toxicity: a toxicity index derived from the power equation. Diving Hyperb Med. 49, 154–160. doi: 10.28920/dhm49.3.154-160 31523789 PMC6881196

[B8] Arieli R. (2019b). Taravana, vestibular decompression illness, and autochthonous distal arterial bubbles. Respir. Physiol. Neurobiol. 259, 119–121. doi: 10.1016/j.resp.2018.08.010 30172778

[B9] Arieli R. (2019c). *In vitro* evidence of decompression bubble dynamics and gas exchange on the luminal aspect of blood vessels: Implications for size distribution of venous bubbles. Physiol. Rep. 7, e14317. doi: 10.14814/phy2.14317 31876064 PMC6930933

[B10] Arieli R. (2021a). Does the most potent lung surfactant dipalmitoylphosphatidylcholine pose a risk for decompression illness in diving mammals? Respir. Physiol. Neurobiol. 290, 103681. doi: 10.1016/j.resp.2021.103681 33962027

[B11] Arieli R. (2021b). Diving-related disorders in breath-hold divers could be explained with the distal arterial bubble hypothesis. Diving Hyperb Med. 51, 382–383. doi: 10.28920/dhm51.4.382-383 34897605 PMC8920904

[B12] Arieli R. (2021c). Endothelial injury in diving: atomic force-, electronic-, and light-microscopy study of the ovine decompressed blood vessels. Front. Physiol. 12, 767435. doi: 10.3389/fphys.2021.767435 34721086 PMC8554307

[B13] Arieli R. (2022). The increased protein tau in blood after SCUBA diving can be related to distal arterial bubbles. Eur. J. Appl. Physiol., 2315–2316. doi: 10.1007/s00421-022-04993-5 35752661

[B14] Arieli R. (2023). Distal arterial bubble: an alternative mechanism underlying vestibular decompression illness. J. Appl. Physiol. (1985) 134, 593–594. doi: 10.1152/japplphysiol.00003.2023 36897585

[B15] Arieli R. (2026). Opinion: bubbles or microparticles — which are the main initial cause of decompression illness? Undersea Hyperbaric Med. 42826432

[B16] Arieli R. Arieli U. Marmur A. (2015). Bubble size on detachment from the luminal aspect of ovine large blood vessels after decompression: the effect of mechanical disturbance. Respir. Physiol. Neurobiol. 216, 1–8. doi: 10.1016/j.resp.2015.05.001 26003848

[B17] Arieli R. Aviner B. (2020). Acclimatization and deacclimatization to oxygen: determining exposure limits to avoid CNS O(2) toxicity in active diving. Front. Physiol. 11, 1105. doi: 10.3389/fphys.2020.01105 33013472 PMC7498636

[B18] Arieli R. Boaron E. Arieli Y. Abramovich A. Katsenelson K. (2011). Oxygen pretreatment as protection against decompression sickness in rats: pressure and time necessary for hypothesized denucleation and renucleation. Eur. J. Appl. Physiol. 111, 997–1005. doi: 10.1007/s00421-010-1725-y 21082196

[B19] Arieli R. Khatib S. Khattib A. Bukovetzky E. Gottfried O. D. (2021). Is the probable spillage of the lung surfactant dipalmitoylphosphatidylcholine the ultimate source of diabetes type 1? Respir. Physiol. Neurobiol. 286, 103615. doi: 10.1016/j.resp.2020.103615 33421610

[B20] Arieli R. Khatib S. Vaya J. (2016). Presence of dipalmitoylphosphatidylcholine from the lungs at the active hydrophobic spots in the vasculature where bubbles are formed on decompression. J. Appl. Physiol. (1985) 121, 811–815. doi: 10.1152/japplphysiol.00649.2016 27516538

[B21] Arieli R. Marmur A. (2013). Dynamics of gas micronuclei formed on a flat hydrophobic surface, the predecessors of decompression bubbles. Respir. Physiol. Neurobiol. 185, 647–652. doi: 10.1016/j.resp.2012.11.020 23246801

[B22] Arieli R. Marmur A. (2014). Ex vivo bubble production from ovine large blood vessels: size on detachment and evidence of "active spots. Respir. Physiol. Neurobiol. 200, 110–117. doi: 10.1016/j.resp.2014.05.014 24933644

[B23] Arieli R. Marmur A. (2016). Expansion of bubbles under a pulsatile flow regime in decompressed ovine blood vessels. Respir. Physiol. Neurobiol. 222, 1–5. doi: 10.1016/j.resp.2015.11.010 26592146

[B24] Arieli R. Marmur A. (2017). A biophysical vascular bubble model for devising decompression procedures. Physiol. Rep. 5. doi: 10.14814/phy2.13191 28320890 PMC5371562

[B25] Arya A. K. Balestra C. Bhopale V. M. Tuominen L. J. Räisänen-Sokolowski A. Dugrenot E. . (2023). Elevations of extracellular vesicles and inflammatory biomarkers in closed circuit SCUBA divers. Int. J. Mol. Sci. 24, 5969. doi: 10.3390/ijms24065969 36983042 PMC10053377

[B26] Balestra C. Germonpré P. Marroni A. (1998). Intrathoracic pressure changes after Valsalva strain and other maneuvers: implications for divers with patent foramen ovale. Undersea Hyperb Med. 25, 171–174. 9789337

[B27] Balestra C. (2014). The lymphatic pathway for microbubbles. Diving Hyperb Med. 44, 1. 24687477

[B28] Balestra C. Germonpre P. (2016). Correlation between patent foramen ovale, cerebral "lesions" and neuropsychometric testing in experienced sports divers: Does diving damage the brain? Front. Psychol. 7, 696. doi: 10.3389/fpsyg.2016.00696 27242609 PMC4863080

[B29] Balestra C. Cimino F. Theunissen S. Snoeck T. Provyn S. Canali R. . (2016a). A red orange extract modulates the vascular response to a recreational dive: a pilot study on the effect of anthocyanins on the physiological consequences of scuba diving. Nat. Prod. Res. 30, 2101–2106. doi: 10.1080/14786419.2015.1107062 26548425

[B30] Balestra C. Theunissen S. Papadopoulou V. Le Mener C. Germonpré P. Guerrero F. . (2016b). Pre-dive whole-body vibration better reduces decompression-induced vascular gas emboli than oxygenation or a combination of both. Front. Physiol. 7, 586. doi: 10.3389/fphys.2016.00586 27965591 PMC5127795

[B31] Balestra C. Germonpre P. Rocco M. Biancofiore G. Kot J. (2019). Diving physiopathology: the end of certainties? Food for thought. Minerva Anestesiol 85, 1129–1137. doi: 10.23736/s0375-9393.19.13618-8 31238641

[B32] Balestra C. Arya A. K. Leveque C. Virgili F. Germonpré P. Lambrechts K. . (2022). Varying oxygen partial pressure elicits blood-borne microparticles expressing different cell-specific proteins-toward a targeted use of oxygen? Int. J. Mol. Sci. 23. doi: 10.3390/ijms23147888 35887238 PMC9322965

[B33] Balestra C. Leveque C. Mrakic-Sposta S. Coulon M. Tumbarello R. Vezzoli A. . (2024a). Inert gas mild pressure action on healthy humans: The "IPA" study. Int. J. Mol. Sci. 25. doi: 10.3390/ijms252212067 39596136 PMC11593890

[B34] Balestra C. Lévêque C. Mrakic-Sposta S. Vezzoli A. Wauthy P. Germonpré P. . (2024b). Physiology of deep closed circuit rebreather mixed gas diving: vascular gas emboli and biological changes during a week-long liveaboard safari. Front. Physiol. 15, 1395846. doi: 10.3389/fphys.2024.1395846 38660539 PMC11040087

[B35] Ball R. Lehner C. E. Parker E. C. (1999). Predicting risk of decompression sickness in humans from outcomes in sheep. J. Appl. Physiol. (1985) 86, 1920–1929. doi: 10.1152/jappl.1999.86.6.1920 10368357

[B36] Barak O. F. Madden D. Lovering A. T. Lambrechts K. Ljubkovic M. Dujic Z. (2015). Very few exercise-induced arterialized gas bubbles reach the cerebral vasculature. Med. Sci. Sports Exerc 47, 1798–1805. doi: 10.1249/mss.0000000000000625 25628180

[B37] Behnke A. R. (1967). “ The isobaric (oxygen window) principle of decompression,” in Trans. Third Annual Conf. Marine Tech, Soc, vol. 1. , 213.

[B38] Bennett P. B. Marroni A. Cronje F. J. Cali-Corleo R. Germonpre P. Pieri M. . (2007). Effect of varying deep stop times and shallow stop times on precordial bubbles after dives to 25 msw (82 fsw). Undersea Hyperb Med. 34, 399–406. 18251436

[B39] Besnard S. Capri A. Philippot M. Hervé P. Porcher M. A. Arbeille P. (2006). Vascular distribution of an ultrasound contrast agent used to simulate decompression bubbles. Aviat. Space Environ. Med. 77, 846–851. 16909880

[B40] Bhat A. R. Arya A. K. Bhopale V. M. Imtiyaz Z. Thom S. R. (2024a). Recombinant human plasma gelsolin suppresses persistent neuroinflammation and restores hippocampal neurogenesis in murine model of decompression sickness. J. Neurophysiol. doi: 10.1152/jn.00332.2024 39475490 PMC11687844

[B41] Bhat A. R. Arya A. K. Bhopale V. M. Imtiyaz Z. Xu S. Bedir D. . (2024b). Persistent neuroinflammation and functional deficits in a murine model of decompression sickness. J. Appl. Physiol. (1985) 137, 63–73. doi: 10.1152/japplphysiol.00097.2024 38660728 PMC11389893

[B42] Bhat A. R. Arya A. K. Imtiyaz Z. Xu S. Thom S. R. (2025). Critical role for scavenger receptor CD36 in microparticle-mediated neuroinflammation in a murine model of decompression sickness. J. Appl. Physiol. (1985) 139, 1112–1124. doi: 10.1152/japplphysiol.00484.2025 40987527

[B43] Bhopale V. M. Ruhela D. Brett K. D. Nugent N. Z. Fraser N. K. Levinson S. L. . (2021). Plasma gelsolin modulates the production and fate of IL-1β-containing microparticles following high-pressure exposure and decompression. J. Appl. Physiol. (1985) 130, 1604–1613. doi: 10.1152/japplphysiol.01062.2020 33764168 PMC8354825

[B44] Bhullar J. Bhopale V. M. Yang M. Sethuraman K. Thom S. R. (2016). Microparticle formation by platelets exposed to high gas pressures - An oxidative stress response. Free Radic. Biol. Med. 101, 154–162. doi: 10.1016/j.freeradbiomed.2016.10.010 27751909

[B45] Blatteau J. E. Gaillard S. De Maistre S. Richard S. Louges P. Gempp E. . (2018). Reduction in the level of plasma mitochondrial DNA in human diving, followed by an increase in the event of an accident. Front. Physiol. 9, 1695. doi: 10.3389/fphys.2018.01695 30555340 PMC6282000

[B46] Blatteau J. E. Gempp E. Balestra C. Mets T. Germonpre P. (2008). Predive sauna and venous gas bubbles upon decompression from 400 kPa. Aviat. Space Environ. Med. 79, 1100–1105. doi: 10.3357/asem.2377.2008 19070305

[B47] Blatteau J. E. Hugon M. Gardette B. Sainty J. M. Galland F. M. (2005). Bubble incidence after staged decompression from 50 or 60 msw: effect of adding deep stops. Aviat. Space Environ. Med. 76, 490–492. 15892549

[B48] Blatteau J. E. Souraud J. B. Gempp E. Boussuges A. (2006). Gas nuclei, their origin, and their role in bubble formation. Aviat. Space Environ. Med. 77, 1068–1076. 17042253

[B49] Bosco G. Rizzato A. Quartesan S. Camporesi E. Mrakic-Sposta S. Moretti S. . (2018). Spirometry and oxidative stress after rebreather diving in warm water. Undersea Hyperb Med. 45, 191–198. doi: 10.22462/03.04.2018.6 29734571

[B50] Bove A. A. (1998). Risk of decompression sickness with patent foramen ovale. Undersea Hyperb Med. 25, 175–178. 9789338

[B51] Boycott A. E. Damant G. C. Haldane J. S. (1908). The prevention of compressed-air illness. J. Hyg. (Lond) 8, 342–443. doi: 10.1017/s0022172400003399 20474365 PMC2167126

[B52] Brett K. D. Nugent N. Z. Fraser N. K. Bhopale V. M. Yang M. Thom S. R. (2019). Microparticle and interleukin-1β production with human simulated compressed air diving. Sci. Rep. 9, 13320. doi: 10.1038/s41598-019-49924-1 31527725 PMC6746808

[B53] Brubakk A. O. Duplancic D. Valic Z. Palada I. Obad A. Bakovic D. . (2005). A single air dive reduces arterial endothelial function in man. J. Physiol. 566, 901–906. doi: 10.1113/jphysiol.2005.089862 15961424 PMC1464788

[B54] Brubakk A. O. Mollerlokken A. (2009). The role of intra-vascular bubbles and the vascular endothelium in decompression sickness. Diving Hyperb Med. 39, 162–169. 22753245

[B55] Brubakk A. O. Peterson R. Grip A. Holand B. Onarheim J. Segadal K. . (1986). Gas bubbles in the circulation of divers after ascending excursions from 300 to 250 msw. J. Appl. Physiol. (1985) 60, 45–51. doi: 10.1152/jappl.1986.60.1.45 3511026

[B56] Bühlmann A. A. (2013). Decompression - Decompression Sickness (Berlin, Germany: Springer Science & Business Media).

[B57] Butler B. D. Hills B. A. (1979). The lung as a filter for microbubbles. J. Appl. Physiol. Respir. Environ. Exerc Physiol. 47, 537–543. doi: 10.1152/jappl.1979.47.3.537 533747

[B58] Butler B. D. Hills B. A. (1985). Transpulmonary passage of venous air emboli. J. Appl. Physiol. (1985) 59, 543–547. doi: 10.1152/jappl.1985.59.2.543 4030608

[B59] Cantais E. Louge P. Suppini A. Foster P. P. Palmier B. (2003). Right-to-left shunt and risk of decompression illness with cochleovestibular and cerebral symptoms in divers: case control study in 101 consecutive dive accidents. Crit. Care Med. 31, 84–88. doi: 10.1097/00003246-200301000-00013 12544998

[B60] Cardús J. Burgos F. Diaz O. Roca J. Barberà J. A. Marrades R. M. . (1997). Increase in pulmonary ventilation-perfusion inequality with age in healthy individuals. Am. J. Respir. Crit. Care Med. 156, 648–653. 10.1164/ajrccm.156.2.9606016 9279253

[B61] Carturan D. Boussuges A. Burnet H. Fondarai J. Vanuxem P. Gardette B. (1999). Circulating venous bubbles in recreational diving: relationships with age, weight, maximal oxygen uptake and body fat percentage. Int. J. Sports Med. 20, 410–414. doi: 10.1055/s-2007-971154 10496123

[B62] Carturan D. Boussuges A. Molenat F. Burnet H. Fondarai J. Gardette B. (2000). Ascent rate and circulating venous bubbles in recreational diving. Int. J. Sports Med. 21, 459–462. doi: 10.1055/s-2000-7411 11071045

[B63] Castagna O. Brisswalter J. Vallee N. Blatteau J. E. (2011). Endurance exercise immediately before sea diving reduces bubble formation in scuba divers. Eur. J. Appl. Physiol. 111, 1047–1054. doi: 10.1007/s00421-010-1723-0 21107599

[B64] Castagna O. Gempp E. Blatteau J. E. (2009). Pre-dive normobaric oxygen reduces bubble formation in scuba divers. Eur. J. Appl. Physiol. 106, 167–172. doi: 10.1007/s00421-009-1003-z 19219451

[B65] Caupin F. Herbert E. (2006). Cavitation in water: a review. C.R. Phys. 7, 1000–1017. doi: 10.1016/j.crhy.2006.10.01542574925

[B66] Chappell M. A. Payne S. J. (2006). A physiological model of the release of gas bubbles from crevices under decompression. Respir. Physiol. Neurobiol. 153, 166–180. doi: 10.1016/j.resp.2005.10.006 16309977

[B67] Cialoni D. Marabotti C. Sponsiello N. Pieri M. Balestra C. Lucchini V. . (2015). Genetic predisposition to breath-hold diving-induced hemoptisys: Preliminary study. Undersea Hyperbaric Med. 42, 75–83. 26094307

[B68] Cialoni D. Pieri M. Balestra C. Marroni A. (2017). Dive risk factors, gas bubble formation, and decompression illness in recreational SCUBA diving: analysis of DAN Europe DSL data base. Front. Psychol. 8, 1587. doi: 10.3389/fpsyg.2017.01587 28974936 PMC5610843

[B69] Clark J. M. (1993). “ Oxygen toxicity,” in The Physiology and Medicine of Diving; 4th Edition. Eds. Bennet E. DH ( WB Saunders Company Ltd, London, UK), 220–238.

[B70] Daubresse L. Marlinge M. Lavner H. (2026). Therapeutic potential of GYY4137 in reducing oxidative stress and mortality in experimental decompression sickness. Sci. Rep. 16, 8874. doi: 10.1038/s41598-026-41352-2 41786804 PMC12988038

[B71] Daubresse L. Portas A. Bertaud A. (2024). CO2 breathing prior to simulated diving increases decompression sickness risk in a mouse model: the microbiota trail is not forgotten. Int. J. Environ. Res. Public Health 21, 1141. doi: 10.3390/ijerph21091141 39338024 PMC11431549

[B72] de Maistre S. Gaillard S. Martin J. C. (2020). Cecal metabolome fingerprint in a rat model of decompression sickness with neurological disorders. Sci. Rep. 10, 15996. doi: 10.1038/s41598-020-73033-z 32994526 PMC7524739

[B73] Derion T. Guy H. J. Tsukimoto K. Schaffartzik W. Prediletto R. Poole D. C. . (1992). Ventilation-perfusion relationships in the lung during head-out water immersion. J. Appl. Physiol. (1985) 72, 64–72. doi: 10.1152/jappl.1992.72.1.64 1311293

[B74] Desola J. (1996). “ Descriptive classification of diving accidents, Treatment of decompression accidents in recreational diving”, in: 2nd European Consensus Conference (Marseille, France: European Committee for Hyperbaric Medicine).

[B75] Desruelle A. V. de Maistre S. Gaillard S. (2022). Cecal metabolomic fingerprint of unscathed rats: does it reflect the good response to a provocative decompression? Front. Physiol. 13, 882944. doi: 10.3389/fphys.2022.882944 35655958 PMC9152359

[B76] Diez-Silva M. Dao M. Han J. Lim C. T. Suresh S. (2010). Shape and biomechanical characteristics of human red blood cells in health and disease. MRS Bull. 35, 382–388. doi: 10.1557/mrs2010.571 21151848 PMC2998922

[B77] Di Muro G. Murphy F. G. Vann R. D. Howle L. E. (2020). Are interconnected compartmental models more effective at predicting decompression sickness risk? Inf. Med. Unlocked 20, 100334. doi: 10.1016/j.imu.2020.10033442574925

[B78] DMAC (2006). “ Guidance note D 021, rev 2. The Duration of Saturation Exposures and Surface Intervals following Saturations,” in Diving Medical Advisory Committee.

[B79] Doolette D. J. (2016). Venous gas emboli detected by two-dimensional echocardiography are an imperfect surrogate endpoint for decompression sickness. Diving Hyperb Med. 46, 4–10. 27044455

[B80] Doolette D. J. Gerth W. A. Gault K. A. (2011). “ Redistribution of decompression stop time from shallow to deep stops increases incidence of decompression sickness in air decompression dives,” in Navy Experimental Diving Unit, 11–06.

[B81] Doolette D. J. Mitchell S. J. (2022). Extended lifetimes of bubbles at hyperbaric pressure may contribute to inner ear decompression sickness during saturation diving. J. Appl. Physiol. (1985) 133, 517–523. doi: 10.1152/japplphysiol.00121.2022 35834629

[B82] Dorrington K. L. Balanos G. M. Talbot N. P. Robbins P. A. (2010). Extent to which pulmonary vascular responses to PCO2 and PO2 play a functional role within the healthy human lung. J. Appl. Physiol. (1985) 108, 1084–1096. doi: 10.1152/japplphysiol.90963.2008 20185627 PMC2867535

[B83] Dugrenot E. Guernec A. Orsat J. Guerrero F. (2024). Gene expression of Decompression Sickness-resistant rats through a miRnome/transcriptome crossed approach. Commun. Biol. 7, 1245. 10.1038/s42003-024-06963-3 PMC11446962 39358457

[B84] Dugrenot E. Orsat J. Guerrero F. (2022). Blood pressure in rats selectively bred for their resistance to decompression sickness. Diving Hyperb. Med. 52, 119–125. 10.28920/dhm52.2.119-125 PMC9522594 35732284

[B85] Dugrenot E. Orsat J. Gouin E. Guerrero F. (2026). Changes in liver and lung antioxidant and mitochondrial biogenesis gene expression in decompression sickness-resistant rats. Physiol. Genomics 58, 243–251. 10.1152/physiolgenomics.00218.2025 42024651

[B86] Dujic Z. Duplancic D. Marinovic-Terzic I. Bakovic D. Ivancev V. Valic Z. . (2004). Aerobic exercise before diving reduces venous gas bubble formation in humans. J. Physiol. 555, 637–642. 10.1113/jphysiol.2003.059360 PMC1664874 14755001

[B87] Dujic Z. Obad A. Palada I. Ivancev V. Valic Z. (2006). Venous bubble count declines during strenuous exercise after an open sea dive to 30 m. Aviat. Space Environ. Med. 77, 592–596. 16780236

[B88] Dujić Z. Palada I. Obad A. Duplancić D. Baković D. Valic Z. (2005). Exercise during a 3-min decompression stop reduces postdive venous gas bubbles. Med. Sci. Sports Exerc 37, 1319–1323. 10.1249/01.mss.0000174892.27331.ce 16118578

[B89] Dunford R. G. Denoble P. D. Forbes R. Pieper C. F. Howle L. E. Vann R. D. (2020). A study of decompression sickness using recorded depth-time profiles. Undersea Hyperb Med. 47, 75–91. doi: 10.22462/01.03.2020.9 32176949

[B90] Dunford R. G. Vann R. D. Gerth W. A. Pieper C. F. Huggins K. Wacholtz C. . (2002). The incidence of venous gas emboli in recreational diving. Undersea Hyperb Med. 29, 247–259. 12797666

[B91] Eckmann D. M. Armstead S. C. (2006). Influence of endothelial glycocalyx degradation and surfactants on air embolism adhesion. Anesthesiology 105, 1220–1227. doi: 10.1097/00000542-200612000-00022 17122585

[B92] Eftedal I. Ljubkovic M. Flatberg A. Jorgensen A. Brubakk A. O. Dujic Z. (2013). Acute and potentially persistent effects of scuba diving on the blood transcriptome of experienced divers. Physiol. Genomics, 965–972. United States. 10.1152/physiolgenomics.00164.2012 23964024

[B93] Elliott D. H. Hallenbeck J. M. Bove A. A. (1974). Venous infarction of the spinal cord in decompression sickness. J. R. Nav. Med. Serv. 60, 66–71. doi: 10.1136/jrnms-60-66 4425249

[B94] Evans A. Walder D. N. (1969). Significance of gas micronuclei in the aetiology of decompression sickness. Nature 222, 251–252. doi: 10.1038/222251a0 5778390

[B95] Flook V. (2004). “ Excursion tables in saturation diving - decompression implications of current UK practice,” in Unimed Scientific Limited.

[B96] Flook V. (2011). Predictions from a mathematical model of decompression compared to Doppler scores. Undersea Hyperb Med. 38, 187–196. 21721352

[B97] Fock A. (2007). Deep decompression stops. Diving Hyperbaric Med. J. 37, 125.

[B98] Francis J. (1998). Mechanism of spinal cord injury in DCI. SPUMS J. 28, 29–41. doi: 10.53347/rid-31239

[B99] Francis T. J. Pearson R. R. Robertson A. G. Hodgson M. Dutka A. J. Flynn E. T. (1988). Central nervous system decompression sickness: latency of 1070 human cases. Undersea Biomed. Res. 15, 403–417. doi: 10.53347/rid-95147 3067433

[B100] Gabler-Smith M. K. Westgate A. J. Koopman H. N. (2020). Microvessel density, lipid chemistry and N2 solubility in human and pig adipose tissue. Undersea Hyperb Med. 47, 1–12. doi: 10.22462/01.03.2020.25 32176941

[B101] Gale L. R. Chen Y. Hills B. A. Crawford R. (2007). Boundary lubrication of joints: Characterization of surface-active phospholipids found on retrieved implants. Acta orthopaedica 78, 309–314. doi: 10.1080/17453670710013852 17611842

[B102] García E. Mitchell S. J. (2020). Bubbles in the skin microcirculation underlying cutis marmorata in decompression sickness: Preliminary observations. Diving Hyperb Med. 50, 173–177. 10.28920/dhm50.2.173-177 PMC7481116 32557421

[B103] Gaustad S. E. Kondratiev T. V. Eftedal I. Tveita T. (2021). Effects of cold decompression on hemodynamic function and decompression sickness risk in a dry diving rat model. Front. Physiol. 12, 763975. doi: 10.3389/fphys.2021.763975 34803743 PMC8595113

[B104] Gempp E. Blatteau J. E. (2009). Decompression sickness with a right-to-left shunt. Clin. J. Sport Med., 512–513. doi: 10.1097/jsm.0b013e3181bd1df9 19898086

[B105] Gempp E. Blatteau J. E. Pontier J. M. Balestra C. Louge P. (2009a). Preventive effect of pre-dive hydration on bubble formation in divers. Br. J. Sports Med. 43, 224–228. doi: 10.1136/bjsm.2007.043240 18308884

[B106] Gempp E. Blatteau J. E. Stephant E. Louge P. (2009b). Relation between right-to-left shunts and spinal cord decompression sickness in divers. Int. J. Sports Med. 34, 150–153. doi: 10.1055/s-2008-1038844 18773377

[B107] Gempp E. Louge P. (2013). Inner ear decompression sickness in scuba divers: a review of 115 cases. Eur. Arch. Oto-Rhino-Laryngology Off. J. Eur. Fed. Oto-Rhino-Laryngological Societies 270, 1831–1837. doi: 10.1007/s00405-012-2233-y 23100085

[B108] Gempp E. Lyard M. Louge P. (2017). Reliability of right-to-left shunt screening in the prevention of scuba diving related-decompression sickness. Int. J. Cardiol. 248, 155–158. doi: 10.1016/j.ijcard.2017.08.059 28864135

[B109] Gempp E. Peny C. (2016). Yo-Yo diving and risk of decompression sickness in trainee military divers. J. Occup. Environ. Med. 58, e336–e337. doi: 10.1097/jom.0000000000000844 27608154

[B110] Gennser M. Blogg S. L. Eiken O. Mekjavic I. B. (2018). Indices of increased decompression stress following long-term bed rest. Front. Physiol. 9, 442. doi: 10.3389/fphys.2018.00442 30072904 PMC6058089

[B111] Germonpre P. Balestra C. (2017). Preconditioning to reduce decompression stress in scuba divers. Aerosp. Med. Hum. Perform. 88, 114–120. 10.3357/AMHP.4642.2017 28095955

[B112] Germonpre P. Balestra C. Obeid G. Caers D. (2015). Cutis marmorata skin decompression sickness is a manifestation of brainstem bubble embolization, not of local skin bubbles. Med. Hypotheses 85, 863–869. doi: 10.1016/j.mehy.2015.09.022 26432631

[B113] Germonpré P. Dendale P. Unger P. Balestra C. (1998). Patent foramen ovale and decompression sickness in sports divers. J. Appl. Physiol. (1985) 84, 1622–1626. 10.1152/jappl.1998.84.5.1622 9572808

[B114] Germonpre P. Lafere P. Portier W. Germonpre F. L. Marroni A. Balestra C. (2021). Increased risk of decompression sickness when diving with a right-to-left shunt: Results of a prospective single-blinded observational study (The "Carotid Doppler" Study). Front. Physiol. 12, 763408. 10.3389/fphys.2021.763408 PMC8586212 34777020

[B115] Germonpré P. Pontier J. M. Gempp E. Blatteau J. E. Deneweth S. Lafère P. . (2009). Pre-dive vibration effect on bubble formation after a 30-m dive requiring a decompression stop. Aviat. Space Environ. Med. 80, 1044–1048. 10.3357/asem.2588.2010 20027852

[B116] Gerriets T. Tetzlaff K. Hutzelmann A. Liceni T. Kopiske G. Struck N. . (2003). Association between right-to-left shunts and brain lesions in sport divers. Aviat. Space Environ. Med. 74, 1058–1060. 14556567

[B117] Gillian B. C. (1991). “ Evaluation of decompression sickness incidence in multi-day repetitive diving for 77,680 sport dives”, in: Repetitive Diving Workshop (Durham, NC, USA: American Association of Underwater Sciences, Duke University Medical Center).

[B118] Goldman S. (2010). Free energy wells for small gas bubbles in soft deformable materials. J. Chem. Phys. 132, 164509. doi: 10.1063/1.3394940 20441290

[B119] Goldman S. Solano-Altamirano M. Ledez K. (2017). Gas Bubble Dynamics in the Human Body (New-York, USA: Academic Press).

[B120] Gottschalk F. Eiken O. Elia A. Gennser M. (2023). Eccentric exercise 24 h prior to hypobaric decompression increases decompression strain. Eur. J. Appl. Physiol. 123, 2001–2011. doi: 10.1007/s00421-023-05214-3 37140728 PMC10460726

[B121] Hallenbeck J. M. Bove A. A. Elliott D. H. (1975). Mechanisms underlying spinal cord damage in decompression sickness. Neurology 25, 308–316. doi: 10.1212/wnl.25.4.308 1168317

[B122] Hartig F. Reider N. Sojer M. Hammer A. Ploner T. Muth C. M. . (2020). Livedo racemosa - The pathophysiology of decompression-associated cutis marmorata and right/left shunt. Front. Physiol. 11, 994. doi: 10.3389/fphys.2020.00994 33013436 PMC7497564

[B123] Harvey E. N. Barnes D. McElroy W. D. Whiteley A. H. Pease D. Cooper K. (1944). Bubble formation in animals. I. Physical factors. Journal of Cellular and Comparative Physiology 24, 1–22. doi: 10.1002/jcp.1030240102

[B124] Hempleman H. Crocker W. Taylor H. (1952). “ Investigation into the decompression tables. Report III. Part A. A New Theoretical Basis for the Calculation of Decompression Tables. Part B. A Method of Calculating Decompression Stages and the Formulation of New Diving Tables,” in Rnp. Royal Navy, Alverstoke. 52, 708.

[B125] Hennessy T. R. (1971). Inert gas diffusion in heterogeneous tissue I: without perfusion. Bull. Math. Biophys. 33, 235–248. doi: 10.1007/bf02579476 5088454

[B126] Hennessy T. R. (1974). The interaction of diffusion and perfusion in homogenous tissue. Bull. Math. Biol. 36, 505–526. doi: 10.1016/s0092-8240(74)80046-7 4457196

[B127] Hennessy T. R. (1989a). “ On the site of origin, evolution and effects of decompression microbubbles”, in: International symposium on supersaturation and bubble formation in fluids and organisms (Trondheim, Norway: Tapir Publishers), 291–340.

[B128] Hennessy T. R. (1989b). “ Modelling the transport of microbubbles across the pulmonary capillary bed,” in Sintef. Trondheim, Norway: SINTEF.

[B129] Hennessy T. R. Hempleman H. V. (1977). An examination of the critical released gas volume concept in decompression sickness. Proc. R. Soc Lond. B. Biol. Sci. 197, 299–313. doi: 10.1098/rspb.1977.0072 19749

[B130] Hess H. W. Wheelock C. E. St James E. Stooks J. L. Clemency B. M. Hostler D. (2021). Variability in venous gas emboli following the same dive at 3,658 meters. Undersea Hyperb Med. 48, 469–476. doi: 10.22462/07.08.2021.11 34847312

[B131] Hild M. Schmidt R. (1999). The mechanism of the collision-induced enhancement of the aΔg → X3Σg- and b1Σg+ → a1Δg radiative transitions of O2. J. Phys. Chem. A 103, 6091–6096.

[B132] Hills B. A. (1968). Linear bulk diffusion into heterogenous tissue. Bull. Math. Biophys. 30, 47–59. doi: 10.1007/bf02476939 5666819

[B133] Hills B. A. (1971). Decompression sickness: A fundamental study of "surface excursion" diving and the selection of limb bends versus C.N.S. symptoms. Aerosp. Med. 42, 833–836. 5098573

[B134] Hills B. A. (1977). Decompression Sickness, Vol. 1 (New York: John Wiley & Sons).

[B135] Hills B. A. (1979). Mechanical vs. ischemic mechanisms for decompression sickness. Aviat. Space Environ. Med. 50, 363–367. 464957

[B136] Hills B. A. (1989). Oligolamellar lubrication of joints by surface active phospholipid. J. Rheumatol. 16, 82–91. 2716011

[B137] Hills B. A. (1992a). A hydrophobic oligolamellar lining to the vascular lumen in some organs. Undersea Biomed. Res. 19, 107–120. 1561717

[B138] Hills B. A. (1992b). Spinal decompression sickness: the occurrence of lamellar bodies in spinal tissue as potential foci for bubble formation. SPUMS J. 22, 71.

[B139] Hills B. A. (1994). Release of surfactant and a myelin proteolipid apoprotein in spinal tissue by decompression. Undersea Hyperb Med. 21, 95–102. 7520309

[B140] Hills B. A. Butler B. D. (1978). The kangaroo rat as a model for type I decompression sickness. Undersea Biomed. Res. 5, 309–321. 734799

[B141] Hills B. A. Butler B. D. (1984). Surfactants identified in synovial fluid and their ability to act as boundary lubricants. Ann. Rheumatic Dis. 43, 641–648. doi: 10.1136/ard.43.4.641 6476922 PMC1001427

[B142] Hills B. A. James P. B. (1982). Spinal decompression sickness: mechanical studies and a model. Undersea Biomed. Res. 9, 185–201. 7135631

[B143] Hills B. A. James P. B. (1991). Microbubble damage to the blood-brain barrier: relevance to decompression sickness. Undersea Biomed. Res. 18, 111–116. 2042262

[B144] Hills B. A. LeMessurier D. H. (1969). Unsaturation in living tissue relative to the pressure and composition of inhaled gas and its significance in decompression theory. Clin. Sci. 36, 185–195. 5772098

[B145] Homer L. D. Weathersby P. K. (1986). How well mixed is inert gas in tissues? J. Appl. Physiol. (1985) 60, 2079–2088. doi: 10.1152/jappl.1986.60.6.2079 3013830

[B146] Howle L. E. Weber P. W. Hada E. A. Vann R. D. Denoble P. J. (2017). The probability and severity of decompression sickness. PloS One 12, e0172665. doi: 10.1371/journal.pone.0172665 28296928 PMC5351842

[B147] Hugon J. (2014). Decompression models: review, relevance and validation capabilities. Undersea Hyperb Med. 41, 531–556. 25562945

[B148] Hugon J. Barthelemy L. Rostain J. C. Gardette B. (2009). The pathway to drive decompression microbubbles from the tissues to the blood and the lymphatic system as a part of this transfer. Undersea Hyperb Med. 36, 223–236. 20088241

[B149] Imbert J. P . (1995). “ Evolution and offshore performances of the Comex Treatment Tables,” in Workshop on Decompression Illness Treatment ( Undersea Biomedical Hyperbaric Society, Palm Beach, FL, USA).

[B150] Imbert J. P. (1991). “ Decompression tables versus decompression procedures: an analysis of decompression sickness using diving databases”, in: 17th EUBS Annual Scientific Meeting (Heraklion, Greece: Diving and Hyperbaric Medicine Society).

[B151] Imbert J. P. (2005). “ Deep diving: the Comex experience,” in Long-Term Health Effects of Diving. The Godøysund 1993 Consensus Conference Revisited. Eds. Hope A. Risberg J. ( NUI AS, Bergen, Norway).

[B152] Imbert J. P. Barbaud A. Stevens S. Miller C. Peace H. Rossin H. . (2024a). Evaluation of North Sea saturation procedures through divers monitoring. Int. Marit. Health 75, 89–102. 10.5603/imh.99606 38949219

[B153] Imbert J. P. Bontoux M . (1987). “ Safety analysis of the French 1974 air decompression tables,” in Decompression in Surface-Based Diving. Undersea Hyperbaric Medical Society, Tokyo, Japan.

[B154] Imbert J. P. Bontoux M. (1988). “ Diving data bank: a unique tool for diving procedures development”, in: 20th Annual Offshore Technology Conference (Houston, Texas, USA: Offshore Technology Conference).

[B155] Imbert J. P. Egi S. M. Germonpre P. Balestra C. (2019). Static metabolic bubbles as precursors of vascular gas emboli during divers' decompression: a hypothesis explaining bubbling variability. Front. Physiol. 10, 807. doi: 10.3389/fphys.2019.00807 31354506 PMC6638188

[B156] Imbert J. P. Egi S. M. Ozyigit T. Balestra C. (2014). “ Analysis of 605 commercial diving DCS logs: trends and underlying mechanisms”, in: 40th EUBS Annual Scientific Meeting (Wiesbaden, Germany: European Undersea Biomedical Society).

[B157] Imbert J. P. Fructus X. Montbarbon S. (1992). “ Short and repetitive decompressions in air diving procedures: the commercial diving experience”, in: Repetitive Diving Workshop (Costa Mesa, CA, USA: American Academy of Underwater Sciences).

[B158] Imbert J. P. Matity L. Massimelli J. Y. Bryson P. (2024b). Review of saturation decompression procedures used in commercial diving. Diving Hyperb Med. 54, 23–38. doi: 10.28920/dhm54.1.23-38 38507907 PMC11065503

[B159] Imbert J. P. Sidali A. Butler A. Lucas J. (2026). Principles for the design, validation, and acceptance of decompression procedures. Undersea Hyperb Med. 53, 349–362. doi: 10.22462/855 42365958

[B160] James P. B. (1982). “ The size distribution of gas emboli arising during decompression. A review of the concept of critical diameter of gas emboli”, in: XIIIth Annual Congress, European Underwater Biomedical Society (Lubeck, Germany).

[B161] James P. B. (2014). Oxygen and the Brain: The Journey of Our Lifetime. The Key Research Relating Free Radical Formation to Hypoxia (North Palm Beach, FL, USA: Best Publishing Company).

[B162] Jankowski L. W. Tikuisis P. Nishi R. Y. (2004). Exercise effects during diving and decompression on postdive venous gas emboli. Aviat. Space Environ. Med. 75, 489–495. 15198273

[B163] Ji H. L. Zhao R. Matalon S. Matthay M. A. (2020). Elevated Plasmin(ogen) as a common risk factor for COVID-19 susceptibility. Physiol. Rev., 1065–1075. doi: 10.1152/physrev.00013.2020 32216698 PMC7191627

[B164] Jitsuiki K. Kushida Y. Nishio R. Yanagawa Y. (2021). Gas in joints after diving: Computed tomography may be useful for diagnosing decompression sickness. Wilderness Environ. Med. 32, 70–73. doi: 10.1016/j.wem.2020.09.006 33309396

[B165] Juan C. A. Pérez de la Lastra J. M. Plou F. J. Pérez-Lebeña E. (2021). The chemistry of reactive oxygen species (ROS) revisited: Outlining their role in biological macromolecules (DNA, lipids and proteins) and induced pathologies. Int. J. Mol. Sci. 22. doi: 10.3390/ijms22094642 33924958 PMC8125527

[B166] Kaczerska D. Siermontowski P. Olszański R. Krefft K. Małgorzewicz S. Van Damme-Ostapowicz K. (2013). The influence of high-fat diets on the occurrence of decompression stress after air dives. Undersea Hyperb Med. 40, 487–497. doi: 10.12775/jehs.2020.10.09.021 24377191

[B167] Kemper T. C. Rienks R. van Ooij P. J. van Hulst R. A. (2015). Cutis marmorata in decompression illness may be cerebrally mediated: a novel hypothesis on the aetiology of cutis marmorata. Diving Hyperb Med. 45, 84–88. 26165529

[B168] Kim D. J. Han J. W. (2022). Latent class analysis of decompression sickness symptoms of women divers. Healthcare (Basel) 10, 1246. doi: 10.3390/healthcare10071246 35885774 PMC9317702

[B169] Knauth M. Ries S. Pohimann S. Kerby T. Forsting M. Daffertshofer M. . (1997). Cohort study of multiple brain lesions in sport divers: role of a patent foramen ovale. Bmj 314, 701–705. doi: 10.1136/bmj.314.7082.701 9116544 PMC2126163

[B170] Komdeur P. Wingelaar T. T. van Hulst R. A. (2021). A survey on the health status of Dutch scuba diving instructors. Diving Hyperb Med. 51, 18–24. doi: 10.28920/dhm51.1.18-24 33761537 PMC8313785

[B171] Kot J. Sicko Z. Doboszynski T. (2015). The extended oxygen window concept for programming saturation decompressions using air and nitrox. PloS One 10, e0130835. doi: 10.1371/journal.pone.0130835 26111113 PMC4482426

[B172] Kumar M. A. Baba S. K. Sadida H. Q. Marzooqi S. A. Jerobin J. Altemani F. H. . (2024). Extracellular vesicles as tools and targets in therapy for diseases. Signal. Transduct Target Ther. 9, 27. doi: 10.1038/s41392-024-01735-1 38311623 PMC10838959

[B173] Lambertsen C. J. Idicula J. (1975). A new gas lesion syndrome in man, induced by "isobaric gas counterdiffusion. J. Appl. Physiol. 39, 434–443. doi: 10.1152/jappl.1975.39.3.434 170242

[B174] Lambrechts K. Germonpre P. Vandenheede J. Delorme M. Lafere P. Balestra C. (2022). Mini trampoline, a new and promising way of SCUBA diving preconditioning to reduce vascular gas emboli? Int. J. Environ. Res. Public Health 19, 5410. doi: 10.3390/ijerph19095410 35564805 PMC9105492

[B175] Lambrechts K. Pontier J. M. Mazur A. Buzzacott P. Morin J. Wang Q. . (2013). Effect of decompression-induced bubble formation on highly trained divers microvascular function. Physiol. Rep. 1, e00142. doi: 10.1002/phy2.142 24400144 PMC3871457

[B176] Lautridou J. Buzzacott P. Belhomme M. Dugrenot E. Lafere P. Balestra C. . (2017). Evidence of heritable determinants of decompression sickness in rats. Med. Sci. Sports Exerc 49, 2433–2438. doi: 10.1249/mss.0000000000001385 28731987

[B177] Le D. Q. Dayton P. A. Tillmans F. Freiberger J. J. Moon R. E. Denoble P. . (2021). Ultrasound in decompression research: fundamentals, considerations, and future technologies. Undersea Hyperb Med. 48, 59–72. doi: 10.22462/01.03.2021.8 33648035

[B178] Lee H. J. Lim D. S. Lee J. Lee D. G. Oh M. Y. Park J. . (2023). Decompression illness in divers with or without patent foramen ovale : a cohort study. Ann. Intern. Med. 176, 934–939. doi: 10.7326/m23-0260 37429031

[B179] Lehner C. E. Adams W. M. Dubielzig R. R. Palta M. Lanphier E. H. (1997). Dysbaric osteonecrosis in divers and caisson workers. An animal model. Clin. Orthop. Relat. Res. (344), 320–32. doi: 10.1097/00003086-199711000-00033 9372784

[B180] Letellier P. Turmine M. (2019). Bubble solution description by non-extensive thermodynamics: pressure effect. Chemphyschem A. Eur. J. Chem. Phys. Phys. Chem. 20, 2230–2235. doi: 10.1002/cphc.201900412 31328380

[B181] Ljubkovic M. Marinovic J. Obad A. Breskovic T. Gaustad S. E. Dujic Z. (2010). High incidence of venous and arterial gas emboli at rest after trimix diving without protocol violations. J. Appl. Physiol. (1985) 109, 1670–1674. doi: 10.1152/japplphysiol.01369.2009 20813975

[B182] MacLaughlin K. J. Barton G. P. Braun R. K. MacLaughlin J. E. Lamers J. J. Marcou M. D. . (2023). Hyperbaric air mobilizes stem cells in humans; a new perspective on the hormetic dose curve. Front. Neurol. 14, 1192793. doi: 10.3389/fneur.2023.1192793 37409020 PMC10318163

[B183] Madden L. A. Chrismas B. C. Mellor D. Vince R. V. Midgley A. W. McNaughton L. R. . (2010). Endothelial function and stress response after simulated dives to 18 msw breathing air or oxygen. Aviat. Space Environ. Med. 81, 41–45. doi: 10.3357/asem.2610.2010 20058736

[B184] Madden D. Ljubkovic M. Dujic Z. (2015). Intrapulmonary shunt and SCUBA diving: another risk factor? Echocardiography 32 Suppl 3, S205–S210. doi: 10.1111/echo.12815 25693625

[B185] Madden D. Lozo M. Dujic Z. Ljubkovic M. (2013). Exercise after SCUBA diving increases the incidence of arterial gas embolism. J. Appl. Physiol. (1985) 115, 716–722. doi: 10.1152/japplphysiol.00029.2013 23766500

[B186] Madden D. Thom S. R. Dujic Z. (2016). Exercise before and after SCUBA diving and the role of cellular microparticles in decompression stress. Med. Hypotheses 86, 80–84. doi: 10.1016/j.mehy.2015.12.006 26804603

[B187] Magri K. Eftedal I. Petroni Magri V. Matity L. Azzopardi C. P. Muscat S. . (2021). Acute effects on the human peripheral blood transcriptome of decompression sickness secondary to scuba diving. Front. Physiol. 12, 660402. doi: 10.3389/fphys.2021.660402 34177613 PMC8222921

[B188] Mapleson W. W. (1963). An electric analogue for uptake and exchange of inert gases and other agents. J. Appl. Physiol. 18, 197–204. doi: 10.1152/jappl.1963.18.1.197 13932730

[B189] Marroni A. Bennett P. B. Cronje F. J. Cali-Corleo R. Germonpre P. Pieri M. . (2004). A deep stop during decompression from 82 fsw (25 m) significantly reduces bubbles and fast tissue gas tensions. Undersea Hyperb Med. 31, 233–243. 15485086

[B190] Marroni A. Pieri M. Imbert J.-P. Pelliccia R. Arena M. Marroni L. . (2026). Deep versus shallow decompression stops: illustration of different decompression strategies used in technical diving. Int. Maritime Health. doi: 10.5603/imh.106791 42578735

[B191] Martin J. D. Thom S. R. (2002). Vascular leukocyte sequestration in decompression sickness and prophylactic hyperbaric oxygen therapy in rats. Aviat. Space Environ. Med. 73, 565–569. 12056672

[B192] Marzella L. Yin A. (1994). Role of extravascular gas bubbles in spinal cord injury induced by decompression sickness in the rat. Exp. Mol. Pathol. 61, 16–23. doi: 10.1006/exmp.1994.1022 7995376

[B193] Matalon S. Bartoszewski R. Collawn J. F. (2015). Role of epithelial sodium channels in the regulation of lung fluid homeostasis. Am. J. Physiol. Lung Cell. Mol. Physiol., L1229–L1238. doi: 10.1152/ajplung.00319.2015 26432872 PMC4669342

[B194] Mcdiarmid J. I. Ross J. A. Taylor C. L. Watt S. J. Adie W. (2004). “ Examination of the long-term health impact of diving: the ELTHI diving study. Co-ordinated investigation into the possible long-term health effects of diving at work,” in Uk Health & Safety Executive.

[B195] Millar I. (2019). “ Hypothetical mechanism for post-saturation diving cognitive impairment”, in: Bergen International Divng Seminar (Bergen, Norway: NUI AS).

[B196] Mitchell S. J. (2024). Decompression illness: a comprehensive overview. Diving Hyperb Med. 54, 1–53. doi: 10.28920/dhm54.1.suppl.1-53 38537300 PMC11168797

[B197] Mitchell S. J. Bennett M. H. Moon R. E. (2022). Decompression sickness and arterial gas embolism. N. Engl. J. Med. 386, 1254–1264. doi: 10.1056/nejmra2116554 35353963

[B198] Mitchell S. J. Doolette D. J. (2015). Pathophysiology of inner ear decompression sickness: potential role of the persistent foramen ovale. Diving Hyperb Med. 45, 105–110. 26165533

[B199] Moon R. E. Camporesi E. M. Kisslo J. A. (1989). Patent foramen ovale and decompression sickness in divers. Lancet 1, 513–514. doi: 10.1016/s0140-6736(89)90064-0 2564057

[B200] Mrakic-Sposta S. Vezzoli A. D'Alessandro F. Paganini M. Dellanoce C. Cialoni D. . (2020). Change in oxidative stress biomarkers during 30 days in saturation dive: A pilot study. Int. J. Environ. Res. Public Health 17. doi: 10.3390/ijerph17197118 32998440 PMC7579105

[B201] Murtagh F. Legendre P. (2011). “ Ward's hierarchical clustering method: clustering criterion and agglomerative algorithm,” in Arxiv Preprint Arxiv:1111.6285.

[B202] NAVSEA (2016). Us Navy Diving Manual, Revision 7. Navsea 0910-Lp-115-1921 (Washington, DC, 20350).

[B203] Nishi R. Y. Brubakk A. O. Eftedhal O. S. (2003). “ Bubble detection,” in Physiology and Medicine of Diving 5th Ed. Eds. Brubakk A. O. Neumann T. S. ( Saunders, London, UK), 501–529.

[B204] Obad A. Marinovic J. Ljubkovic M. Breskovic T. Modun D. Boban M. . (2010). Successive deep dives impair endothelial function and enhance oxidative stress in man. Clin. Physiol. Funct. Imaging 30, 432–438. doi: 10.1111/j.1475-097x.2010.00962.x 20718805

[B205] Orsat J. Gouin E. Guernec A. Balestra C. Theron M. Leman G. . (2026). Differential gene transcription following intravenous injection of air bubbles in rats with varying resistance to decompression sickness. Physiol. Genomics 58, 304–318. doi: 10.1152/physiolgenomics.00082.2026 42262931

[B206] Ozyigit T. Egi S. M. Denoble P. Balestra C. Aydin S. Vann R. . (2010). Decompression illness medically reported by hyperbaric treatment facilities: cluster analysis of 1929 cases. Aviat. Space Environ. Med. 81, 3–7. doi: 10.3357/asem.2495.2010 20058731

[B207] Papadopoulou V. Eckersley R. J. Balestra C. Karapantsios T. D. Tang M. X. (2013). A critical review of physiological bubble formation in hyperbaric decompression. Adv. Colloid Interface Sci. 191-192, 22–30. doi: 10.1016/j.cis.2013.02.002 23523006

[B208] Papadopoulou V. Evgenidis S. Eckersley R. J. Mesimeris T. Balestra C. Kostoglou M. . (2015). Decompression induced bubble dynamics on ex vivo fat and muscle tissue surfaces with a new experimental set up. Colloids Surf. B. Biointerfaces 129, 121–129. doi: 10.1016/j.colsurfb.2015.03.027 25835147

[B209] Papadopoulou V. Germonpré P. Cosgrove D. Eckersley R. J. Dayton P. A. Obeid G. . (2018). Variability in circulating gas emboli after a same scuba diving exposure. Eur. J. Appl. Physiol. 118, 1255–1264. doi: 10.1007/s00421-018-3854-7 29616324

[B210] Peppas S. Palaiodimos L. Nagraj S. Kokkinidis D. G. Tiwari N. Kharawala A. . (2023). Right-to-left shunt in divers with neurological decompression sickness: a systematic review and meta-analysis. Healthcare (Basel) 11. doi: 10.3390/healthcare11101407 37239692 PMC10217987

[B211] Pontier J. M. Blatteau J. E. Vallee N. (2008). Blood platelet count and severity of decompression sickness in rats after a provocative dive. Aviat. Space Environ. Med. 79, 761–764. doi: 10.3357/asem.2299.2008 18717114

[B212] Pontier J. M. Gempp E. Ignatescu M. (2012). Blood platelet-derived microparticles release and bubble formation after an open-sea air dive. Appl. Physiol. Nutr. Metab. 37, 888–892. doi: 10.1139/h2012-067 22735037

[B213] Pyle R. (1997). The importance of deep safety stops: rethinking ascent patterns from decompression dives. SPUMS J. 27, 113–115.

[B214] Pyle R. L. (2001). “ Insights on deep bounce dive safety from the technical diving community”, in: 16th Annual Meeting, United States-Japan Cooperative Program on Natural Ressources (UNR) (Honolulu, Hawai).

[B215] Rashbass C. (1955). “ Investigation into the decompression tables. Report VI. New tables,” in Rnprc(London).

[B216] Risberg J. (2021). Acclimatization to diving: a systematic review. Undersea Hyperb Med. 48, 127–147. doi: 10.22462/03.04.2021.3 33975403

[B217] Rosén A. Gennser M. Oscarsson N. (2022). Protein tau concentration in blood increases after SCUBA diving: an observational study. Eur. J. Appl. Physiol. 122, 993–1005. 10.1007/s00421-022-04892-9 PMC8926952 35142945

[B218] Salvagno M. Sterchele E. D. Zaccarelli M. Mrakic-Sposta S. Welsby I. J. Balestra C. . (2024). Oxidative stress and cerebral vascular tone: The role of reactive oxygen and nitrogen species. Int. J. Mol. Sci. 25, 3007. doi: 10.3390/ijms25053007 38474253 PMC10931760

[B219] Schellart N. A. Corstius J. J. Germonpré P. Sterk W. (2008). Bubble formation after a 20-m dive: deep-stop vs. shallow-stop decompression profiles. Aviat. Space Environ. Med. 79, 488–494. doi: 10.3357/asem.2164.2008 18500045

[B220] Schellart N. A. Sterk W. (2012). Venous gas embolism after an open-water air dive and identical repetitive dive. Undersea Hyperb Med. 39, 577–587. 22400448

[B221] Schirato S. R. El-Dash I. El-Dash V. Bizzarro B. Marroni A. Pieri M. . (2020). Association between heart rate variability and decompression-induced physiological stress. Front. Physiol. 11, 743. doi: 10.3389/fphys.2020.00743 32714210 PMC7351513

[B222] Schirato S. R. El-Dash I. El-Dash V. Bizzarro B. Marroni A. Pieri M. . (2024). A broad appraisal of decompression-induced physiological stress in different simulated dive profiles. J. Clin. Trans. Res. 10. doi: 10.36922/jctr.24.00021

[B223] Schirato S. R. Silva V. Iadocicco K. Maronni A. Pieri M. Cialoni D. . (2022). Post-decompression bubble and inflammation interactions: a non-extensive dynamical system model. Undersea Hyperb Med. 49, 207–226. doi: 10.22462/03.04.2022.6 35580488

[B224] Shields T. Duff P. Wilcock S. Giles R. (1989). “ Decompression sickness from commercial offshore air-diving operations on the UK continental shelf during 1982 to 1988,” in Subtech 89: Fitness for Purpose, SUT, Aberdeen, UK.

[B225] Shields T. G. Lee W. B. (1998). “ The incidence of decompression sickness arising from commercial offshore air-diving operations in the UK sector of the North Sea during 1982/83,” in Department of Energy, London, UK. Sheffield: Health and Safety Executive.

[B226] Srinivasan R. S. Gerth W. A. Powell M. R. (2000). A mathematical model of diffusion-limited gas bubble dynamics in tissue with varying diffusion region thickness. Respiration Physiol. 123, 153–164. doi: 10.1016/s0034-5687(00)00173-0 10996196

[B227] Strauss M. B. Lu L. Q. Miller S. S. (2018a). The Gradient Perfusion Model Part 1: Why and at what sites decompression sickness can occur. Undersea Hyperb Med. 45, 287–295. doi: 10.22462/05.06.2018.4 30028915

[B228] Strauss M. B. Lu L. Q. Miller S. S. (2018b). The Gradient Perfusion Model Part 2: Substantiation of the GPM with clinical cases. Undersea Hyperb Med. 45, 297–305. 30028916

[B229] Sureda A. Ferrer M. D. Batle J. M. Tauler P. Tur J. A. Pons A. (2009). Scuba diving increases erythrocyte and plasma antioxidant defenses and spares NO without oxidative damage. Med. Sci. Sports Exerc 41, 1271–1276. doi: 10.1249/mss.0b013e3181951069 19461538

[B230] Sykes O. Clark J. E. (2013). Patent foramen ovale and scuba diving: a practical guide for physicians on when to refer for screening. Extrem Physiol. Med. 2, 10. doi: 10.1186/2046-7648-2-10 23849539 PMC3710076

[B231] Thaler J. Pignel R. Magnan M. A. Pellegrini M. Louge P. (2020). Decompression illness treated at the Geneva hyperbaric facility 2010-2016: A retrospective analysis of local cases. Diving Hyperb Med. 50, 370–376. doi: 10.28920/dhm50.4.370-376 33325018 PMC8038901

[B232] Theron M. Blasselle A. Nedellec L. Ballet P. Dugrenot E. Gardette B. . (2025). N(2) exchanges in hyperbaric environments: toward a model based on physiological gas transport (O(2) and CO(2)). J. Appl. Physiol. (1985) 138, 342–357. doi: 10.1152/japplphysiol.00357.2024 39589899

[B233] Thom S. R. Bhopale V. M. Arya A. K. Ruhela D. Bhat A. R. Mitra N. . (2023a). Blood-borne microparticles are an inflammatory stimulus in type 2 diabetes mellitus. Immunohorizons 7, 71–80. doi: 10.4049/immunohorizons.2200099 36645851 PMC10563440

[B234] Thom S. R. Bhopale V. M. Bhat A. R. Arya A. K. Ruhela D. Qiao G. . (2023b). Neuroinflammation with increased glymphatic flow in a murine model of decompression sickness. J. Neurophysiol. 129, 662–671. doi: 10.1152/jn.00005.2023 36752495 PMC10010924

[B235] Thom S. R. Y. Bhopale V. M. Huang S. Milovanova T. N. (2011). Microparticles initiate decompression-induced neutrophil activation and subsequent vascular injuries. J. Appl. Physiol. 110, 340–351. doi: 10.1152/japplphysiol.00811.2010 20966192

[B236] Thom S. R. Bhopale V. M. Yang M. (2014). Neutrophils generate microparticles during exposure to inert gases due to cytoskeletal oxidative stress. J. Biol. Chem. 289, 18831–18845. doi: 10.1074/jbc.m113.543702 24867949 PMC4081925

[B237] Thom S. R. Bhopale V. M. Yang M. (2019). Microparticle-induced vascular injury in mice following decompression is inhibited by hyperbaric oxygen: effects on microparticles and interleukin-1beta. J. Appl. Physiol. (1985) 126, 1006–1014. doi: 10.1152/japplphysiol.01109.2018 30763157

[B238] Thom S. R. Bhopale V. M. Yu K. Huang W. Kane M. A. Margolis D. J. (2017). Neutrophil microparticle production and inflammasome activation by hyperglycemia due to cytoskeletal instability. J. Biol. Chem. 292, 18312–18324. doi: 10.1074/jbc.m117.802629 28972154 PMC5672053

[B239] Thom S. R. Bhopale V. M. Yu K. Yang M. (2018). Provocative decompression causes diffuse vascular injury in mice mediated by microparticles containing interleukin-1beta. J. Appl. Physiol. (1985) 125, 1339–1348. doi: 10.1152/japplphysiol.00620.2018 30113270

[B240] Thom S. R. Milovanova T. N. Bogush M. Bhopale V. M. Yang M. Bushmann K. . (2012). Microparticle production, neutrophil activation, and intravascular bubbles following open-water SCUBA diving. J. Appl. Physiol. (1985) 112, 1268–1278. doi: 10.1152/japplphysiol.01305.2011 22323646

[B241] Thom S. R. Yang M. Bhopale V. M. Milovanova T. N. Bogush M. Buerk D. G. (2013). Intramicroparticle nitrogen dioxide is a bubble nucleation site leading to decompression-induced neutrophil activation and vascular injury. J. Appl. Physiol. (1985) 114, 550–558. doi: 10.1152/japplphysiol.01386.2012 23264541

[B242] Thorsen T. Klausen H. Lie R. T. Holmsen H. (1993). Bubble-induced aggregation of platelets: effects of gas species, proteins, and decompression. Undersea Hyperb Med. 20, 101–119. 8392414

[B243] Togawa S. Maruyama M. Yamami N. Nakayama H. Shibayama M. Kawashima M. . (2006). Dissociation of neurological deficits in spinal decompression illness. Undersea Hyperb Med. 33, 265–270. 17004413

[B244] Torti S. R. Billinger M. Schwerzmann M. Vogel R. Zbinden R. Windecker S. . (2004). Risk of decompression illness among 230 divers in relation to the presence and size of patent foramen ovale. Eur. Heart J. 25, 1014–1020. doi: 10.1016/j.ehj.2004.04.028 15191771

[B245] Tournoy K. G. Vandebotermet M. Neuville P. Germonpré P. (2024). Modelling the risk factors for accidents in recreational divers: results from a cross-sectional evaluation in Belgium. Diving Hyperb Med. 54, 287–295. doi: 10.28920/dhm54.4.287-295 39675736 PMC12018701

[B246] UK Health & Safety Executive (1998). Diving Information Sheet No 5: Exposure limits for air diving operations. Sudbury, Suffolk, UK. doi: 10.1093/law/9780199287284.003.0012

[B247] Valadao J. M. Vigilante J. A. DiGeorge N. W. O'Connor S. E. Bear A. Kenyon J. . (2014). Ketogenic diet for high partial pressure oxygen diving. Undersea Hyperb Med. 41, 331–335. 25109086

[B248] Vallée N. Dugrenot E. Desruelle A. V. (2021). Evidence of a hormonal reshuffle in the cecal metabolome fingerprint of a strain of rats resistant to decompression sickness. Sci. Rep. 11, 8317. 10.1038/s41598-021-87952-y PMC8050073 33859311

[B249] Vallée N. Dugrenot E. Desruelle A. V. (2023). Highlighting of the interactions of MYD88 and NFKB1 SNPs in rats resistant to decompression sickness: toward an autoimmune response. Front. Physiol. 14, 1253856. 10.3389/fphys.2023.1253856 PMC10470123 37664439

[B250] Van Liew H. D. Burkard M. E. (1993). Density of decompression bubbles and competition for gas among bubbles, tissue, and blood. J. Appl. Physiol. (1985) 75, 2293–2301. doi: 10.1152/jappl.1993.75.5.2293 8307888

[B251] Van Liew H. D. Conkin J. Burkard M. E. (1993). The oxygen window and decompression bubbles: estimates and significance. Aviat. Space Environ. Med. 64, 859–865. 8216150

[B252] Van Liew H. D. Raychaudhuri S. (1997). Stabilized bubbles in the body: pressure-radius relationships and the limits to stabilization. J. Appl. Physiol. (1985) 82, 2045–2053. doi: 10.1152/jappl.1997.82.6.2045 9173975

[B253] Vann R. D. (1982). “ Decompression theory and decompression,” in The Physiology of Diving and Compressed Air Work. Eds. Bennett P. Elliott D. ( Bailliere and Tindall), 32–382.

[B254] Vann R. D. Denoble P. Uguccioni D. M. Pollock N. W. Freiberger J. J. Pieper C. F. . (2005). “ The risk of decompression sickness (DCS) is influenced by dive conditions”, in: Diving for Science, AAUS.

[B255] Vann R. D. Grimstad J. Nielsen C. H. (1980). Evidence for gas nuclei in decompressed rats. Undersea Biomed. Res. 7, 107–112. 7404848

[B256] Vezzoli A. Mrakic-Sposta S. Brizzolari A. Balestra C. Camporesi E. M. Bosco G. (2024). Oxy-inflammation in humans during underwater activities. Int. J. Mol. Sci. 25. doi: 10.3390/ijms25053060 38474303 PMC10931934

[B257] Vik A. Brubakk A. O. Hennessy T. R. Jenssen B. M. Ekker M. Slørdahl S. A. (1990). Venous air embolism in swine: transport of gas bubbles through the pulmonary circulation. J. Appl. Physiol. (1985) 69, 237–244. doi: 10.1152/jappl.1990.69.1.237 2203724

[B258] Vik A. Jenssen B. M. Brubakk A. O. (1993a). Arterial gas bubbles after decompression in pigs with patent foramen ovale. Undersea Hyperb Med. 20, 121–131. 8329939

[B259] Vik A. Jenssen B. M. Eftedal O. Brubakk A. O. (1993b). Relationship between venous bubbles and hemodynamic responses after decompression in pigs. Undersea Hyperb Med. 20, 233–248. 8401153

[B260] Wang Q. Guerrero F. Theron M. (2020). Pre-hydration strongly reduces decompression sickness occurrence after a simulated dive in the rat. Diving Hyperb Med. 50, 288–291. doi: 10.28920/dhm50.3.288-291 32957132 PMC7819728

[B261] Wilmshurst P. (2015). Cutis marmorata and cerebral arterai gas embolism. Diving Hyperbaric Med. 45. 26687315

[B262] Wilmshurst P. Bryson P. (2000). Relationship between the clinical features of neurological decompression illness and its causes. Clin. Sci. (Lond) 99, 65–75. doi: 10.1042/cs0990065 10887059

[B263] Wilmshurst P. T. Pearson M. J. Walsh K. P. Morrison W. L. Bryson P. (2001). Relationship between right-to-left shunts and cutaneous decompression illness. Clin. Sci. (Lond) 100, 539–542. doi: 10.1042/cs1000539 11294694

[B264] Wisloff U. Richardson R. S. Brubakk A. O. (2003). NOS inhibition increases bubble formation and reduces survival in sedentary but not exercised rats. J. Physiol. 546, 577–582. 10.1113/jphysiol.2002.030338 PMC2342529 12527743

[B265] Workman R. D. (1965). “ Calculation of decompression schedules for nitrogen-oxygen and helium-oxygen dives,” in Us Navy Exp Diving Unit. doi: 10.21236/ad0620879 5295231

[B266] Yang M. Milovanova T. N. Bogush M. Uzun G. Bhopale V. M. Thom S. R. (2012). Microparticle enlargement and altered surface proteins after air decompression are associated with inflammatory vascular injuries. J. Appl. Physiol. (1985) 112, 204–211. doi: 10.1152/japplphysiol.00953.2011 21960660 PMC3290415

[B267] Yount D. E. (1979). Application of a bubble formation model to decompression sickness in rats and humans. Aviat. Space Environ. Med. 50, 44–50. 217330

[B268] Yount D. E. (1982). On the evolution, generation, and regeneration of gas cavitation nuclei. J. Acoust. Soc Am. 71, 1473–1481. doi: 10.1121/1.387845

[B269] Yount D. E. Kunkle T. D. D'Arrigo J. S. Ingle F. W. Yeung C. M. Beckman E. L. (1977). Stabilization of gas cavitation nuclei by surface-active compounds. Aviat. Space Environ. Med. 48, 185–189. 856151

[B270] Zanchi J. Ljubkovic M. Denoble P. J. Dujic Z. Ranapurwala S. Pollock N. W. (2014). Influence of repeated daily diving on decompression stress. Int. J. Sports Med. 35, 465–468. doi: 10.1055/s-0033-1334968 23771833

